# Can laboratory-based XAFS compete with XRD and Mössbauer spectroscopy as a tool for quantitative species analysis? Critical evaluation using the example of a natural iron ore

**DOI:** 10.1371/journal.pone.0323678

**Published:** 2025-05-16

**Authors:** Sebastian Praetz, Christopher Schlesiger, Damian Alexander Motz, Stephen Klimke, Moritz Jahns, Christine Gottschalk, Lena Heinrich, Eva Maria Heppke, Wolfgang Malzer, Franz Renz, Carla Vogt, Birgit Kanngießer

**Affiliations:** 1 Technische Universität Berlin, Institute of Optics and Atomic Physics, Berlin, Germany; 2 Leibniz University Hannover, Institute of Inorganic Chemistry, Hannover, Germany; 3 Leibniz Institute of Freshwater Ecology and Inland Fisheries (IGB), Berlin, Germany; 4 Technische Universität Berlin, Institut für Chemie, Berlin, Germany; Purdue University, UNITED STATES OF AMERICA

## Abstract

While X-ray diffraction (XRD) is a commonly used method for quantification analysis using Rietveld refinement and quantitative Mössbauer spectroscopy is sporadically used primarily for iron speciation, laboratory X-ray Absorption Fine Structure Spectroscopy (lab-XAFS) is rarely applied for the quantitative determination of sample compositions. With the recent developments of laboratory-based XAFS spectrometers, this method becomes more interesting for many applications as well as for quantification. The goal of this study is to compare quantitative lab-XAFS via Linear Combination Fitting (LCF) of reference spectra with XRD and Mössbauer spectroscopy. Iron species analysis with the focus on the determination of the mass ratio alpha-iron(III) oxide (α-Fe_2_O_3_)/iron(II, III) oxide (Fe_3_O_4_) was used as an example. The examinations were performed on synthetic α-Fe_2_O_3_/Fe_3_O_4_ model mixtures and, predominantly, on a natural iron ore sample mainly consisting of the minerals hematite and magnetite, thus, these two iron oxides. For the iron K-edge lab-XAFS measurements an X-ray tube-based spectrometer using the von Hamos geometry with Highly Annealed Pyrolytic Graphite (HAPG) mosaic crystal optic was used. The capabilities and challenges of each method are discussed. The quantitative model mixtures examinations by lab-XAFS show results and accuracies similar to those obtained by XRD and Mössbauer spectroscopy. However, while the quantitative results for the iron ore investigations by lab-XAFS are in good agreement (deviation of 2 percent points) with the XRD results, the composition determined by Mössbauer spectroscopy differs clearly from the lab-XAFS and XRD results. Furthermore, the Mössbauer spectroscopic examinations hint the presence of an additional iron oxide species affecting the quantification. Besides the still common challenges in identification, differentiation and quantification of different iron oxides, the results show that quantitative lab-XAFS can particularly compete with quantitative XRD when determining the species composition of one element. This makes lab-XAFS particularly well-suited for routine analytics.

## Introduction

There are not too many options for the task of quantitative analysis of species composition in solid state samples in the laboratory. X-ray diffraction (XRD) with Rietveld analysis is the most common one. It is a mature method and well established. Mössbauer is an option for a selection of elements. Optical methods very much depend on sample properties. With the (commercial) availability of laboratory-based spectrometers for X-ray Absorption Fine Structure (XAFS) spectroscopy [1–10], a new option comes into play.

XAFS spectroscopy, including its derivatives X-ray Absorption Near Edge Structure (XANES) and Extended X-ray Absorption Fine Structure (EXAFS), was first measured in laboratory settings more than 100 years ago. Maurice de Broglie recorded the first absorption edge in 1913 [11], and in 1920, Fricke [12] and Hertz [13] were the first to observe the fine structure. However, the major breakthrough for XAFS came in the 1970s with the pioneering publications on EXAFS by Sayers, Stern and Lytle [14–16] and the development of synchrotron radiation sources, particularly at Stanford in 1974 [17]. Since then, XAFS at synchrotron sources has become a well-established and widely applied technique across various scientific fields*.* In contrast, its frequent use in laboratory-based spectrometers has been a topic since just some time and is still emerging as showing in various application fields, especially qualitative and quantitative species analyses [1,2,8,18–24]. In contrast to that, XRD in general and Mössbauer spectroscopy (gamma radiation resonance spectroscopy) especially in the field of investigations into iron containing compounds are well-known laboratory-based methods. While each method has its own rich information content, a common field for all three is the species analysis, i.e., the determination of different species of one element in a sample (different by means of oxidation states, electronic states, bond states and/ or crystal structure).

While laboratory-based XAFS is now in the process of being established side-by-side with these methods, it lacks their long history and tradition that lead not only to a huge amount of experience about the capabilities and limitations of each, but also to the existence of databases of vast amounts of reference data of structures, phases, etc. Obviously, this step still has to come for laboratory-based XAFS, even when there is a lot of knowledge from synchrotron-based experiments that will give a jumpstart. Here, this study will try to close some of the gaps by comparing all three methods in their respective laboratory-based installation. As XRD is the best-known method of those three, its introduction will be the shortest.

First, XRD, in the form of powder diffraction and single-crystal diffraction, has been applied in multiple scientific fields (e.g., chemistry, materials science, geology/ mineralogy and biology) in research as well as in industry since a long time [25–27]. It is based on an interference effect in the scattering of X-rays. As there is the need for a periodic structure to show significant interference pattern, this method is limited to crystalline structures, that, therefore, function as diffraction gratings [25,27]. This implies that classical XRD is primarily performed on solid macro- or microcrystalline materials [25]. Nanocrystalline materials or materials with a high number of crystal defects already cause a significant broadening of the reflections, which increases with the decrease of the particle size (this effect can be used for estimations of particle/ crystallite sizes via the Scherrer equation) or the increase of defects [25,27,28]. Materials with crystallite sizes of *d* ≤ 15–3 nm [25] (so-called X-ray amorphous substances) and amorphous materials (such as glasses) do not cause an X-ray diffraction pattern in regular XRD experiments and cannot be analyzed by them. Nevertheless, these materials can still be examined by small-angle X-ray scattering (SAXS), but SAXS is a special technique with relatively limited accessibility and, moreover, does not provide the same information as regular XRD does and is instead rather used for nanoparticle size or pore size determinations [18,20].

Second, Mössbauer spectroscopy is primarily established in the analytics of iron containing substances and thus in the form of ^57^Fe Mössbauer spectroscopy. Due to its extremely high sensitivity to energy changes of the nucleus (about 10^–8^ eV [29]) and hence to distinct chemical environments of the analyte atom (hyperfine interactions) ^57^Fe Mössbauer spectroscopy is used for detailed examinations of oxidation states, spin states and coordination polyhedra of iron atoms in many different compounds [29–35]. Furthermore, observations of the magnetic properties of iron compounds are also possible [29–35]. These iron compound characteristics are reflected in the presence or absence and the magnitudes of the three hyperfine parameters isomeric/ chemical shift *δ*, quadrupole splitting ∆*E*_Q_ and magnetic splitting *H* (or *z*) which can be observed in obtained Mössbauer spectra [29–35]. Hence, it is relatively common in coordination chemistry [31,36], material science [29,30,34], building materials science [37,38], geology [31,32], archaeometry [31,39] and even astrophysics [31,32]. One of the most famous applications was the successful in-situ ^57^Fe Mössbauer spectroscopy of the iron-rich Martian surface materials. These iron species analyses were carried out by the miniaturized Mössbauer spectrometers (*MIMOS II*) of the *NASA Mars Exploration Rovers* (MER) *Spirit* and *Opportunity* from 2004 to the 2010s, which were part of the large and still ongoing *NASA Mars Exploration Program* (MEP) and its missions [29,31,40]. Nevertheless, the overall establishment of Mössbauer spectroscopy is considerably more limited than the use of XRD, especially regarding routine analytics, for example product control and monitoring in industrial production, even though some applications in these analytic field exist [31]. Besides the strict rules and laws usually connected with the use of radioactive sources, one of the main restrictions is the limitation of Mössbauer spectroscopic analyses to a few elements in the periodic table and, thus, to substances which contain these elements. In addition to the Doppler effect applied on the gamma source to slightly modulate the radiation, the observed basic physical interaction is the Mössbauer effect, which is a recoilless emission/ resonant absorption of gamma radiation by atomic nuclei [29–31,41]. The recoil contribution occurring during the emission/ absorption due to momentum conservation is not negligible in case of the very high energy of gamma radiation in the order of *E* ≈ 10^1^–10^4^ keV [42] and the very small spectral line widths of the gamma lines [29,30]. Thus, this interaction requires very specific conditions of a nucleus [29,30] which limit the number of Mössbauer-active atomic nuclei or rather elements and isotopes to about 40 elements/ 80 isotopes [31,32,34]. The availability of appropriate gamma radiation sources required for the implementation of the technique decreases the number of elements effectively analyzable by Mössbauer spectroscopy to about 20 elements [31,32,34]. Of these elements iron, specifically the isotope ^57^Fe, is the most common one [29,32,34] (due to stability/ abundance of the Mössbauer-active isotope, gamma radiation energy of *E* = 14.4 keV [29,30,41], availability/ handling of the radiation source ^57^Co [29–31], frequent natural occurrence and numerous applications of iron/ iron-containing compounds [32,43,44]), though tin (^119^Sn) [29,34] and iridium (^191^Ir) [29,30,32] should also be mentioned. Another limitation of the technique is that, similar to XRD, Mössbauer spectroscopy also strictly requires the solid state of a sample, since this is also an essential condition for the recoilless resonant absorption (recoil energy dispersion) [29,30,41]. Nevertheless, in contrast to XRD, crystalline as well as amorphous solid samples can be analyzed by Mössbauer spectroscopy [29,33].

Third, XAFS is based on the energy-dependent absorption of X-rays in matter. The absorption, here the mass attenuation coefficient *µ*, is acquired by measuring the transmission through the sample (*I* with sample and *I*_0_ without the sample) and using the Lambert-Beer law to calculate *µQ* with *Q* as the mass deposition in the sample. This absorption strongly increases at binding energies of core electrons which yields to element specific absorption edges [45]. Additionally, X-ray absorption spectra show distinct fine structures in energy regions right in front of, at and above the edges (XANES region including pre-edges and edges) as well as in decreasing extent in regions farer behind the edges (EXAFS) [45]. These fine structures can contain simple features such as the presence, shapes and intensities of pre-edges (e.g., occurring in case of K-edges when absorber atoms have d orbital vacancies), shapes of edges and also slight energy shifts of both, pre-edges and edges [45–48]. Especially edge shifts reflect shifts/ differences in the binding energies of core electrons due to oxidation states and bond distances, though also pre-edge shifts can trace back to these effects (besides effects of the detailed d electron configuration) [45–48]. Furthermore, the fine structures also contain more complex features in form of oscillations in the XANES and EXAFS regions [45–48]. The ejected photoelectron can scatter at neighboring atoms, leading to a complex interference pattern that modulates the possibility of absorption [45–48]. Thus, not only the oxidation state by means of chemical shift of the binding energy, but also the complete chemical environment of the absorbing element is translated into a unique spectral absorption behavior. On the one hand, this can be evaluated to get structural and electronic information such as oxidation states, spin states, coordination geometries (coordination numbers and coordination polyhedra) of absorber atoms as well as and bond types, distances and angles between absorber and neighboring atoms [45–48]. On the other hand, this can also be taken as a fingerprint spectrum to determine species content in a linear fitting approach.

In this publication XRD and Mössbauer spectroscopy will be compared to a self-developed laboratory-XAFS spectrometer. In this work, the acronym XAFS is utilized to refer to the spectra, as they encompass the energy range of the XANES region and also include significant portions of the spectra that fall within the EXAFS region. It is important to note that no EXFAS analysis, such as Fourier transformation or EXAFS modelling, has been conducted, as this would exceed the scope of this study. Major points of the discussion will be transferable independent of the specific spectrometer, others will mirror our spectrometer’s specific performance and might differ for other laboratory-XAFS spectrometer approaches and setups. In contrast to Mössbauer spectroscopy and XRD, respectively, XAFS neither has the disadvantage of only being limited to a few elements in the periodic table nor only to observe solid (and especially crystalline) phases. Hence, in combination with the mentioned continuous developments and improvements of laboratory-XAFS spectrometers as complements to the established synchrotron setups XAFS has the large potential to become equally important as the other two in species analysis. However, since especially laboratory-XAFS lacks decades of development, there is still much work to be done to establish it in a manner similar to XRD and Mössbauer spectroscopy. That includes, e.g., determination of lower limits of detection and uncertainty analysis.

This study presents the direct comparison of quantitative analysis with laboratory-XAFS to XRD and Mössbauer spectroscopy on a model sample system. The focus was on the capability of quantitative species analyses in the form of determinations of relative species ratios. Qualitative analyses were also considered, as preceding qualitative evaluations are necessary requirements for and are part of quantifications. A Mexican iron ore, which mainly consisted of the minerals magnetite (Fe_3_O_4_) and hematite (α-Fe_2_O_3_), was measured with all three methods and evaluated quantitatively with respect to the species mass ratio *ω*_rel_(α-Fe_2_O_3_)/*ω*_rel_(Fe_3_O_4_). Pure synthetic alpha-iron(III) oxide (α-Fe_2_O_3_) and iron(II, III) oxide (Fe_3_O_4_) were used as reference substances. Additionally, three model mixtures with precisely determined compositions of these reference substances were also prepared and examined preliminary to the analyses of the natural iron ore sample. The goal of these preliminary investigations was to get general information on the accuracy (closeness/ distance between measured value and true value (ISO 5725–1) [49]) of each method regarding the species ratio quantification *ω*_rel_(α-Fe_2_O_3_)/*ω*_rel_(Fe_3_O_4_). A major focus is on laboratory XAFS as the new method among those three, but also on the comparison with the other two methods, not only by means of the quantitative results, but also the limitations, advantages, and challenges of each method**.**

## Experimental section

The following sections cover a short introduction to the specifics of the measurements of all three methods. The descriptions focus on the instrumental characteristics and settings as well as the applied sample preparation techniques. However, only the basic information on data evaluation and particularly the quantification procedures (XAFS: normalization + LCF, XRD: Rietveld refinement and Mössbauer spectroscopy: area ratio approach) is covered in this chapter and will be described more precisely in the results section instead. The reason for this is that the work with the different quantification approaches was essential part of this study and several choices as well as adjustments/ modification of the approaches have been made in consequence of/ in interplay with parts of the results. Furthermore, the sample system used, consisting of the two reference substances, the model mixtures and Mexican iron ore, as well as the results of the necessary pre-characterizations will be described in the following sections.

### X-ray absorption fine structure spectroscopy

The experimental setup for the laboratory XAFS spectroscopy is based on the Highly Annealed Pyrolytic Graphite (HAPG) von Hámos spectrometer with the use of a cylindrically shaped crystal for combining high spectral resolving power with a high solid angle of detection [4,50].

As detector unit the pixelated X-ray hybrid-CMOS detector *Dectris EIGER2 R 500k* was used. The area of detection is 77.3 mm x 38.6 mm with a pixel size of 75 µm x 75 µm. The X-ray source was a water-cooled micro focus X-ray tube with molybdenum as anode material, a power of 30 Watt optimized at 15 kV and a spot size of 70 µm.

For the setup specifics and more details on the used components, we refer to the mentioned publications [4,50].

Two common solid sample preparation techniques were considered and used in this study. In case of the pure reference substances iron(III) oxide (α-Fe_2_O_3_) and iron(II, III) oxide (Fe_3_O_4_) as well as the Mexican iron ore samples were applied on adhesive tape. To do so, the sample was applied via horsehair brush in between two tape layers. Afterwards the tape was sliced and pre-analyzed via X-ray fluorescence (XRF) to estimate the mass deposition *Q* of iron on each slice and also to ensure the mandatory homogeneity. The latter aspect is particularly crucial for quantitative evaluation of lab-XAFS spectra (the analyzed sample area is wide due to the large beam of the applied X-ray sources, thus, X-ray tubes in lab-based spectrometers). An adequate sample thickness for the absorption measurement is then achieved by stacking the required amount of sample slices to reach an edge jump of about *µQ* = 1. That can result in stacking up to 20–40 slices, depending on the properties of the powder material. In contrast, the α-Fe_2_O_3_/Fe_3_O_4_ model mixtures were prepared both via the adhesive tape and the wax pellet method. For the pellet preparation sample powder was mixed with a binder (in this case *Hoechst Wax C*©). The ratio of sample material and wax for pure iron oxide powder is about 1:6. The ratio was optimized to achieve a stable pellet and to avoid unnecessary absorption by the binder. Therefore, the ratio varies for different elements, different analyte concentration in the sample and for materials with different particle/preparation properties. The wax and sample materials were mixed in a plastic bottle in a vortex shaker with the addition of two inert glass (SiO_2_) balls of 5 mm diameter. The obtained sample-wax mixtures were then pressed into pellets by use of a hydraulic press. Finally, these pellets were embedded between adhesive tape stripes. The main reason for preparing the samples as wax pellets in addition to applying the material on adhesive tape was that the two phases of the mixtures might have different properties on adhesive tape and would, therefore, result in different fraction after preparation. When adding and mixing with wax in a vortex mixer and pressing the material in a pellet this application properties should have no effect. For the Mexican iron ore, although it was also a phase mixture, the deposition behavior should not differ between the two phases. The raw material was grinded with a ball mill (see below) and, thus, the Fe-oxide phases should have very similar particle sizes, the mixture should be very homogeneous and the described preparation via the adhesive tape method was assumed as suitable.

The absorption spectrum was acquired by measuring with and without the sample and an in-house data processing of the 2D-images [4]. The measurements were performed at room temperature with a measurement time of 10 h for each sample/reference prepared on adhesive tape and 2.5 h for each sample/reference prepared as wax pellet in order to achieve best possible signal-to noise ratio. The energy axis of the spectra was calibrated by comparing an α-Fe foil or powder reference measurement (performed before every sample measurement) with an α-Fe foil synchrotron radiation measurement. Deviation in the energy axis could still exist but an absolute energy axis is not required in this work since the deviation will be the same for sample and reference spectra. Spectra normalization was performed using *ATHENA* of the *Demeter* software package [51] and, in case of the iron ore sample, additionally the software package *Larch* [52]. The linear combination fitting (LCF) for the quantitative evaluations was performed in form of different approaches using *ATHENA* [51], the software package *Larch* [52] or an in-house algorithm [4]. These packages were used based on the fact that *ATHENA*, due to its user-friendly graphical interface, is frequently used for XAFS analysis. However, it is no longer supported. In contrast, *Larch* is continuously developed and can also be used as a Python package. The in-house algorithm first applied in [4] was utilized due to its superior transparency and flexibility compared to the other two open-source software options. In all cases, the results obtained by LCF, which relate to the ratio *r*_i_ of the bound iron in each species, were converted to weight percentages of the species for comparability with the other two methods by formula (S1) (see S1 File). The details of the normalization and LCF procedures are presented as part of the results section.

### X-ray diffraction

For the X-ray diffraction measurements of the α-Fe_2_O_3_/Fe_3_O_4_ model mixtures (as well as measurements of the pure reference substances α-Fe_2_O_3_ and Fe_3_O_4_) a *Panalytical X’Pert PRO* diffractometer with a Bragg-Brentano setup was used. The diffractometer operates with a Cu anode and without a monochromator (Cu Kα radiation) at 40 kV and 30 mA. At room temperature the diffraction data were obtained over a measurement range of 10–120° 2*θ*. For all three model mixtures the measurement time was 92 min (for the measurements of the pure reference substances 30 min). Samples were applied flat on a cut-off Si wafer attached to the sample holder. The quantitative XRD evaluation was performed using the standardless method of Rietveld refinement [53], since it is one of the most frequently XRD quantification techniques [25]. The Rietveld refinement was done with *Fullprof* [53,54].

For the examinations of the Mexican iron ore a different X-ray diffractometer was used. It is well known from the literature that the application of standard configurated diffractometers, hence, the application of copper radiation (Cu Kα), in the analyses of iron containing samples yield to limited diffractogram quality. The reasons for this are a reduced diffracted beam intensity (X-ray absorption, respectively inelastic scattering) as well as an increased background (X-ray fluorescence) [55]. This can impede and distort the phase identification, differentiation and quantification especially in cases of iron oxides/ hydroxides with very similar crystal structures (Fe_3_O_4_, γ-Fe_2_O_3_) or, in general, more complex multi-phase mixtures of iron oxides/ hydroxides as it can occur for example in geological samples. To overcome these issues the use of monochromators (e.g., a graphite monochromator as it was used in the pre-characterizations of the sample system in this study, see below and SI) or, ideally, a better suitable radiation source is required [55]. Especially cobalt radiation (Co Kα) is an ideal and common choice for examinations of such iron containing samples [55]. Therefore, to get optimal results the X-ray diffraction measurement of the Mexican iron ore sample has been performed with the Benchtop XRD diffractometer *Bruker D2Phaser* with a Cobalt X-ray source and a SSD160 detector (active length = 12 mm). The measurements were performed at room temperature in the measurement range 10°- 90° 2*θ* with 0.014° step size and 4.8 s/step, resulting in a total measurement time of 8 h. During the measurement the sample was rotated with 10 rpm. The sample was filled in PMMA-holders (Ø 2.5 mm) using the top-loading technique. The analysis was carried out using a 1-mm fixed divergence slit, a 2.5° primary and a 4° secondary soller collimator, a fixed knife edge (3 mm above the sample surface), and an Fe K_β_ filter (2.5). Qualitative phase analysis was conducted using *DIFFRAC.EVA V5.2* (*Bruker*) and the *Crystallography Open Database* [56]. Quantitative estimations via Rietveld refinement [53] were carried out using two different evaluation programs. The reason for this was to consider both, a common commercial as well as a common non-commercial software, and to compare the results. Therefore, the *Bruker* software *Topas V6* as well as the open-source and platform-independent software *Profex 4.2.4/BGMN* were applied [57–59].

### Mössbauer spectroscopy

Mössbauer spectroscopy was performed at a *MIMOS II* type spectrometer set-up with a ^57^Co source (in rhodium matrix) and Si-PIN detector [39,60]. In contrast to the backscattering-operated *MIMOS II* applied for the Martian surface analyses, the set-up used in this study can be operated either in backscattering or transmission mode. For the analyses the ^57^Fe-γ-line *E* = 14.4 keV was used and α-iron (α-Fe foil) was applied for the velocity/ energy calibration before the samples were analyzed. The samples were prepared in Mössbauer plastic powder sample holders consisting of an outer and an inner part which fit smoothly but tightly into each other. Thus, small amounts of the sample powders were placed in the outer parts in that way that a thin and even powder layer was obtained. Afterwards, to cap and to fixate the sample, the inner part was placed into the sample containing outer part. This was performed by gently rotating the inner part during the insertion into the outer part. This combination of careful pushing and rotating yielded to relatively thin, even and fixed sample layers. After a visual check for the mandatory absence of holes or gaps in the powder layers, the finished processed sample holders were sealed with *Parafilm* around the edges to ensure the tight fit of the sample holder parts, the fixation of the sample layers as well as a further reduction of air contact of the samples. The prepared samples were measured in transmission mode at room temperature. For all samples the measured velocity range was *v* = –10 mm/s - 10 mm/s (acceleration, synchronized with a 512 velocity channels multichannel analyzer, by Mössbauer drive in triangle-mode). The measurement time varied between 10 h and 120 h depending on the sample. In detail the measurement times were: α-Fe_2_O_3_ reference 24 h, Fe_3_O_4_ reference 72 h, α-Fe_2_O_3_-Fe_3_O_4_ model mixture 30/70 24 h, α-Fe_2_O_3_-Fe_3_O_4_ model mixture 50/50 120 h, α-Fe_2_O_3_-Fe_3_O_4_ model mixture 70/30 72 h and Mexican iron ore 10 h. The obtained spectra were mainly evaluated in two steps. In a first step the raw spectra were folded (software: *Fold it!*, Johannes Gutenberg Universität Mainz, Germany). Usually, this folding process is basically a “mirroring” of a raw 1024 channel spectrum (which consists of two mirror-imaged spectra of 512 channels each) relative to a center line. Thus, the transmitted gamma radiation intensities recorded in the channels that are symmetrical to this line are simply added or interpolated to get the “folded” 512 channel raw spectrum [61]. However, as it is typical for *MIMOS II* type spectrometers [61], the spectrometer applied in this study just had 512 velocity channels available. Therefore, 512 channel mirror-imaged raw spectra were transferred into “folded” 256 channel raw spectra. In the second step the fits were done in the form of Lorentzian site analyses (Software: *Recoil*, Department of Physics, Ottawa, Canada). The Lorentzian multiplet analysis is one of the most common least-squares based (reduced chi-squared $\chi_{\nu }^{2}$) fitting approaches in Mössbauer spectroscopy to perform a full spectrum analysis and to consider several line shapes (singlets, doublets and also sextets). Basic principles of this approach are the assumption that a measured spectrum consists of accumulated different subspectra as well as the approximations of all line shapes observed in the spectrum in form of Lorentzian lines/ distributions (Cauchy distribution) [62,63]. For the subsequently quantitative evaluations a common approximating approach, described in [32], was used. In this approach the ratio *N*(A)/*N*(B) of the amount of iron atoms of two different types A and B is in a simple association only with the observed Mössbauer signal areas *A*(A) and *A*(B) as well as the Mössbauer fractions *f*(A) and *f*(B). This correlation is described by the formula (S4) (see S1 File). The obtained atom amount ratios were transferred into weight percentages, respectively mass ratios of the species. The details on the quantitative evaluations are presented as part of the results section.

### Sample system

As reference substances pure iron(III) oxide (α-Fe_2_O_3_) from *Honeywell* and iron(II, III) oxide (Fe_3_O_4_) from *Sigma Aldrich* were obtained [64,65]. Both were described as micro particle powders (α-Fe_2_O_3_: *d* < 5 µm, Fe_3_O_4_: *d* < 5 µm) with a purity of ≥ 99% in case of iron(III) oxide and 95% in case of the iron(II, III) oxide. The reference substance samples analyzed in scope of this study were collected with plastic spatulas from freshly opened containers and were stored in snap-on cap bottles sealed with *Parafilm* beforehand and in between the examinations. The primarily analyzed sample of this study was an oxidic iron ore from Mexico and was purchased from *Mineraliengrosshandel Hausen GmbH* (Telfs, Austria). It must be emphasized that this iron ore has been available and obtained under the inaccurate name “Mexican magnetite” and, thus, has been expected to be a (relatively) pure mineral. However, the examinations performed in this study (pre-characterizations as well as the measurements in focus, quantitative XAFS, XRD and Mössbauer spectroscopy) clearly showed that the sample was not a (pure) mineral, but rather a rock because it consisted of more than just magnetite. Hence, in the following, the sample will be referred to by the more accurate name “Mexican iron ore”. The sample was delivered as grey massive polycrystalline pieces, which had sizes of a few centimeters (see SI Fig S1 in S1 File). Hence, some of the sample pieces were crushed with a hammer (wrapped with *Parafilm* to minimize contaminations) and then milled for 5 min at 650 rpm by the use of a ball mill *Pulverisette 6* (*Fritsch*, Idar-Oberstein, Germany) with zirconium dioxide (zirconium(IV) oxide, ZrO_2_) grinding tools. The resulting grey-red powder was utilized in all described further analyses and experiments. The observed powder color was a first indication that the sample was actually a mixture of magnetite and hematite (see microscopic examination, Fig S2c in S1 File). Beforehand and in between the numerous measurements of this study the sample powder was stored in HDPE containers or snap-on cap bottles which were sealed with *Parafilm* and additionally packed in sample bags to minimize the chance of speciation changes, e.g., by further oxidation due to profound air contact.

The purchased pure substances were characterized in detail before they were used as reference substances or standards in the XAFS spectrometer. To check the particle sizes light microscopy was done, micro X-ray fluorescence analysis (µ-XRF) and a microwave assisted digestion (in aqua regia) followed by inductively coupled plasma optical emission spectroscopy (ICP-OES) were used for elemental analysis and qualitative X-ray powder diffraction (XRD) for speciation. Additional to the initial classical macroscopic mineral determination methods to raw sample pieces (Table S1 in S1 File), the milled iron ore sample was also characterized in the same way as the reference substances. In this section, the characterization results are just presented in the form of a short overview (for more details see [66] and S1 File ).

The observed particle sizes (Fig S2a–c in S1 File) were in case of the iron(III) oxide and iron(II, III) oxide powders in XAFS suitable magnitudes (*d* < *µ*^–1^ [45]) since, consistent with the manufacturer information, both samples consisted of particles smaller than 5 µm (see boundary *µ*^–1^ particle size at *E* = 7.14756 keV based on NIST [67]: *d*(Fe_2_O_3_) ≈ 6.8 µm, *d*(Fe_3_O_4_) ≈ 6.6 µm). In contrast, the prepared iron ore powder mainly contained particles in the range of 10–20 µm. Assuming a composition of iron oxides it is evident that the particle size was slightly above the ideally XAFS suitable magnitude. Thus, slightly limiting effects on the XAFS spectra quality (e.g., suppression of white line features) could not be excluded and had to be considered.

The ICP-OES analyses were done regarding the element contents of iron, aluminum and chromium as well as cobalt and nickel. Based on µ-XRF results (Table S2 in S1 File), manganese and, in case of the iron ore sample, calcium were also considered. Additionally, the ore sample was semi-quantitatively analyzed with regard to possible contents of rare earth elements (REE), because some REEs can interfere with the Fe K-edge in XAFS [45]. The determined iron as well as iron oxide contents of the iron(III) oxide (*ω*_rel_(Fe) = 68.16% ± 1.34%) and iron(II, III) oxide micro particles (*ω*_rel_(Fe) = 68.25% ± 1.37%) were in good accordance to the manufacturer information (Table S3 in S1 File). It has to be pointed out that the iron(II, III) oxide micro particles also contained, inter alia (Table S4 in S1 File), relatively high contents of aluminum (*ω*_rel_(Al) = 2515.7 mg/kg ± 68.5 mg/kg), which could somewhat limit the suitability of these as a reference substance, for instance due to matrix effects, which can have numerous effects on the analytical signal in each method [68]. Nevertheless, in general the purity of both substances was sufficient for the use as a laboratory XAFS reference substance. The measured iron content of the Mexican iron ore (*ω*_rel_(Fe) = 63.05% ± 1.45%) was lower than literature values of pure magnetite or hematite (Table S3 in S1 File) and the contents of aluminum, calcium as well as silicon were relatively high (Table S2 and S4 in S1 File). This observation is explainable by impurities of the magnetite and hematite as well as common accompanying minerals. REEs were not detected.

The qualitative XRD (Fig S3a–c in S1 File) analyses also demonstrated the identity and purity of the reference substances. Especially the fact, that the XRD pattern of the Fe_3_O_4_ micro particles only showed the desired Fe_3_O_4_ reflections (Fig S3b in S1 File), confirmed that these particles were also sufficiently pure for the use as a laboratory XAFS reference substance in course of this work. The XRD of the Mexican iron ore finally verified the assumption of a mineral mixture mainly consisting of magnetite and hematite. This paragenesis is explainable by the fact that magnetite can be transferred to iron(III) species (e.g., hematite, maghemite or oxide-hydroxides like goethite and others [limonite]) due to secondary mineral formation processes based on weathering, metamorphism and especially pseudomorph formation (martitization) [69–72].

Furthermore, alpha-iron(III) oxide/ iron(II, III) oxide model mixtures with precisely determined (weigh-in) mass ratios *ω*_rel_(α-Fe_2_O_3_)/*ω*_rel_(Fe_3_O_4_) were prepared and also quantitatively analyzed by XAFS, XRD and Mössbauer spectroscopy, beforehand the iron ore examinations. The goal was to get more information on the general accuracy of the Lab-XAFS and the other two methods by considering this, in comparison to the natural iron ore, similar but simpler and more precisely known sample system. Thus, it was appropriate to count the actually achieved weigh-in ratios as true values and, based on ISO 5725–1 [49], the accuracy as closeness (or distance) to these. Hence, the accuracy is in relation to the deviation of the measured values and the true values. As source materials the same iron(III) oxide and iron(II, III) oxide which had already been pre-characterized and used as pure reference materials in this study (see above), were applied. The mixtures were prepared by weigh-in (precision balance *Adventurer Pro*, *σ* = 0.0001 g, *Ohaus*, Greifensee, Switzerland) the pure reference substance powders and mixing them in an agate mortar. In total three different α-Fe_2_O_3_-Fe_3_O_4_ mixtures with the nominal mass ratios *ω*_rel_(α-Fe_2_O_3_)/*ω*_rel_(Fe_3_O_4_) 30/70, 50/50 and 70/30 were produced (see Table 1 and S5 in in S1 File for the actually achieved ratios). The mixtures were stored in sealed snap-on cap bottles. To compensate possible segregations occurred during the storage and transport, the mixtures were always re-homogenized beforehand the measurement preparations.

**Table 1:** Quantitative results for the α-Fe_2_O_3_/Fe_3_O_4_ model mixtures of the lab-based methods XAFS, XRD and Mössbauer. The results are presented as mass ratios *ω*_rel_(α-Fe_2_O_3_)/*ω*_rel_(Fe_3_O_4_).

Nominal ratio *ω*_rel_(α-Fe_2_O_3_)/ *ω*_rel_(Fe_3_O_4_)	Achieved weigh-in ratio *ω*_rel_(α-Fe_2_O_3_)/ *ω*_rel_(Fe_3_O_4_)	*ω*_rel_(α-Fe_2_O_3_) measured in %	*ω*_rel_(Fe_3_O_4_) measured in %
**30/70**	31.8/68.2	XAFS tape: 25.4 ± 0.6	XAFS tape: 74.7 ± 0.6
		XAFS pellet: 33.0 ± 2.7	XAFS pellet: 67.0 ±** **3.5
		XRD: 32.0 ± 1.2	XRD: 68.0 ± 1.5
		Mössbauer: 38.6 ± 1.5	Mössbauer: 61.4 ± 3.4
**50/50**	50.6/49.4	XAFS tape: 44.0 ± 0.6	XAFS tape: 56.0 ± 0.6
		XAFS pellet: 53.0 ± 1.6	XAFS pellet: 47.0 ± 2.8
		XRD: 50.8 ± 1.8	XRD: 49.2 ± 1.8
		Mössbauer: 54.7 ± 1.0	Mössbauer: 45.3 ± 1.7
**70/30**	70.5/29.5	XAFS tape: 65.6 ± 0.6	XAFS tape: 34.4 ± 0.6
		XAFS pellet: 87.0 ± 2.9	XAFS pellet: 13.0 ± 2.9
		XRD: 67.9 ± 2.1	XRD: 32.1 ± 1.8
		Mössbauer: 75.0 ± 0.8	Mössbauer: 25.0 ± 1.1

The results represent the mass ratio of α-Fe_2_O_3_ and Fe_3_O_4_. The given percentage is the value of the whole species and not only the iron. The uncertainty is not comparable, for more information see SI. Percentages may not sum up to exactly 100% due to rounding. The results XAFS tape, XRD and Mössbauer have been obtained by single determination (regarding sample preparation) whereas the XAFS pellet results 30/70 and 50/50 are averaged values of two sample pellets each (two replicate determinations) (in case of 70/30 just one of the two prepared pellets was utilizable due to a systematic error during the preparation).

## Results

In the following section the results of the measurements obtained by each of the three different analytical techniques will be described. The presentation of the lab-XAFS, XRD and Mössbauer results will follow the order pure reference substances, model mixtures and, finally, Mexican iron ore respectively.

Furthermore, it must be emphasized that the measurements of the Mexican iron ore sample with all three different techniques were performed at about the same time. This means that the storage times of the sample were very similar at the times of measurements. Hence, differences in the results tracing back to disparate sample aging due to dissimilar storage conditions or times could largely be excluded. Notable sample aging effects, e.g., a further oxidation of the Fe_3_O_4_ portion of the iron ore, during the measurements could be precluded at least for the XAFS and Mössbauer spectroscopic measurements. In both cases the samples were protected from air contact due to the type of sample preparation (XAFS: sample powder encapsulated in adhesive tape layers, Mössbauer: sample powder in plastic powder sample holders sealed with *Parafilm*).

Finally, it needs to be highlighted that, analogous to the examinations and results of the model mixtures, the presented quantitative results for the Mexican iron ore will be focused primarily on the mass ratios *ω*_rel_(α-Fe_2_O_3_)/*ω*_rel_(Fe_3_O_4_). These two iron species were expected on the basis of the pre-characterization results. It was the intention to perform the examinations with the three different species analysis techniques independently of each other and, thus, each just based on these pre-characterization results and, finally, to compare the individual results. In fact, only α-Fe_2_O_3_ and Fe_3_O_4_ were identified in the obtained XAFS spectrum and XRD pattern. However, in scope of the quantitative examinations the Mössbauer spectrum revealed the probable presence of certain amounts of a third iron-containing phase (γ-Fe_2_O_3_, respectively Fe_3–x_O_4_). Attempts of an additional quantitative consideration of this third phase will be presented/ discussed in the discussion section in more detail.

### X-ray absorption fine structure spectroscopy

#### XAFS – Pure reference substances.

**Fig 1:**
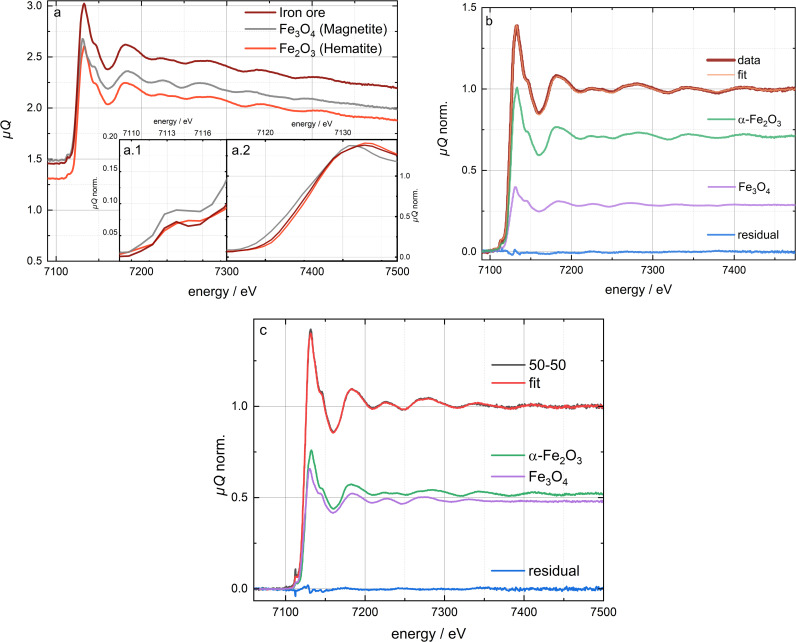
XAFS-spectra of the reference substances **α-Fe**_**2**_**O**_**3**_ **and Fe**_**3**_**O**_**4**_**, the** **α-Fe**_**2**_**O**_**3**_**/Fe**_**3**_**O**_**4**_ **model mixtures and the Mexican iron ore**. a unnormalized XAFS-spectra of the sample and the references, with insets of the normalized edge region (a.2) and the pre-peak area (a.1). The complete normalized spectra can be seen in Fig S4a,b (SI). **b** LCF results performed by the *ATHENA* software [51] on the normalized spectra with the flatten algorithm of the Mexican iron ore. Fit and data show only minor deviations, indicated by the residual. The LCF results with the *Larch* software [52] and the in-house algorithm on the unflattened and flattened normalized spectra can be seen in Fig S8 (S1 File ). The LCF results are displayed in Table S7 (S1 File ). **c** LCF results of the 50/50 α-Fe_2_O_3_/Fe_3_O_4_ model mixture (adhesive tape preparation). For each sample/reference the measurements were performed at room temperature with a measurement time of 10 h.

The obtained XAFS spectra of the pure substances alpha-iron(III) oxide and iron(II, III) oxide micro particles can be seen in Fig 1a. A qualitative analysis with focus on the XANES area including the pre-edge of these pure potential reference substances was performed. This was essential to verify the quality of the obtained spectra regarding, e.g., the presence of all theoretically expected essential substance specific fine structures as well as if the spectral resolution is high enough to distinguish the different species, before these spectra were used in the quantitative analysis as references. As a result, the qualitative analysis revealed the expected characteristic fine structures of α-Fe_2_O_3_ and Fe_3_O_4_ and the differences between them, as it can be seen in Fig 1a and S5 in S1 File between 7110 eV and 7150 eV and Fig 1a between 7225 eV and 7350 eV (though the spectra of both compounds also show distinct similarities in general) due to different structures. All of these aspects were in correlation with the theory of influences of the chemical and structural environment of an absorber atom on the XAFS, as it is described in literature in general [45–48] as well as with regard to iron compounds and the Fe K-edge in particular [73–76]. Thus, as observable in Fig 1a and S5 in S1 File, the edge and the pre-edge of Fe_3_O_4_ are shifted to slightly lower energies compared to α-Fe_2_O_3_. This can be explained by the fact that Fe_3_O_4_ is a mixed-valence compound containing both Fe(II) and Fe(III) (in case of Fe(II) somewhat lower energy for the electron excitations is required) whereas α-Fe_2_O_3_ just contains Fe(III). Furthermore, the pre-edge of Fe_3_O_4_ is more intensive than the Fe_2_O_3_ pre-edge (Fig 1a inlet a.1 and Fig S5 in S1 File), which is in relation to the different crystal structures (Fe_3_O_4_: inverse spinel-type structure, α-Fe_2_O_3_: corundum-type structure) [43,71,77–79] and, therefore, to different coordination polyhedra. In case of Fe_3_O_4_ 2/3 of the iron cations are in an octahedral coordination (Fe(II) and Fe(III)), but the remaining 1/3 of the cations are in a tetrahedral coordination (Fe(III)), whereas in case of the α-Fe_2_O_3_ all Fe(III) cations are in octahedral sites.[43,71,77,79] Since a tetrahedral coordination enables the more intensive E1 (electric dipole) transitions in the 1s – 3d excitations in addition to the generally less probable, but in case of octahedral coordination solely present E2 (electric quadrupole) transitions in the 1s – 3d excitation, this more intensive pre-edge of Fe_3_O_4_ is explainable.

#### XAFS – model mixtures.

The normalized XAFS spectra including the LCF results of the three α-Fe_2_O_3_/Fe_3_O_4_ model mixtures (both prepared by the adhesive tape and the wax pellet method) are shown in Fig 1c (adhesive tape 50/50 mixture) as well as Fig S6 and S7 in S1 File in comparison to the spectra of the pure reference compounds. The LCF of the mixtures was only performed on flatten normalized spectra using *ATHENA* of the *Demeter* software package [51]. The fitting range extended from 10 eV before to 320 eV beyond the edge, similar to the LCF performed on the Mexican iron ore (see below).

The quantitative results from the fits are listed in Table 1 (for more detailed determined compositions see Table S6 in S1 File) in comparison to the results obtained by the other two methods. The quantitative results of the mixtures prepared on adhesive tape, which were measured together with the Mexican iron ore, deviate from the actual weigh-in ratio (the true value) between 4.9 and 6.6 p.p. (percentage points). Since the iron ore was prepared with the same method and the statistical uncertainty for the measurement of the spectra is comparable, it can be assumed, that the deviation for this analysis is below 7 p.p. accordingly (details see discussion section). Using the wax pellets, the results of the LCF deviated 1.2 p.p, 2.4 p.p and 16.5 p.p. for the target ratios of 30/70, 50/50 and 70/30 respectively. Noticeable is the obtained much larger bias and, thus, lower accuracy in case of the 70/30 mixture, which was caused by a systematic error in the sample preparation (see discussion section).

#### XAFS – iron ore.

The obtained Mexican iron ore spectrum is shown in comparison to the reference substance spectra in Fig 1a. In accordance to the pre-characterization results, the qualitative analysis of the ore sample revealed a composition of both iron oxides, as the spectrum clearly contained fine structures of both iron compounds and, e.g., edge and pre-edge positions between the two (see Fig 1a and S5 in S1 File).

For further analysis and quantification evaluation by linear combination fitting (LCF) the spectra where normalized using *ATHENA* of the *Demeter* software package [51] (see Fig S4a in S1 File) and the software package *Larch* [52] (see Fig S4b in S1 File). The differences in the normalized spectra result from the flatten algorithm in *ATHENA* which is set to default. While *ATHENA* can perform LCF on the flattened and unflattend normalized spectra, *Larch* can only perform LCF on unflattened XAFS-spectra. In general, normalization is carried out in the following steps: First, the pre-edge region is interpolated using a linear fit (pre-edge line), while the normalization range beyond the edge is interpolated using a polynomial fit (post-edge line). These fits can be manually adjusted by modifying the pre-edge and normalization ranges. The criteria for defining a suitable pre- and post-edge line are subjective. In case of the iron ore sample (and also the model mixtures) examined in this study, the post-edge line was chosen to pass through the middle of the data. A detailed documentation of this process is available in the *ATHENA* online manual [80]. The next step involves determining the normalization constant *µ*₀(*E*₀), analogous to the edge step, by extrapolating the pre- and post-edge lines to *E*₀ − their intersection point − which defines the edge step parameter. In conventional normalization (without the flattening algorithm), the extrapolated pre-edge line is subtracted from the spectrum, which is then divided by the edge step parameter. In contrast, when using the flattening algorithm, the difference in slope and curvature between the pre- and post-edge lines is subtracted from the data, aligning all oscillations to *µQ* or *y* = 1. This approach facilitates data comparison, as conventional normalization can cause spectra to diverge at higher energies.

The normalization procedure is a crucial part for the linear combination fitting. Different normalized spectra can lead to different results. A broad energy range enables a better normalization with a more reliable result. But even if a single person took great care to perform a consistent normalization of sample and reference standards the results could be off relative to each other by 10% [81]. Due to the meridional limitation of the HAPG-crystal of 5 cm the recorded energy range extends only from about 50 eV before the edge to 400 eV behind the edge. This impedes consistent normalization even further, hence, as described above, the points for the post edge line need to be manually adjusted until the line passes through the middle of the oscillations.

The results of the LCF can be seen in Fig 1b and S8 in S1 File. It shows the fit and the data as well as the residual of the Mexican iron ore for four different LCF. LCF has been performed with *ATHENA* (flattened),[51] *Larch* (unflattened) [52], and with the in house algorithm [4] on the flattened and unflattened normalized spectra. The four different LCF were performed in the energy range between 7095 eV to 7475 eV. The LCF results and thus the resulting determined compositions are listed in detail in the SI (Table S7 in S1 File). It is clearly visible, that all LCF approximate the data well and have only minor oscillations in the residual, especially in the white line. Table 2 shows the averaged result of the four differently performed LCF. Thus, an overall averaged sample mass ratio composition of about 72% ± 3% hematite (α-Fe_2_O_3_) and 28% ± 3% magnetite (Fe_3_O_4_) was determined.

**Table 2:** Quantitative results for the Mexican iron ore of the lab-based methods XAFS, XRD and Mössbauer.

Method	α-Fe_2_O_3_ in wt. %	Fe_3_O_4_ in wt. %	γ-Fe_2_O_3_ in *wt.* %
**XAFS-2**	72 ± 3	28 ± 3	
**XAFS-3**	56 ± 3	17 ± 5	27 ± 3
**XRD**	74 ± 1	27 ± 1	
**Mössbauer-2**	57 ± 1	43 ± 2	
**Mössbauer-3**	49	38	13

The results represent the mass ratio of *ω*_rel_(α-Fe_2_O_3_)/*ω*_rel_(Fe_3_O_4_) (XAFS-2, XRD and Mössbauer-2). Additionally the mass ratio *ω*_rel_(α-Fe_2_O_3_)/*ω*_rel_(Fe_3_O_4_)/*ω*_rel_(γ-Fe_2_O_3_), thus, an alternative result also considering a third iron oxide species which presence was hinted by the Mössbauer evaluation is shown (XAFS-3, Mössbauer-3: for the Mössbauer-3 results no errors are presented since the outcomes mostly base on a calculation/ approximation, see discussion). The XAFS-2 result is the average of the four different LCF methods with the uncertainty by the span of the values. The XRD result is the average of the Rietveld refinement done with the *Topas* and the *Profex* software. The given percentage is the value of the whole species and not only the iron. The uncertainty is not comparable, for more information see SI. Percentages may not sum up to exactly 100% due to rounding.

To compare the actual quality of the LCF of the different fitting approaches by open-source and in-house software $\chi^{2}$ and reduced $\chi_{\nu }^{2}$ have been calculated by the equation (S2 in S1 File) in the SI. By considering the value of the $\chi_{\nu }^{2}$ of all fits (Table S7 in S1 File) both in-house LCFs for flattened and unflattened normalized spectra achieved the best fit to approximate the data, even though all applied LCFs overall showed good results.

### X-ray diffraction

#### XRD – pure reference substances.

Since XRD examinations of the pure reference substances alpha-iron(III) oxide and iron(II, III) oxide have already been part of the pre-characterizations the results of these re-measurements are not presented in detail. In total, the measured diffractograms were well explained by the expected iron oxide species/ phase (α-Fe_2_O_3_ for the iron(III) oxide micro particle powder and Fe_3_O_4_ for the iron(II, III) oxide micro particle powder). Hence, the obtained results were completely consistent with the pre-characterization XRD outcomes. The raw data of these re-measurements can be found in the data repository entry associated with this study (see section Data availability).

#### XRD – model mixtures.

The measured diffraction patterns of the three α-Fe_2_O_3_/Fe_3_O_4_ model mixtures including the results of the Rietveld refinement for the quantitative evaluation of the two iron species are presented in Fig S11-13 (S1 File). The detailed quantitative results as well as the statistical parameters (R_wp_, R_exp_ etc.) for the Rietveld refinement are listed in the Table S9 (S1 File). Nevertheless, the end results are also presented in Table 1 (and Table S8 in S1 File) in comparison to the outcomes of XAFS and Mössbauer spectroscopy.

In total, α-Fe_2_O_3_/Fe_3_O_4_ mass ratios were determined that were of the noticeably small bias (high accuracy) 0.2 p.p. for the 30/70 and 50/50 as well as 2.6 p.p. for the 70/30 target ratios. However, it must also be highlighted, that the deviation obtained in case of the α-Fe_2_O_3_/Fe_3_O_4_ 70/30 model mixture (2.6 p.p.) was distinctively higher compared to the other two mixtures, although still very close to the target ratio especially when also considering the determined uncertainty of examined result (±3 for *ω*_rel_(α-Fe_2_O_3_) and ±2 for *ω*_rel_(α-Fe_3_O_4_)).

#### XRD – iron ore.

**Fig 2:**
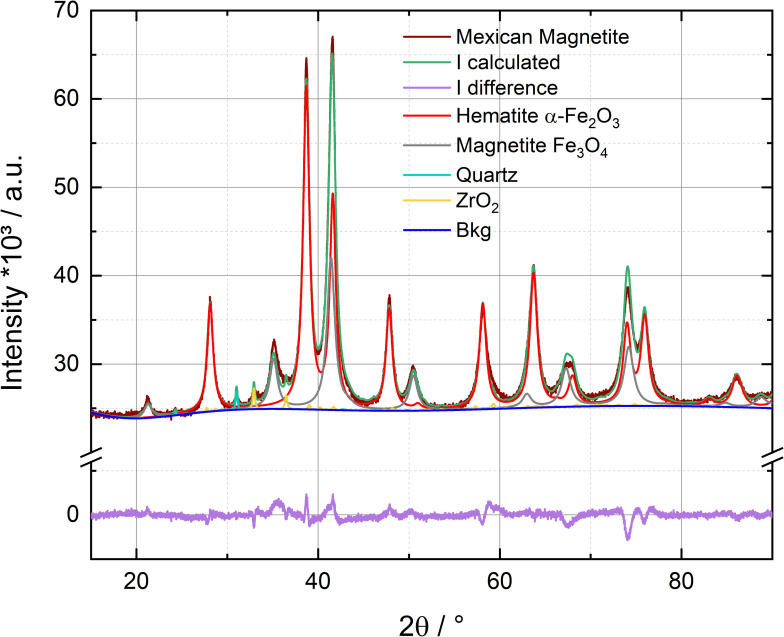
XRD pattern of the Mexican iron ore with quantitative evaluation by Rietveld refinement with *Profex 4.2.4/BGMN.* The results of the Rietveld refinement with *Topas V6* (*Bruker*) can be seen in SI, Fig S14a in S1 File. The quantitative results are listed in Table S10 (in S1 File). The measurement time was 8 h. The measurements were performed at room temperature.

The obtained XRD pattern of the Mexican iron ore with the results of the Rietveld refinement by *Profex 4.2.4/BGMN* for the quantitative determination of the iron species can be seen in Fig 2 (for the refinement with *Topas V6* see Fig S14 in S1 File). The quantitative results obtained with both software packages (*Topas V6* and *Profex 4.2.4*) are listed in Table S10 (in S1 File). Applying both programs, the identified crystalline phases were magnetite (Fe_3_O_4_), hematite (α-Fe_2_O_3_), (low) quartz (α-SiO_2_) and monoclinic zirconium(IV) oxide (ZrO_2_). While the α-SiO_2_ is of natural origin the ZrO_2_ was introduced into the sample through the grinding/milling process using a ZrO_2_ grinding tool (see above).

The statistical parameters R_wp_, R_exp_ and GOF (Goodness of fit also known as χ) for the Rietveld refinement are listed for both software in the Table S10 (in S1 File). The GOF is 2.53 for the *Profex* software and 2.73 for *Topas*, suggesting that the used model is reasonable, even though it is no guarantee that the model is correct.[82] Since there is no rule-of-thumb to tell if the model/fit can describe the sample accurate other aspects like the chemical model or the graphical results of data and fit should be considered [82]. The uncertainty of the mineral composition in Table S10 in S1 File is given by 3x e.s.d. according to ASTM E117-13 [83]. It has to be pointed out that this is only a mathematical estimation of the uncertainty. The calculated and the observed pattern have minor deviation, which means that missing a species in the used model cannot be excluded. However, all observed peaks can be explained by the chosen species. If there are any additional phases present, these must be very similar (very similar/ identical peak quantities, positions, and intensities) to those phases which have already been found (e.g., similarity γ-Fe_2_O_3_ and Fe_3_O_4_). Additionally, the purities of the identified iron phases must also be taken into account. Since noticeable amounts of aluminum, which could be accommodated in the two iron oxides (isomorphic substitution: Al(III) can Fe(III), see details Mössbauer results) of the iron ore sample, have been detected in scope of the sample pre-characterizations, slight effects on diffraction peak intensities and positions can also contribute to the deviation. However, in total, the XRD results suggest that the used chemical model should be sufficient for the purpose of this work*.*

Focusing on the XRD quantification of the Fe-phases, a sample composition of 73% ± 1% hematite (α-Fe_2_O_3_) and 27% ± 1% magnetite (Fe_3_O_4_) for *Topas* and 74% ± 1% hematite and 27% ± 1% magnetite for *Profex* respectively was determined. Because the deviation between the two obtained results is small and both applied fits are of sufficient quality, it is appropriate to use the averaged result of about 74% ± 1% hematite and 27% ± 1% magnetite (Table 2) in addition to the individual *Topas*- and *Profex*-based values for the further comparison. Percentages may not sum up to exactly 100% due to rounding.

### Mössbauer spectroscopy

#### Mössbauer spectroscopy – pure reference substances.

**Fig 3:**
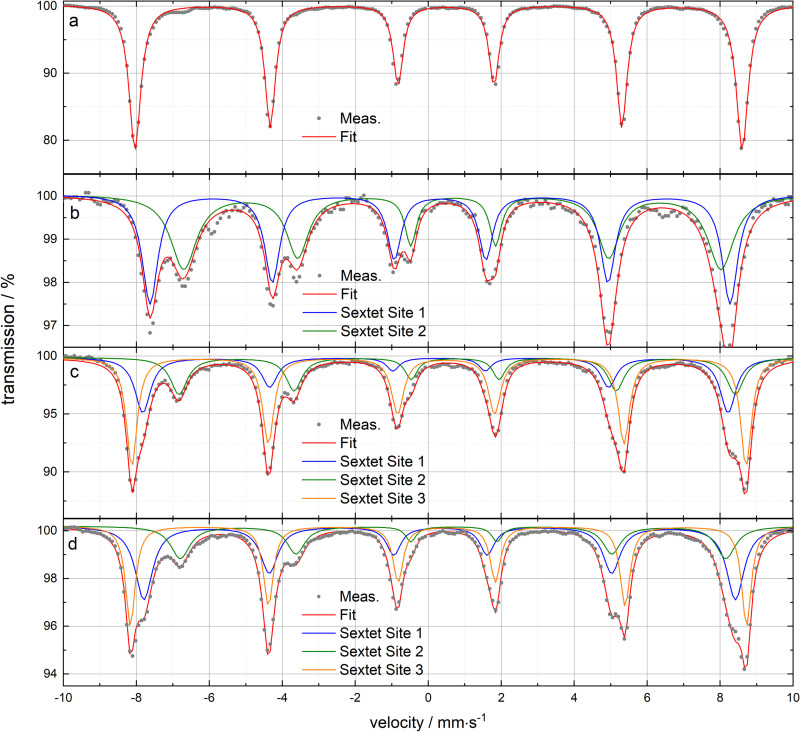
Mössbauer spectra of the reference substances, the model mixtures and the Mexican iron ore sample. a α-Fe_2_O_3_ micro particles ($\chi_{\nu }^{2}=7.748$), b Fe_3_O_4_ micro particles (sextet site 1: A-site, sextet site 2: B-site) ($\chi_{\nu }^{2}=1.110$), c Mexican iron ore ($\chi_{\nu }^{2}=4.158$). d 50/50 α-Fe_2_O_3_/Fe_3_O_4_ model mixture ($\chi_{\nu }^{2}=2.692$) (see SI Fig. S15 in S1 File for 30/70 and 70/30 ratio). The total spectra fits are marked in red and the individual sextets in blue, green and orange. The measurement time was 24 h for α-Fe_2_O_3_, 72 h for Fe_3_O_4_, 10 h for the Mexican iron ore and 120 h for the 50/50 model mixture. The measurements were performed at room temperature.

Fig 3a,b show the resulting spectra of the reference substances alpha-iron(III) oxide and iron(II, III) oxide micro particles. The determined hyperfine parameters are listed in detail in Table S11 and S12 (S1 File) in comparison to literature.

The obtained spectra and hyperfine parameters of the two reference iron oxides were in quite good accordance to the chosen representative literature values considering the actual possible ranges in each hyperfine parameter. These ranges are caused by the dependency of the exact values on several sample parameters (e.g., crystallinity, particle sizes, purity) as it is described widely, for example in [31,32,84–87]. In case of the iron(III) oxide micro particle powder (Fig 3a) a single sextet was found which had hyperfine parameters in the typical range of octahedral coordinated Fe(III) in high-spin state [31,32]. In the iron(II, III) oxide micro particle´s case (Fig 3b) the two different sextets expected for the inverse spinel-type structure (Fe_3_O_4_: Fe(III)[T]Fe(II)[O]Fe(III)[O] O_4_, Fe(III)AFe(II, III)_2_B O_4_, resp.) were observed. The one for the tetrahedral high-spin Fe(III) (A-site) [32,88] and the second with a slightly broadened linewidth for high-spin Fe(II) and Fe(III) in the octahedral sites (B-site) (at room temperature Fe(II) and Fe(III) in these sites cannot be easily distinguished by Mössbauer spectroscopy because of a fast electron transfer between these iron cations) [32,43,88]. However, the expected ideal sextet area ratio *A*(B-site)/*A*(A-site) of 2:1 or, more practical (considering the A- and B-site´s different Mössbauer-Lamb factors/ Mössbauer fractions *f*), 1.8:1 [32,43] was not obtained. This is clearly visible at the obtained reversed absorption maxima ratios of the outer A-site sextet and the inner B-site sextet (Fig 3b, Table S11 in S1 File). In detail an area ratio of about 1:1 (≠ 1.8:1) was determined from the results (SI, Table S11 in S1 File). But as it is described in [32] this area ratio often differs in practical measurements from the ideal amount due to sample deviations from an ideal Fe_3_O_4_, or in case of mineral or rock samples deviations from an ideal magnetite. In general, these divergences can be caused by two effects.

Mainly, the sample deviations can be caused by partial Fe(II) oxidation (simplified a partial transition into γ-Fe_2_O_3_, in detail the formation of an oxidized, non-stoichiometric magnetite phase Fe_3–x_O_4_) [32]. Therefore, the absorption of the B-site decreases and a third sextet, which is very close to and in some cases difficult to distinguish from the pure Fe_3_O_4_ A-site sextet, appears. This overlay can cause an apparent increase of the A-site intensity and area. Altogether one will get a spectrum still consisting of two sextets (Fe(III) and Fe(II, III)), but with areas deviating from pure Fe_3_O_4_. Similar observations can also be found in [89], where a successful distinction between the A-site and the mentioned third sextet is used to quantify the amount of the γ-Fe_2_O_3_ phase in Fe_3_O_4_ nano particles. Overall the obtained lower ratio *A*(B-site)/*A*(A-site) could indicate that the iron(II, III) oxide micro particles contained amounts of oxidized phases. Even though it was paid attention to prevent the Fe_3_O_4_ sample from significant oxidation (sample taken from a freshly opened container, storage in sealed bottles etc.) during this study, the presence of such an oxidized phase cannot completely be excluded. Firstly, there was still a certain air contact because the sample storage and also the preparation were not performed under inert gas atmosphere. To perfect the oxidation prevention procedures, operations under nitrogen or argon atmosphere would be required. Secondly, oxidation processes could have also already occurred during the production and filling processes by the manufacturer.

However, the sextet area deviations can also be caused by contents of aluminum and/ or titanium (or others). Al(III) or Ti(IV) can partially substitute the iron(III) due to similar ionic radii and charges, thus, similar charge densities (isomorphic substitution) and form aluminomagnetites (Fe_3–x_Al_x_O_4_) or titanomagnetites (solid solution system Fe_3_O_4_/Fe_2_TiO_4_, thus, Fe_3–x_Ti_x_O_4_) which is also reflected in altered sextet area ratios [32,71,90]. Considering the pre-characterizations of the Fe_3_O_4_ micro particles, which revealed a high aluminum content of about 2500 mg/kg (0.25 wt.%), it can be assumed with confidence that the observed sextet area ratio deviation additionally traces back to this second effect.

#### Mössbauer spectroscopy – model mixtures.

The α-Fe_2_O_3_/Fe_3_O_4_ model mixtures Mössbauer spectra are shown in Fig 3d (50/50) and Fig S15a-c in S1 File. The associated detailed hyperfine parameters are presented in Table S11 in S1 File. As expected, the mixtures spectra consisted of the α-Fe_2_O_3_ sextet as well as the two Fe_3_O_4_ sextets, hence, in total three sextets.

For the quantitative evaluation to determine the mass ratios of α-Fe_2_O_3_/Fe_3_O_4_ a slightly adjusted version of equation (S4 in S1 File) in form of formula (S5a in S1 File) had to be applied. In this approach, the first quantification/ calculation step is just focused on the B-site sextet of the Fe_3_O_4_ (instead of considering both, the area of A- and B-site) and, thus, just on *N*(Fe B-site Fe_3_O_4_)/*N*(Fe α-Fe_2_O_3_). This was required due to two aspects. Firstly, in case of the iron ore examinations the same evaluation procedure was performed because interferences affected the A-site (see below). Thus, a comparability of the model mixture quantitative evaluation procedure and the one for the iron ore was ensured. Secondly, also in case of the pure Fe_3_O_4_ reference an affected A-site was observed in the qualitative Mössbauer analysis (see above). Therefore, the application of the adjusted quantification procedure met two advantages at the same time.

The required Mössbauer fractions *f* were obtained by averaging literature values because a detailed experimental determination of them was not accessible in scope of this study. In further steps, the calculated atom amount ratio was converted to amounts of the substances Fe_3_O_4_ and α-Fe_2_O_3_ and, finally, to the required mass fractions and ratios. The detailed calculation process of the quantification is presented in Table S13 (S1 File).

The final results are presented in Table 1 in comparison to the outcomes of the other methods as well as in more detail in Table S13 (S1 File). As it is apparent the results deviated 6.8 p.p., 4.1 p.p. and 4.5 p.p. for the target ratios of 30/70, 50/50 and 70/30 respectively.

#### Mössbauer spectroscopy – iron ore.

Fig 3c presents the obtained Mexican iron ore spectrum. The determined hyperfine parameters are listed in detail in Table S12 (S1 File) as well as [66] in comparison to the literature. The spectrum of the sample was adapted the best way through a fit considering three different sextets. The comparison of the associated hyperfine parameters to the determined reference substance parameters and the literature values (S1 File, Table S11 and S12) demonstrated that sextet 1 and 2 are best assigned to the A- and B-sites of Fe_3_O_4_ whereas sextet 3 fits to the shape of α-Fe_2_O_3_. Although it must be pointed out that the obtained magnetite hyperfine parameters are not in a direct match with the reference substance parameters or the chosen literature values (due to the parameter ranges), the scale of the values fitted sufficiently enough to do the assignment. Thus, in line with the pre-characterizations and XAFS, the qualitative evaluation of the Mössbauer spectra also showed the composition of Fe_3_O_4_ and α-Fe_2_O_3_, hence magnetite and hematite. However, a closer look at the area ratio of the two sextets belonging to Fe_3_O_4_ provided a very similar result to the reference Fe_3_O_4_ micro particles. An area ratio *A*(B-site)/*A*(A-site) of about 1 was obtained. Considering the fact that the iron ore sample is a mixture of magnetite and hematite because parts of a pure raw magnetite were transferred into hematite due to oxidative secondary mineral formation processes, it is plausible that also the residual magnetite in the mineral mixture is actually in part an oxidized magnetite phase (γ-Fe_2_O_3_ containing magnetite, respectively Fe_3–x_O_4_) as they are described in [32]. Furthermore, under certain conditions the geological transformation from magnetite into hematite can occur with maghemite (γ-Fe_2_O_3_) as an intermediate stage [70]. In total, in case of the iron ore sample the partial Fe(II) oxidation of magnetite can be assumed as the primary contribution yielding to the sextet area deviation, although additional effects due to Al(III) may also be present. However, even though a considerable aluminum content of about 1900 mg/kg (0.19 wt.%) was found in the ICP-OES pre-characterizations (see S1 File Table S4), a conclusion that all of the aluminum is just contained in the magnetite part of the ore is not feasible on the basis of the results of this study. Al(III) can basically also substitute Fe(III) in the hematite (solid solution system hematite/corundum, thus, α-Fe_2_O_3_/α-Al_2_O_3_) [71,91,92] and Si(IV) in the silicates [93,94] of the ore sample. However, since in the total digestion prior to the ICP-OES analyses major parts of the quartz (SiO_2_) contents remained in form of very small amounts of insoluble residues (see SI), it could be assumed that the aluminum content determined by ICP-OES mainly corresponds to Al(III) originated from the digested/ dissolved iron oxides. Nevertheless, a further differentiation between Al(III) in the hematite and Al(III) in the magnetite is not possible based on the results of this study. Thus, in total, the qualitative Mössbauer spectroscopic analysis suggests the presence of (small) amounts of an additional third iron oxide phase (α-Fe_2_O_3_, Fe_3_O_4_ and γ-Fe_2_O_3_).

These results were of crucial importance for the quantitative evaluation to determine the mass ratio of the mineral sample main components. The ore sample analyzed in this study contained at least three different types of iron atoms, two of them belonging to magnetite and one of them to hematite. As this investigation has shown the sample even contained a certain amount of a fourth type of iron atoms, but the content of this oxidized phase was not determined in the main quantification attempts. Nevertheless, the presence of this fourth iron atom type had to be considered in the selection of the individual Mössbauer sextets to execute the determination of the magnetite hematite weight ratio. Due to the mentioned overlay and the increased intensity of the magnetite A-site signal, only the B-site sextet was used for the first quantification/ calculation step. So, the fundamental of the quantification in this work was formula (S5b) (see S1 File ) which is an adjusted version of formula (S4 in S1 File) and is focused on *N*(Fe B-site magnetite)/*N*(Fe hematite).

Analogous to the examinations of the model mixtures, the Mössbauer fractions *f* were used by averaging literature values since a detailed experimental determination of them was not accessible during the time of this study. In further steps, the calculated atom amount ratio was converted to amounts of the substances Fe_3_O_4_ and α-Fe_2_O_3_ and, finally, to the required mass fractions and ratios. The detailed calculation process of the quantification is presented in Table S14 (S1 File). As a result, a mass ratio of about to 57% ± 1% hematite (α-Fe_2_O_3_) to 43% ± 2% magnetite (Fe_3_O_4_) was obtained (Table 2 and S14 in S1 File).

## Discussion & perspective

Tables 1 and 2 present the obtained quantitative results for the α-Fe_2_O_3_/Fe_3_O_4_ model mixtures and the Mexican iron ore sample of all three applied laboratory-based species analysis techniques and show the averaged rounded values (for the individual results see Table S6 - S10, S13 and S14 in S1 File). These results will now be discussed directly, but even further, each method’s capabilities and restrictions will also be covered.

### Discussion of quantitative results

For the analysis of the α-Fe_2_O_3_/Fe_3_O_4_ model mixtures (Table 1) the lab-XAFS LCF results of the adhesive tape prepared samples deviate 6.4 p.p., 6.6 p.p. and 4.9 p.p. for the target ratios of *ω*_rel_(α-Fe_2_O_3_)/ *ω*_rel_(Fe_3_O_4_) 30/70, 50/50 and 70/30 respectively. In comparison to the outcomes obtained by XRD, which are of distinctively high accuracy, thus, small bias (0.2 p.p., 0.2 p.p. and 2.6 p.p.) and show no significant differences to the true values (the achieved weigh-in ratios) especially when also considering the determined uncertainties, these deviations are slightly larger. However, in relation to the Mössbauer spectroscopic results, which differ 6.8 p.p., 4.1 p.p. and 4.5 p.p. for the weigh-in ratios 30/70, 50/50 and 70/30 respectively, the lab-XAFS results bias are of a very similar magnitude or range Δ < 7.0 p.p., in detail: 4.0 p.p. < Δ < 7.0 p.p.. Therefore, the lab-XAFS LCF quantification of adhesive tape prepared α-Fe_2_O_3_/Fe_3_O_4_ samples can considerably compete with Mössbauer spectroscopy, particularly when taking the obtained error values into account. Even so it must also be mentioned that there is a systematic difference between the lab-XAFS and Mössbauer results and accuracies. The lab-XAFS results deviations reflect in experimentally determined lower α-Fe_2_O_3_ and higher Fe_3_O_4_ portions whereas the opposite is the case with the Mössbauer (see Table 1, e.g., *ω*_rel_(α-Fe_2_O_3_)/*ω*_rel_(Fe_3_O_4_) 70/30 ◊ lab-XAFS: 65.6 wt.% α-Fe_2_O_3_/ 43.4 wt.% Fe_3_O_4_, Mössbauer: 75.0 wt.% α-Fe_2_O_3_/ 25.0 wt.% Fe_3_O_4_).

In contrast to the adhesive tape prepared samples, the lab-XAFS LCF results of the α-Fe_2_O_3_/Fe_3_O_4_ model mixtures prepared via the wax pellet technique show smaller deviations of 1.2 p.p. and 2.4 p.p. for the 30/70 and 50/50 target ratios, but a clearly larger bias of 16.5 p.p. for the 70/30 ratio. Thus, regarding the 30/70 and 50/50 mixtures, the lab-XAFS accuracy for the wax pellet samples is higher than for the adhesive tape samples (and also for the Mössbauer spectroscopic measurements), comes close to the XRD magnitude and can clearly compete with Mössbauer spectroscopy and also the XRD. However, regarding to the 70/30 mixture the accuracy is extraordinarily low. One reason for that discrepancy could be slight inhomogeneity in the XANES area, especially the region around the white line. This is explainable by observed inhomogeneity in the prepared sample, thus, a systematic error in the sample-wax pellet preparation. The normalization procedure for this sample was more challenging than for the other mixtures and the reference spectra. Slight changes in the parameter for the post edge line during the normalization process can have significant influence on the height of the oscillations, e.g., the white line and would result in different results of the LCF. When changing the energy range for the LCF, so that the white line area is not included in the fit, the quantitative result agrees much more with the weigh-in ratios with deviation ≤ 10 p.p. Nevertheless, in Table 1, which summarizes the results, just the mass ratios determined with the LCF over the entire energy range are included, as this setting was also used for the analysis of the Mexican iron ore. In addition to that, the quantitative results by the XRD Rietveld refinement have the highest bias as well for the 70/30 mixtures in comparison with the weight in fraction. The deviation with 2.6 pp is over 10 times higher compared to the bias of only 0.2 p.p. for the 30/70 and 50/50 mixtures. In total, the comparison of the results for the α-Fe_2_O_3_/Fe_3_O_4_ model mixture wax pellets and the mixtures prepared on adhesive tape also demonstrate, that XAFS results can be affected by the applied sample preparation technique. Thus, depending on the characteristics of the sample system, different preparation techniques can yield to different accuracies. Reasons for this are for instance the challenges in preparing sufficiently homogenous samples. Since in lab-XAFS spectroscopy the applied X-ray beam covers a sample area which is larger compared to synchrotron-based XAFS, a sufficient homogeneity all across this analyzed area is crucial, especially when quantitative examinations are intended.

Finally, when assessing the accuracy examinations, it must be underlined that the results presented in this study should be viewed as trends. Further examinations with an increased number of samples in context of replicate/ multiple/ parallel determinations (in scope of this study the feasible number of samples was limited, see Table 1 legend) are required to optimize the statistics and to be able to evaluate the accuracy results differentiated into trueness and precision (see ISO 5725-1 [49]). Therefore, a more detailed investigation into the different contributions of systematic (trueness) and random errors (precision) to the particular accuracy of the three species analysis techniques would be feasible. Additionally, also examinations regarding the detections limits (LODs) should be performed because the presented investigations focused on mixtures with both components in concentrations of main component ranges only.

Since the Mexican iron ore was prepared with the adhesive tape method and the statistical uncertainty for the measurement of the XAFS spectra is comparable, in terms of accuracy, it could be assumed, that the bias for this analysis is below 7 p.p. accordingly. Analogously, bias below 3 p.p. for the XRD and 7 p.p. for the Mössbauer analyses could be expected. With regard to the XRD measurements it must be highlighted again, that the model mixtures were just examined with a standard configurated machine using non-ideal Cu Kα radiation. Since the more iron-compound suitable Co Kα radiation was applied for the iron ore measurements, an even higher accuracy could be expected. Nevertheless, because the natural iron ore sample has a more complex composition, interferences (depending on the respective method) as well as different and more pronounced matrix effects on the analytical signals can limit these assumptions to certain extent. Furthermore, other specific sample characteristics can be additional restraints and increase the bias, thus, lowering/ limiting the expected accuracies: Particle sizes can significantly influence XAFS spectra, XRDs and also Mössbauer spectra. For example, the pre-characterization results of the iron ore revealed a particle size slightly exceeding the ideal limit for the XAFS measurements, whereas the particle sizes of the reference substances (and, hence, also the model mixtures) were in the ideal magnitude. Phase impurities, e.g., in the form of isomorphic substitution (in the case of the iron ore sample of this study: Fe(III) partially substituted by Al(III)) are known for their effects on XRD as well as Mössbauer spectra. However, slight phase impurities (Al(III)) magnitudes similar to the iron ore were also the case with the Fe_3_O_4_ reference substance and, as the obtained accuracies demonstrate, did not affect the quantitative results for the model mixtures on a larger scale. Specific for XRD, the presence of amorphous phases must also be mentioned.

In case of the lab-XAFS analysis the results presented in Table 2 showing the averaged LCF results of the Mexican iron ore composition (72 wt.% ± 3 wt.% α-Fe_2_O_3_: 28 wt.% ± 3 wt.% Fe_3_O_4_) are in good agreement with the averaged quantitative XRD results (74 wt.% ± 1 wt.% α-Fe_2_O_3_: 27 wt.% ± 1 wt.% Fe_3_O_4_) because the deviation, especially when also considering the uncertainty of the XAFS results, of Δ ≈ 2 p.p. is very small. This is also the case, when comparing the individual, but as already mentioned very similar *Profex*- and *Topas*-based XRD results (Table S10 in S1 File) to the averaged XAFS outcomes. When just considering the values obtained by *Topas* the deviation gets even smaller (Δ ≈ 1.0 p.p.). Nevertheless, it has also to be mentioned that the deviations partially increase, when also collating the four individual XAFS results (Table S7 in S1 File) and the (averaged or individual) XRD values. Thus, the largest deviation is obtained in the comparison of the XAFS LCF method “in-house algorithm unflattened normalized” and the *Profex*-based XRD result. But all in all, even in this case the deviation of Δ ≈ 4.8 p.p. is relatively small and the general magnitude of all obtained lab-XAFS and XRD weight percent results of about 70% hematite and 30% magnetite is still obvious. This is especially valid when also considering the higher uncertainty of the XAFS result obtained by LCF method “in-house algorithm unflattened normalized” (Table S7 in S1 File). This deviation is still below the estimated bias 7 p.p. by measuring the synthetic α-Fe_2_O_3_, Fe_3_O_4_ and mixtures of both which was described above. However, these slightly varying XAFS results also demonstrate that the quantification results of the lab-XAFS spectroscopy (and also XAFS spectroscopy in general) can be distinctly affected by the chosen evaluation procedure, thus the specific LCF method/ algorithm, not even taken into account differences induced by the pre-processing, e.g., normalization procedure. Nevertheless, such differences can also occur within Rietveld-based XRD quantifications since small distinctions in the applied Rietveld procedure and its parameters impact the results. Regarding to Mössbauer quantifications such effects must be considered as well. In this case as well, fitting procedures of obtained raw data are the crucial fundament/ requirement for both, qualitative and the subsequential quantitative evaluation. However, the folding procedure (performed beforehand the fitting) is an additional potentially affecting step in Mössbauer spectroscopy.

The composition of the Mexican iron ore determined by Mössbauer spectroscopy (57 wt.% ± 1 wt.% α-Fe_2_O_3_: 43 wt.% ± 2 wt.% Fe_3_O_4_) differs clearly from the lab-XAFS and also the XRD values (Mössbauer deviation from XRD Δ ≈ 17.0 p.p.). The conclusion of a higher mass proportion of hematite in the mineral sample is in accordance with the lab-XAFS LCF and XRD Rietveld results in this respect. However, even though considering the quoted uncertainty of the Mössbauer quantification results, the particular mass ratio is significantly different.

### General differences of the three techniques

The reasons for this difference are diverse. One general aspect which is a limiting factor of the Mössbauer results in this study, both, for the model mixtures as well as the iron ore, is the resolving power. The applied *MIMOS II* type spectrometer set-up had just 512 velocity channels and thus folded spectra of 256 data points were used for the evaluations (the fitting procedures and the subsequent qualitative and quantitative analyses). This yields to a spectral resolution which is somewhat reduced compared to Mössbauer spectrometers with 1024 velocity channels (folded spectra of 512 data points) available (see, e.g., the spectra presented in [61], but there, backscattering mode was used which also affects the resolution). Thus, even with an ideal fit, the informative value can be slightly more limited. However, four more aspects, which lead to significantly higher uncertainties of the Mössbauer results particularly for the iron ore sample (but also for the model mixtures) than the stated calculated errors when considering them, are especially important and may be particularly significant. Firstly, an obtained reduced chi-squared $\chi_{\nu }^{2}=4.158$ (Fig 3c) is an indication of a lower fit quality in comparison to the model mixture spectra. For the mixtures distinctively smaller values in the range of about 1.4 to 3.8 (see Fig 3d and S15 in S1 File) were achieved. One sources of this issue can be the chosen analysis/ fitting approach. The applied Lorentzian site analysis requires, e.g., very (infinitely) thin samples since thickness effects can yield to line shapes showing larger deviations from Lorentzian line shapes [62,63]. Thus, it is possible that the line shapes of the measured iron ore Mössbauer spectrum differ more from Lorentzian distributions and alternative fitting approaches (for example Voigt-based fitting analyses) might be an option. A second potential source can be that the iron ore Mössbauer spectrum cannot be completely described by just three subspectra (one for the hematite part and two for the magnetite). If more subspectra are required for an improved fit, this would indicate the presence of additional iron-containing phases in the ore sample. This source might be the major one in the case of the iron ore sample because distinct indications of contents of a third iron species have even been observed in the fitted spectrum as it is. Additionally, the signal-to-noise ratio can also be considered since it cannot be excluded that there are even more iron phases (with very low concentrations) which signals were not separated from the background but affect the reduced chi-squared. However, despite the rule of thumb that higher reduced chi-squared values ($\chi_{\nu }^{2}\gg 1$) indicate poorer fit qualities, the significance of this value should not be overestimated in Mössbauer spectroscopy. Even fits with poorer reduced chi-squared can conform to appropriate fits and describe the sample correctly (for a details see, e.g., [95]).This can also be seen in this study when considering the measured spectrum of the pure iron(III) oxide reference substance ($\chi_{\nu }^{2}=7.748$, but the fit is obviously acceptable). Secondly, the fundamental formula used for the quantification in this study is already an approximation. In this approach several simplified assumptions had been done, for example the widths at half maximum of the absorption peaks were set equal and thickness effects were not considered. Thirdly, just averaged literature values of the required Mössbauer fractions *f* were used in this approximation formula. It has to be claimed that these *f* values can vary greatly due to temperature dependence as well as slightly different sample properties (impurities, particle sizes etc.). Therefore, beforehand the quantification, a complex direct or indirect experimental determination of the recoilless fractions *f* is important if more accurate results shall be obtained [32,35]. However, both, the application of the approximative quantification formula and also the usage of averaged literature based *f* values was also the case with the quantitative Mössbauer examinations of the α-Fe_2_O_3_/Fe_3_O_4_ mixtures. Although for the mixture results the mentioned two aspects are definitely also limiting effects which explain the lower accuracies compared to XRD, the procedure still yielded to relatively good accuracies (see above and Table 1). That proves the general applicability of this approach, but despite this, thickness effects and larger deviations of the actual *f* values from applied averaged literature values could have had a stronger effect in case of the natural iron ore sample. The fourth aspect is the observed presence of another iron species (oxidized magnetite phase) in the ore sample, which overlay parts of the magnetite signal and could not be separated from it in the Mössbauer-fitting procedure in the form of a fourth sextet in course of this study. Therefore, just the measuring data of one of the two magnetite sextets could be utilized in the quantitative Mössbauer evaluation and determination of Fe_3_O_4_. Nevertheless, the same quantification procedure was also applied for the α-Fe_2_O_3_/Fe_3_O_4_ mixtures. In this case, it did not result in distinctively decreased accuracies, but it must also be considered that the level of Fe_3_O_4_ oxidation was certainly smaller than the oxidation grade of the iron ore sample. On the other hand, it must also be highlighted that this issue of an interfering third iron species simultaneously proves the pronounced sensitivity and large capacity of Mössbauer spectroscopy regarding to iron speciation. Neither the lab-XAFS nor the XRD analyses of this study revealed an additional iron containing phase on their own. Even though, a real consideration of this third iron compound could not be achieved during the Mössbauer measurement and fitting, at least a mathematical approximation/ estimation was attempted. Based on the known practical ideal Fe_3_O_4_ area ratio *A*(B-site)/*A*(A-site) = 1.8:1 the contribution of the oxidized magnetite phase Fe_3–x_O_4_ to the obtained total A-site area was calculated (*A*(Fe_3–x_O_4_) ≈ 52498 cts ∙ mm ∙ s^–1^). With the simplified assumption, that this oxidized magnetite phase can be described as maghemite (γ-Fe_2_O_3_, Mössbauer fraction *f*_RT_ = 0.814) [32], a mass ratio of about 25 wt.% maghemite (γ-Fe_2_O_3_) to 75 wt.% magnetite (Fe_3_O_4_) or in sum 49 wt.% hematite (α-Fe_2_O_3_), 38 wt.% magnetite (Fe_3_O_4_) and 13 wt.% maghemite (γ-Fe_2_O_3_) was determined (Table 2, Mössbauer-3). The essential issue of this approach is that the presence of the oxidized magnetite phase is assumed to be the only source of the observed sextet area ratio deviation. Although it is certainly the major source (see description in the results section), the partly Fe(III) substitution by Al(III) in the minerals of the iron ore sample must also be considered. Thus, this additionally calculated/ approximated iron ore composition with three species (Mössbauer-3) should be treated with caution. Nevertheless, in comparison to XRD and XAFS, it could be possible that the mass ratio hematite to magnetite determined by Mössbauer spectroscopy is potentially even the more correct one – especially when considering the possibility that in the XRD as well as XAFS quantifications the maghemite contribution could have been added to the hematite and/ or magnetite signals. For instance, in case of the XRD it is known that the diffraction patterns of all three iron oxides, but particularly magnetite and maghemite show similarities and are difficult to distinguish [55,89]. Since magnetite and maghemite have very similar crystal structures (Fe_3_O_4_: inverse spine-type structure, γ-Fe_2_O_3_: defect spine-structure) the XRD peak quantities, positions and intensities are of pronounced similarity. Just some additional very weak reflections at low 2*θ* values (respectively high lattice plane distances *d*), which are detectable in some cases (under ideal measurement conditions and depending on the sample composition), are specific for maghemite. Despite the application of optimized measurement conditions (Co Kα radiation) these distinctive maghemite signals were not resolved in the diffractogram of the iron ore of this study (see Fig 2 and Fig S14a-b in S1 File area 2*θ* < 35°). Thus, maghemite could not be detected in the ore sample. For the XAFS subsequent measurements with the inclusion of γ-Fe_2_O_3_ as a third reference standard have been performed. The γ-Fe_2_O_3_[96] was obtained from *Sigma Aldrich* and has been undergone equal extensive pre-characterization (see [66]) as the references α-Fe_2_O_3_ [64] and Fe_3_O_4_ [65]**.** The resolving power of the lab spectrometer is sufficiently high to display disparities between γ-Fe_2_O_3_ and Fe_3_O_4_ in the XANES area (see SI Fig S9a in S1 File), although a comparison with synchrotron XAFS spectra (see SI Fig S9b in S1 File) indicates that synchrotron spectra can be slightly more beneficial for a differentiation of these two iron oxides. Particularly, the potentially very high resolution of synchrotron XAFS might be advantageous or even required, for example, in cases in which one of the two compounds is just contained in very low concentrations (combination: high Fe_3_O_4_ content + very low γ-Fe_2_O_3_ content or vice versa). The lab-XAFS LCF has been performed with the two references as in the prior measurements and additionally with γ-Fe_2_O_3_. The results can be seen in Fig S10 a and b in S1 File and are discussed in detail in the SI. For the two standard LCF the results are in accordance with the prior measurements and analysis. Using the additional γ-Fe_2_O_3_ as a reference results in an LCF with a slightly better fit by considering the reduced chi-squared given by the *ATHENA* software. The resulting composition of the Mexican iron ore is in this case 56 wt.% ± 3 wt.% α-Fe_2_O_3_, 17 wt.% ± 5 wt.% Fe_3_O_4_ and 27 wt.% ± 3 wt.% γ-Fe_2_O_3_ (Table 2, XAFS-3). Just focused on the α-Fe_2_O_3_ and Fe_3_O_4_ parts, this results in a ratio of about 77 wt.% α-Fe_2_O_3_ to 23 wt.% Fe_3_O_4_. Therefore, despite the consideration of the third species in the lab-XAFS LCF, the distinct difference to the Mössbauer spectroscopic results persists. In this case, the discrepancies of lab-XAFS and Mössbauer results are between 7 p.p. and 21 p.p. and may have various origins. As it has already been mentioned, the Mössbauer results are based on a very simplified assumption since the γ-Fe_2_O_3_ was not/ could not be fitted separately in case of the ore sample of this study. To do so, further elaborated measurements in form of temperature-dependent experiments or measurements by applying an external magnetic field could be beneficial. Furthermore, as already mentioned above, for a correct determination of relative concentrations the spectrometer has to be well calibrated or all the hyperfine parameters have to be known and even then the relative error will be about 5% [97]. However, in case of the lab-XAFS it must be considered that also contents of small amounts of such an oxidized Fe_3_O_4_ phase in the Fe_3_O_4_ reference substance, applied in the qualitative and quantitative spectrum evaluation, cannot be ruled out completely (see Mössbauer results) – a limitation that must be taken into account in both attempts, the LCF based on two as well as the one based on three reference substances because in both cases the same Fe_3_O_4_ reference substance was applied. Furthermore, the estimated bias for LCF with this Lab-XAFS spectrometer of ≤ 7 p.p. (determined for LCF with two species, α-Fe_2_O_3_ and Fe_3_O_4_) is likely to increase (decreasing accuracy) when considering additional species in the LCF with similar spectral characteristics. To investigate this, further examinations on a set of model mixtures containing α-Fe_2_O_3_, Fe_3_O_4_ and γ-Fe_2_O_3_, thus, all three species instead of just two, are required. On top of that, also the (additional) consideration of iron(III) hydroxides, or rather iron(III) oxide-hydroxides such as α-FeO(OH) and/ or γ-FeO(OH) in model mixture investigations are interesting for future lab-XAFS examinations. The first reason is that also in case of these compounds a relatively distinct similarity to the iron oxides is present in the XAFS spectra [52]. In contrast, in the XRD [55] as well as the Mössbauer spectra [32] a distinction from the oxides is relatively simple in plenty of cases. The second reason is an improved applicability in practice. In the natural iron ore sample investigated in scope of this study noticeable amounts of iron hydroxide compounds were not detected (at least not unequivocally and/ or within the detection limits of the analyses performed – thus, minor or trace amounts of iron hydroxides are still not excluded). However, in general, the paragenesis of the iron oxide minerals hematite, magnetite and maghemite with individual minerals of these iron(III) oxide-hydrates (“Fe_2_O_3_· x H_2_O”), such as goethite (α-FeO(OH)) and lepidocrocite (γ-FeO(OH)), or mixtures of them (e.g., limonite) is very common in geological samples due to secondary mineral/ rock formation processes (e.g., weathering) with participation of water.

A compact comparison of the properties of all three methods is given in Table S15 in S1 File to provide a comprehensive overview of the differences between the methods.

### Discussion on necessary pre-characterization

Another aspect, which must be considered in the comparative discussion of the lab-XAFS in relation to the other two applied techniques is the effort of the quantitative (and concurrently the qualitative) evaluation of the measured data. While performing qualitative analyses and especially quantitative LCF on lab-XAFS spectra and synchrotron XAFS spectra, one of the most crucial points is the knowledge about the sample regarding possible contained materials or substances to select suitable reference substances [98]. Actually, also in Mössbauer spectroscopy pre-knowledge on a sample can be necessary or, at least, beneficial to set appropriate starting values for the fitting procedures or to evaluate the plausibility of the results [32,99]. For example, in cases of samples with very complex compositions and/ or overlapping subspectra (due to similar line shapes/ hyperfine parameters) the fitting processes get very complicated. On top of that, in such cases different subspectra fits can sometimes yield to total fits of equal or similar quality, when just relying on the mathematical process [99]. However, a selection and application of suitable reference substances is rarely performed in Mössbauer spectroscopy whereas in XAFS this is a crucial requirement. In this study, detailed pre-characterizations, especially qualitative XRD, had already led to a composition of the Mexican iron ore sample of magnetite (Fe_3_O_4_) and hematite (α-Fe_2_O_3_) and, thus, homologous reference substances were applied. XRD and, with some limitations, also Mössbauer spectroscopy can revert to large established reference databases (XRD: Powder Diffraction Files of the *ICDD* and *ICSD*, Mössbauer spectroscopy: e.g., the *MEDC database*, the *Mössbauer Effect Reference and Data Journal* and the *Mössbauer Mineral Handbook* of the *Mössbauer Effect Data Center* [100]) and can be performed as virtually standardless and, thus, relatively fast methods in a lot of qualitative and quantitative analyses. This especially applies to cases of relatively simple samples or simple scientific questions such as the one of this study. In contrast, XAFS and especially lab-XAFS still lack comparable elaborated databases. And even with such a database at hand, the specific spectrometer which was used in generating the database’s spectra might have a significant influence on the spectrum itself, thus complicating the LCF approach. Though there are numerous studies containing synchrotron and, since recent times in continuously increasing number, also lab-XAFS spectra data of various compounds, it is still necessary to measure reference substances parallel to the analysis samples in a lot of cases. This is most notably valid when also intending to perform quantification (by LCF), which increases the analysis labor and time cost. However, it must be noted that, sometimes, at least, quantitative XRD is also performed by standard-based methods (with external or internal standards, e.g., the reference intensity ratio method with α-Al_2_O_3_ standard [25]) instead of the standardless Rietveld method. Furthermore, in case of Mössbauer spectroscopy it is often imperative to perform more costly additional measurements (e.g., determination of the sample specific recoilless fraction *f*) as well as to optimize the calculation parameters/ process (e.g., consideration of the thickness effects) to get more accurate and statistically firmer quantitative results, as the measurements of this study also clearly illustrate**.**

### Discussion on quantification method

The last aspect, which is revealed by the measurements of this study are the different sorts of quantification realizable by the three speciation methods. It must be pointed out that the performed lab-XAFS evaluation by LCF, but also the Mössbauer analysis just yielded to relative quantitative results. These relative results were also limited to the iron species of the sample and therefore only the quantitative relative ratios of these specific species were determined. In contrast, the XRD analysis additionally provided information on more species of the sample, and these were incorporated in the quantitative Rietveld refinement. Although XAFS spectroscopy in general and also lab-XAFS spectrometers in particular are suitable for analyzing many elements and, thus, numerous different species (e.g., the lab-XAFS setup used in this study currently has an accessible energy range of *E* = 4.0–19.0 keV [50]), in a single run, the measurement is restricted to/ focused on a simultaneously measurable energy range of a few hundred eV around an element specific X-ray absorption edge. This means that a qualitative and quantitative multi elemental and species analysis cannot be performed in a single run and requires different measurements, each performed with suited reference materials, respectively. However, Mössbauer spectroscopy is clearly more limited since a multi element/ species analysis, like the XAFS, demands multiple different measurements but is additionally limited to only a few potentially accessible elements. This decreases the potential to consider species of different elements in a Mössbauer-based quantification further more. In contrast to this, XRD enables multi species analyses in a single measurement run with almost no species limitation, except, for example, the thresholds given by the detection limits, the presence of X-ray amorphous phases or the state of matter (XAFS does not have the latter two). Nevertheless, it must also be preconceived that, although Rietveld-based XRD quantification can incorporate numerous species simultaneously, it still remains a ratio quantification, because just the detected and assigned analytes are assumed as the overall sample composition, similar to standardless fundamental parameter based XRF quantification. In cases of simple sample compositions, of samples containing small amounts of secondary or trace ingredients and especially of samples, which just consist of solid crystalline substances, these Rietveld-based relative quantification can get very close to a full relative quantification. The term full relative quantification relates to a quantification in which all sample components are directly or at least indirectly considered, such as the standard-based (external standards, standard addition or internal standards) determinations of amount of substance concentrations, mass concentrations or mass fractions of elements or species where the total volume or mass of the sample is taken into account. Such real full relative XRD quantifications are in some cases feasible using more costly standard-based XRD methods [25]. Regarding lab-XAFS (and XAFS in general) absolute quantifications would also require the application of standard-based methods, for instance in the context of external standard calibration series (either consisting of one or more species of interest). However, this leads to the general challenges and actual limitations of purchasing or preparing suitable reference materials (regarding to contained elements/ species, element/ species concentrations, matrix adjustment etc.) for applications as (external) standards in solid state spectroscopic techniques.

## Conclusion

The capability of lab-XAFS performing quantitative analysis on synthetic α-Fe_2_O_3_/Fe_3_O_4_ model mixtures as well as on an iron oxides containing ore sample was shown in comparison to XRD and Mössbauer spectroscopy.

Despite the high similarity of the references XAFS spectra Fe_3_O_4_ and α-Fe_2_O_3_ the quantitative lab-XAFS results for the iron oxide model mixtures reveal accuracies similar to those obtained for the Mössbauer (bias < 7 p.p.) and XRD (bias < 3 p.p.) measurements of the mixtures. Thus, in total, the mass ratios *ω*_rel_(α-Fe_2_O_3_)/*ω*_rel_(Fe_3_O_4_) of the low complexity α-Fe_2_O_3_/Fe_3_O_4_ model mixtures determined by quantitative lab-XAFS are in a relatively good agreement with the Mössbauer and XRD results. However, the results also show that the achievable lab-XAFS accuracy can distinctively be affected by the applied sample preparation technique. In the presented case, the results obtained for the wax pellet prepared samples show a slightly higher accuracy (with the exception of one sample due to a systematic error which occurred in the preparation) than those for the adhesive tape prepared ones.

Regarding to the more complex Mexican iron ore sample the situation is significantly less clear and more complicated. The (averaged) lab-XAFS results of the composition with 72 wt.% ± 3 wt.% hematite (α-Fe_2_O_3_) and 28 wt.% ± 3 wt.% magnetite (Fe_3_O_4_) are in a good agreement with the quantitative XRD results, while the quantitative results by Mössbauer spectroscopy deviate by more than 20% (or 10 p.p.) from the lab-XAFS and XRD results. The causes for that large discrepancy (approximations in fundamental formula, average literature values, etc.) have been explained in detail in the above section. Even when showing a strong deviation in the quantitative results for the iron ore sample, it has to be highlighted that Mössbauer was the only one of the three applied instrumental techniques that could reveal the probable presence of a third iron species, maghemite (γ-Fe_2_O_3_), with a low content. This shows the pronounced sensitivity and capacity of this method with regard to qualitative species analysis of iron containing samples. Although XRD cannot (or just barely) distinguish between the revealed γ-Fe_2_O_3_ and the Fe_3_O_4_, XAFS can qualitatively and quantitatively differentiate between these species if it is known that they must be taken into account. These results clearly demonstrate that knowledge of the possible species in an analyzed sample is crucial for LCF XAFS.

Additionally, the results show three more aspects. Firstly, the Mexican iron ore sample is a case in point for the discrepancies which can occur between simple ideal/ model sample systems and “real” sample systems. Analytics of “real” systems, such as natural samples, often bear numerous additional challenges. Thus, the results of this study present that a consideration of such “real” sample systems in addition to model systems when benchmarking different analysis techniques can be of great value to get a more in-depth view on the strength and weaknesses of each method. Secondly, the reported issues in analyzing the iron ore sample also clearly demonstrate how challenging qualitative and quantitative examinations of samples containing many different iron oxides still are. This applies to all three analysis techniques utilized in this study and is especially emphasized by the outcome that the composition of the Mexican iron ore is still not completely elucidated. Thirdly, the results of this study demonstrate again that in many cases one individual method cannot answer an analytical question sufficiently. A simultaneous application and combination of different analysis techniques is required very often. Thus, in the case of this benchmarking study lab-XAFS, XRD and Mössbauer spectroscopy did not just competed but rather complemented one another. This becomes particularly clear when taking the successful consideration of γ-Fe_2_O_3_ as an additional third iron species in the XAFS LCF, after the Mössbauer spectroscopy had attested the probable presence of this phase, into account.

It can be concluded that quantitative lab-XAFS, using LCF of reference spectra, can compete with the other two methods, especially for samples with low complexity. Therefore, it could be used as an alternative or better complementary method. Nevertheless, it must be highlighted that this conclusion is limited on the analyses of main components. Comparative investigations into minor components and, hence, the detection limits (LODs) of lab-XAFS, XRD and Mössbauer spectroscopy must be performed in future studies to expand and substantiate this conclusion. Lab-XAFS and Mössbauer spectroscopy are limited to analyzing only one element at a time, unlike XRD. However, Mössbauer spectroscopy is restricted to solid phases, while XAFS has no such limitation. XRD is blind to amorphous phases, but XAFS requires more prior knowledge of the sample. Therefore, thepresented results complement the ongoing developments and improvements of laboratory XAFS setups and show once more its capabilities as an everyday tool for laboratory research finding its place side-by-side with and complementing well established methods such as Mössbauer spectroscopy and XRD.[1,2] The ongoing spread of laboratory XAFS spectrometers and progressive construction and expansion of XAFS databases will accelerate the establishment of this method as a tool for species analysis in research but also routine analytics.

## Supporting information

S1 File The data supporting this article have been included as part of the Supplementary Information file: Sample pre-characterizations, XAFS sample preparation/ qualitative analyses/ LCF, Mössbauer hyperfine parameters/ quantification, XRD quantification, individual quantitative results. See DOI: 10.1039/x0xx00000x.
(PDF)

## References

[1] Malzer W, Schlesiger C, Kanngießer B. A century of laboratory X-ray absorption spectroscopy – A review and an optimistic outlook. Spectrochimica Acta Part B: Atomic Spectroscopy. 2021;177:106101. doi: 10.1016/j.sab.2021.106101

[2] Zimmermann P, Peredkov S, Abdala PM, DeBeer S, Tromp M, Müller C, et al. Modern X-ray spectroscopy: XAS and XES in the laboratory. Coordination Chemistry Reviews. 2020;423:213466. doi: 10.1016/j.ccr.2020.213466

[3] Legall H, Stiel H, Schnürer M, Pagels M, Kanngießer B, Müller M. An efficient x-ray spectrometer based on thin mosaic crystal films and its application in various fields of x-ray spectroscopy. J Appl Crystallogr. 2009;42:572–9. doi: 10.1107/S0021889809006803

[4] Schlesiger C, Anklamm L, Stiel H, Malzer W, Kanngießer B. Xafs spectroscopy by an x-ray tube based spectrometer using a novel type of hopg mosaic crystal and optimized image processing. J Anal At Spectrom. 2015;30:1080–5. doi: 10.1039/C4JA00303A

[5] Seidler GT, Mortensen DR, Remesnik AJ, Pacold JI, Ball NA, Barry N, et al. A laboratory-based hard x-ray monochromator for high-resolution x-ray emission spectroscopy and x-ray absorption near edge structure measurements. Rev Sci Instrum. 2014;85(11):113906. doi: 10.1063/1.4901599 25430123

[6] Taguchi T, Shinoda K, Tohji K. Customization of an inhouse XAFS spectrometer for sulfur measurement. Phys Scr. 2005;1017. doi: 10.1238/physica.topical.115a01017

[7] Németh Z, Szlachetko J, Bajnóczi ÉG, Vankó G. Laboratory von Hámos X-ray spectroscopy for routine sample characterization. Rev Sci Instrum. 2016;87(10):103105. doi: 10.1063/1.4964098 27802722

[8] Bès R, Ahopelto T, Honkanen A-P, Huotari S, Leinders G, Pakarinen J, et al. Laboratory-scale X-ray absorption spectroscopy approach for actinide research: Experiment at the uranium L3-edge. Journal of Nuclear Materials. 2018;507:50–3. doi: 10.1016/j.jnucmat.2018.04.034

[9] Honkanen A-P, Ollikkala S, Ahopelto T, Kallio A-J, Blomberg M, Huotari S. Johann-type laboratory-scale x-ray absorption spectrometer with versatile detection modes. Rev Sci Instrum. 2019;90(3):033107. doi: 10.1063/1.5084049 30927829

[10] Jahrman EP, Holden WM, Ditter AS, Mortensen DR, Seidler GT, Fister TT, et al. An improved laboratory-based x-ray absorption fine structure and x-ray emission spectrometer for analytical applications in materials chemistry research. Rev Sci Instrum. 2019;90(2):024106. doi: 10.1063/1.5049383 30831699

[11] de Broglie M. Recherches sur la diffraction des rayons de Röntgen par les milieux cristallins. Radium (Paris). 1913;10(8):245–9. doi: 10.1051/radium:01913001008024500

[12] Fricke. H. The K-Characteristic Absorption Frequencies for the Chemical Elements Magnesium to Chromium. Phys Rev. 1920;16(3):202–15. doi: 10.1103/physrev.16.202

[13] Hertz G. Über Absorptionslinien in der L-serie. Z für Phys. 1920;3:19–25.

[14] Sayers DE, Stern EA, Lytle FW. New technique for investigating noncrystalline structures: Fourier analysis of the extended x-ray—absorption fine structure. Phys Rev Lett. 1971;27:1204–7. doi: 10.1103/PhysRevLett.27.1204

[15] Stern EA. Theory of the extended x-ray-absorption fine structure. Phys Rev B. 1974;10:3027–37. doi: 10.1103/PhysRevB.10.3027

[16] Lytle FW, Sayers DE, Stern EA. Extended x-ray-absorption fine-structure technique. II. Experimental practice and selected results. Phys Rev B. 1975;11(12):4825–35. doi: 10.1103/physrevb.11.4825

[17] Lytle F. First X-ray Absorption Spectroscopy at SSRP in 1974. Synchrotron Radiation News. 2015;28(4):30–3. doi: 10.1080/08940886.2015.1059236

[18] Moya-Cancino JG, Honkanen A-P, van der Eerden AMJ, Schaink H, Folkertsma L, Ghiasi M, et al. In-situ X-Ray Absorption Near Edge Structure Spectroscopy of a Solid Catalyst using a Laboratory-Based Set-up. ChemCatChem. 2019;11(3):1039–44. doi: 10.1002/cctc.201801822 31007776 PMC6471006

[19] Moya–Cancino J, Honkanen A, vanderEerden A, Schaink H, Folkertsma L, Ghiasi M. Elucidating the K–edge X–ray absorption near–edge structure of cobalt carbide. ChemCatChem. 2019;11:3042–5. doi: 10.1002/cctc.201900434 PMC6471006 31007776

[20] Jahrman E, Pellerin L, Ditter A, Bradshaw L, Fister T, Polzin B, et al. Laboratory-based x-ray absorption spectroscopy on a working pouch cell battery at industrially-relevant charging rates. J Electrochem Soc. 2019;166:A2549–55. doi: 10.1149/2.0721912jes

[21] Yamamoto T, Teramachi A, Orita A, Kurimoto A, Motoi T, Tanaka T. Generation of Strong Acid Sites on Yttrium-Doped Tetragonal ZrO2-Supported Tungsten Oxides: Effects of Dopant Amounts on Acidity, Crystalline Phase, Kinds of Tungsten Species, and Their Dispersion. J Phys Chem C. 2016;120(35):19705–13. doi: 10.1021/acs.jpcc.6b05388

[22] Menezes PW, Walter C, Hausmann JN, Beltrán-Suito R, Schlesiger C, Praetz S, et al. Boosting Water Oxidation through In Situ Electroconversion of Manganese Gallide: An Intermetallic Precursor Approach. Angew Chem Int Ed Engl. 2019;58(46):16569–74. doi: 10.1002/anie.201909904 31483557 PMC6899514

[23] Bajnóczi ÉG, Németh Z, Vankó G. Simultaneous Speciation, Structure, and Equilibrium Constant Determination in the Ni2+-EDTA-CN- Ternary System via High-Resolution Laboratory X-ray Absorption Fine Structure Spectroscopy and Theoretical Calculations. Inorg Chem. 2017;56(22):14220–6. doi: 10.1021/acs.inorgchem.7b02311 29116773

[24] Motz D, Praetz S, Schlesiger C, Henniges J, Böttcher F, Hesse B, et al. Examining iron complexes with organic ligands by laboratory XAFS. J Anal At Spectrom. 2023;38(2):391–402. doi: 10.1039/d2ja00351a

[25] Spieß L, Teichert G, Schwarzer R, Behnken H, Genzel C. Moderne Röntgenbeugung: Röntgendiffraktometrie für Materialwissenschaftler, Physiker und Chemiker. 2., überarb. und erw. Aufl. ed. Vieweg Teubner. 2009.

[26] Borchardt-Ott W. Kristallographie. Springer Berlin Heidelberg. 2009. doi: 10.1007/978-3-540-78271-1

[27] Ameh ES. A review of basic crystallography and x-ray diffraction applications. Int J Adv Manuf Technol. 2019;105(7–8):3289–302. doi: 10.1007/s00170-019-04508-1

[28] Sharma R, Bisen D, Shukla U, Sharma B. X-ray diffraction: a powerful method of characterizing nanomaterials. Recent Res Sci Technol. 2012.

[29] Gütlich P, Bill E, Trautwein AX. Mössbauer Spectroscopy and Transition Metal Chemistry. Springer Berlin Heidelberg. 2011. doi: 10.1007/978-3-540-88428-6

[30] Nasu S. General Introduction to Mössbauer Spectroscopy. In: Yoshida Y, Langouche G, editors. Mössbauer Spectroscopy. Berlin, Heidelberg: Springer Berlin Heidelberg; 2013. pp. 1–22. doi: 10.1007/978-3-642-32220-4‗

[31] Gütlich P, Garcia Y. Chemical Applications of Mössbauer Spectroscopy. In: Yoshida Y, Langouche G, editors. Mössbauer Spectroscopy. Berlin, Heidelberg: Springer Berlin Heidelberg; 2013. pp. 23–89. doi: 10.1007/978-3-642-32220-4‗

[32] Vandenberghe RE, de Grave E. Application of Mössbauer Spectroscopy in Earth Sciences. In: Yoshida Y, Langouche G, Editors. Mössbauer Spectroscopy. Berlin, Heidelberg: Springer Berlin Heidelberg; 2013. pp. 91–185. doi: 10.1007/978-3-642-32220-4

[33] Greneche J-M. The Contribution of 57Fe Mössbauer Spectrometry to Investigate Magnetic Nanomaterials. In: Yoshida Y, Langouche G, editors. Mössbauer Spectroscopy. Berlin, Heidelberg: Springer Berlin Heidelberg; 2013. pp. 187–241. doi: 10.1007/978-3-642-32220-4‗

[34] Shinjo T, Mibu K. Magnetic Multilayers and Interfaces. In: Yoshida Y, Langouche G, editors. Mössbauer Spectroscopy. Berlin, Heidelberg: Springer Berlin Heidelberg; 2013. pp. 243–265. doi: 10.1007/978-3-642-32220-4

[35] Scott B, Brown CAM, Dunlap RA, Obrovac MN. Quantitative composition determination by Mössbauer spectroscopy. MRS Communications. 2020;10(1):123–8. doi: 10.1557/mrc.2019.158

[36] Enamullah M, Renz F, El-Ayaan U, Wiesinger G, Linert W. Experimental spin-crossover investigations on charged and neutral iron(II) complexes with 4-substituted-2,6-bis-(benzimidazol-2′-yl)pyridine. Vib Spectrosc. 1997;14:95–104.

[37] Eissa NA, Hassaan MY, Sallam HA, Salah SH, Abo-El-Enein SA. Mössbauer spectroscopy in cement manufacture. J Mater Sci Lett. 1984;3:88–90. doi: 10.1007/BF00720084

[38] Lilkov V, Petrov O, Tzvetanova Y, Savov P, Kadiyski M. Mössbauer, XRD, and complex thermal analysis of the hydration of cement with fly ash. J Spectrosc. 2013;2013:1–9. doi: 10.1155/2013/231843

[39] Costa BFO, Blumers M, Sansano A, Klingelhöfer G, Rull F, Lehmann R, et al. Klimt artwork: material investigation by backscattering Fe-57 Mössbauer and Raman spectroscopy. Hyperfine Interact. 2014;226(1–3):621–7. doi: 10.1007/s10751-013-0998-z

[40] Hanslmeier A. Einführung in Astronomie und Astrophysik. Berlin, Heidelberg: Springer Berlin Heidelberg. 2020. doi: 10.1007/978-3-662-60413-7

[41] Bethge K, Walter G, Wiedermann B. Kernphysik. Springer Berlin Heidelberg. 2008. doi: 10.1007/978-3-540-74567-9

[42] Demtröder W. Experimentalphysik 4. Berlin, Heidelberg: Springer Berlin Heidelberg. 2010. doi: 10.1007/978-3-642-01598-4

[43] Riedel E, Janiak C. Anorganische Chemie. 8 ed. Berlin: de Gruyter. 2011.

[44] Neukirchen F, Ries G. Die Welt der Rohstoffe: Lagerstätten, Förderung und wirtschaftliche Aspekte. Springer Spektrum. 2014.

[45] Bunker G. Introduction to XAFS: A practical guide to X-ray absorption fine structure spectroscopy. Cambridge and New York: Cambridge University Press. 2010.

[46] Schnohr CS, Ridgway MC. X-Ray Absorption Spectroscopy of Semiconductors. Springer Berlin Heidelberg. 2015. doi: 10.1007/978-3-662-44362-0

[47] Kas JJ, Jorissen K, Rehr JJ. Real‐Space Multiple‐Scattering Theory of X‐Ray Spectra. X‐Ray Absorption and X‐Ray Emission Spectroscopy. Wiley. 2016. 51–72. doi: 10.1002/9781118844243.ch3

[48] Joly Y, Grenier S. Theory of X‐Ray Absorption Near Edge Structure. X‐Ray Absorption and X‐Ray Emission Spectroscopy. Wiley. 2016. 73–97. doi: 10.1002/9781118844243.ch4

[49] ISO 5725-1 - Accuracy (trueness and precision) of measurement methods and results - Part 1: General principles and definitions. Switzerland; 2023. Available: https://www.iso.org/obp/ui/#iso:std:iso:5725:-1:ed-2:v1:en

[50] Schlesiger C, Praetz S, Gnewkow R, Malzer W, Kanngießer B. Recent progress in the performance of HAPG based laboratory EXAFS and XANES spectrometers. J Anal At Spectrom. 2020;35(10):2298–304. doi: 10.1039/d0ja00208a

[51] Ravel B, Newville M. Athena, Artemis, Hephaestus: data analysis for X-ray absorption spectroscopy using IFEFFIT. J Synchrotron Radiat. 2005;12(Pt 4):537–41. doi: 10.1107/S0909049505012719 15968136

[52] Newville M. Larch: An Analysis Package for XAFS and Related Spectroscopies. J Phys: Conf Ser. 2013;430:012007. doi: 10.1088/1742-6596/430/1/012007

[53] Rietveld HM. A profile refinement method for nuclear and magnetic structures. J Appl Crystallogr. 1969;2:65–71. doi: 10.1107/S0021889869006558

[54] Rodríguez-Carvajal J. Fullprof: a program for rietveld refinement and pattern matching analysis. In: Satellite Meeting on Powder Diffraction of the XV Congress of the IUCr. 1990.

[55] Mos Y, Vermeulen A, Buisman C, Weijma J. X-ray diffraction of iron containing samples: the importance of a suitable configuration. Geomicrobiol J. 2018;35:511–7. doi: 10.1080/01490451.2017.1401183

[56] Gražulis S, Chateigner D, Downs RT, Yokochi AFT, Quirós M, Lutterotti L, et al. Crystallography Open Database - an open-access collection of crystal structures. J Appl Crystallogr. 2009;42(Pt 4):726–9. doi: 10.1107/S0021889809016690 22477773 PMC3253730

[57] Bergmann J, Friedel P, Kleeberg R. BGMN – a new fundamental parameter based Rietveld program for laboratory X-ray sources, its use in quantitative analysis and structure investigations. IUCrJ. 1998;5:ß.

[58] Doebelin N, Kleeberg R. Profex: a graphical user interface for the Rietveld refinement program BGMN. J Appl Crystallogr. 2015;48(Pt 5):1573–80. doi: 10.1107/S1600576715014685 26500466 PMC4603273

[59] Runčevski T, Dinnebier RE, Leineweber A, Evans JSO. Rietveld refinement practical powder diffraction pattern analysis using TOPAS. J Appl Crystallogr. 2019;52:1238–9. doi: 10.1107/S1600576719011178

[60] Granone LI, Ulpe AC, Robben L, Klimke S, Jahns M, Renz F, et al. Effect of the degree of inversion on optical properties of spinel ZnFe2O4. Phys Chem Chem Phys. 2018;20(44):28267–78. doi: 10.1039/c8cp05061a 30398245

[61] Klingelhöfer G, Morris R, Bernhardt B, Rodionov D, De Souza P, Squyres S. Athena MIMOS II Mössbauer spectrometer investigation. J Geophys Res Planets. 2003;108:2003JE002138. doi: 10.1029/2003JE002138

[62] Klencsár Z. Mössbauer spectrum analysis by evolution algorithm. Nucl Instrum Methods Phys Res Sect B Beam Interact Mater At. 1997;129:527–33. doi: 10.1016/S0168-583X(97)00314-5

[63] Lagarec K, Rancourt D. Recoil – mössbauer spectral analysis software for windows, version 1.0. Departments of Physics University of Ottawa. 1998.

[64] Honeywell/Fluka. iron(III) oxide (310050), powder, <5µm, ≥99%. Honeywell, editor. Available: https://lab.honeywell.com/shop/iron-iii-oxide-310050

[65] Sigma-Aldrich. iron(II,III) oxide (310069): powder, < 5µm, 95%. Available: https://www.sigmaaldrich.com/DE/de/product/aldrich/310069

[66] Xu H, Zhang Y, Zhang Z, Wang J, Shen C, Wu Z, et al. Development and validation of a nomogram for predicting prostatic urethral involvement in bladder cancer. Sci Rep. 2025;15(1):10431. doi: 10.1038/s41598-025-95684-6 40140488 PMC11947163

[67] Chantler C, Olsen K, Dragoset R, Chang J, Kishore A, Kotochigova S. X-ray form factor, attenuation, and scattering tables (version 2.1). Natl Inst Stand Technol. 2005. https://www.nist.gov/pml/x-ray-form-factor-attenuation-and-scattering-tables

[68] Cammann K. Instrumentelle Analytische Chemie: Verfahren, Anwendungen, Qualitatssicherung. Heidelberg: Springer. 2010.

[69] Okrusch M, Matthes S. Mineralogie. Springer Berlin Heidelberg. 2014. doi: 10.1007/978-3-642-34660-6

[70] Petrovský E, Kropáček V, Dekkers MJ, deBoer C, Hoffmann V, Ambatiello A. Transformation of hematite to maghemite as observed by changes in magnetic parameters: Effects of mechanical activation?. Geophysical Research Letters. 1996;23(12):1477–80. doi: 10.1029/96gl01411

[71] Cornell RM, Schwertmann U. The Iron Oxides. Wiley. 2003. doi: 10.1002/3527602097

[72] Choudhary A, Khandelwal N, Ganie ZA, Darbha GK. Influence of magnetite and its weathering originated maghemite and hematite minerals on sedimentation and transport of nanoplastics in the aqueous and subsurface environments. Sci Total Environ. 2024;912:169132. doi: 10.1016/j.scitotenv.2023.169132 38070555

[73] Wilke M, Farges F, Petit P-E, Brown GE Jr, Martin F. Oxidation state and coordination of Fe in minerals: An FeK-XANES spectroscopic study. American Mineralogist. 2001;86(5–6):714–30. doi: 10.2138/am-2001-5-612

[74] Matsukawa T, Obashi M, Nakai S, Sugiura C. The k-absorption spectra of fes2, cos2 and nis2. Jpn J Appl Phys. 1978;18:184–6.

[75] Petiau J, Sainctavit Ph, Calas G. K X-ray absorption spectra and electronic structure of chalcopyrite CuFeS2. Materials Science and Engineering: B. 1988;1(3–4):237–49. doi: 10.1016/0921-5107(88)90004-9

[76] Lennie A, Vaughan D. Spectroscopic studies of iron sulfide formation and phase relations at low temperatures. In: Geol Soc Proc. 1996. 117–32.

[77] Holleman AF, Wiberg N. Lehrbuch der Anorganischen Chemie: [mit 188 Tabellen]. 102., stark umgearb. und verb. Aufl. Berlin [u.a.]: de Gruyter; 2007.

[78] Binnewies M, Finze M, Jäckel M, Schmidt P, Willner H, Rayner-Canham G. Allgemeine und Anorganische Chemie. Springer Berlin Heidelberg. 2016. doi: 10.1007/978-3-662-45067-3

[79] Müller U. Anorganische Strukturchemie. 6., aktualisierte Auflage ed. Vieweg Teubner Verlag/GWV Fachverlage GmbH. 2008.

[80] Ravel B. Normalization. ATHENA: XAS Data Processing. 2016. https://bruceravel.github.io/demeter/documents/Athena/bkg/norm.html#the-normalization-algorithm

[81] Calvin S, Furst K. Xafs for everyone. Boca Raton, Fla.: CRC Press. 2013.

[82] Toby BH. Rfactors in Rietveld analysis: How good is good enough?. Powder Diffr. 2006;21(1):67–70. doi: 10.1154/1.2179804

[83] E11 Committee. Practice for Use of the Terms Precision and Bias in ASTM Test Methods. West Conshohocken, PA: ASTM International; doi: 10.1520/E0177-13

[84] Siddique M, Ahmed E, Butt NM. Particle size effect on Mössbauer parameters in γ-Fe2O3 nanoparticles. Physica B: Condensed Matter. 2010;405(18):3964–7. doi: 10.1016/j.physb.2010.06.039

[85] Lemine OM, Sajieddine M, Bououdina M, Msalam R, Mufti S, Alyamani A. Rietveld analysis and Mössbauer spectroscopy studies of nanocrystalline hematite α-Fe2O3. Journal of Alloys and Compounds. 2010;502(2):279–82. doi: 10.1016/j.jallcom.2010.04.175

[86] Zakharova IN, Shipilin MA, Alekseev VP, Shipilin AM. Mössbauer study of maghemite nanoparticles. Tech Phys Lett. 2012;38(1):55–8. doi: 10.1134/s1063785012010294

[87] Stevens J, Khasanov A, Miller J, Pollak H, Li Z. Mössbauer mineral handbook. Mössabuer Effect Data Center (The University of North Carolina at Asheville). 2005.

[88] Daniels JM, Rosencwaig A. Mössbauer spectroscopy of stoichiometric and non-stoichiometric magnetite. Journal of Physics and Chemistry of Solids. 1969;30(6):1561–71. doi: 10.1016/0022-3697(69)90217-0

[89] Winsett J, Moilanen A, Paudel K, Kamali S, Ding K, Cribb W, et al. Quantitative determination of magnetite and maghemite in iron oxide nanoparticles using Mössbauer spectroscopy. SN Appl Sci. 2019;1(12). doi: 10.1007/s42452-019-1699-2

[90] Schwertmann U, Murad E. The Influence of Aluminum on Iron Oxides: XIV. Al-Substituted Magnetite Synthesized at Ambient Temperatures. Clays and clay miner. 1990;38(2):196–202. doi: 10.1346/ccmn.1990.0380211

[91] Popović S, Ristić M, Musić S. Formation of solid solutions in the system Al2O3-Fe2O3. Materials Letters. 1995;23(1–3):139–42. doi: 10.1016/0167-577x(95)00019-4

[92] Nakaishi H, Yabutsuka T, Yao T, Kitao S, Seto M, Chen W-J, et al. Homogeneous solid-solution formation in Fe2O3–Al2O3 system observed by TEM, XAFS, and Mössbauer spectroscopy. Materials Chemistry and Physics. 2023;303:127764. doi: 10.1016/j.matchemphys.2023.127764

[93] Loewenstein W. The distribution of aluminum in the tetrahedra of silicates and aluminates. Am Mineral. 1954;39:92–6.

[94] Benzid K, Timar-Gabor A. Phenomenological model of aluminium-hole ([AlO4/h ]0) defect formation in sedimentary quartz upon room temperature irradiation: electron spin resonance (ESR) study. Radiat Meas. 2020;130:106187. doi: 10.1016/j.radmeas.2019.106187

[95] Prisecaru I, Kent T. Wmoss4 version f – mössbauer spectral analysis software. 2013. http://www.wmoss.org/downloads/WMOSS4F_A4.pdf

[96] Iron(iii) oxide (544884: nanopowder, < 50 nm particle size (bet)). https://www.sigmaaldrich.com/DE/de/product/aldrich/544884

[97] Kuzmann E, Nagy S, Vértes A. Critical review of analytical applications of Mössbauer spectroscopy illustrated by mineralogical and geological examples (IUPAC Technical Report). Pure and Applied Chemistry. 2003;75(6):801–58. doi: 10.1351/pac200375060801

[98] Gräfe M, Donner E, Collins RN, Lombi E. Speciation of metal(loid)s in environmental samples by X-ray absorption spectroscopy: a critical review. Anal Chim Acta. 2014;822:1–22. doi: 10.1016/j.aca.2014.02.044 24725743

[99] Cieslak J, Dubiel SM. Simultaneous analysis of several Mössbauer spectra. Acta Phys Pol A. 2008;114:1691–705. doi: 10.12693/APhysPolA.114.1691

[100] Stevens JG, Khasanov A, Miller JW, Pollak H, Li Z. Mössbauer mineral handbook. 2005. Available: https://www.semanticscholar.org/paper/M%C3%B6ssbauer-mineral-handbook-Stevens-Khasanov/b5be25d2ce47dca9800b2af6ca3b3e1da04d7db0

