# Correction: Can laboratory-based XAFS compete with XRD and Mössbauer spectroscopy as a tool for quantitative species analysis? Critical evaluation using the example of a natural iron ore

**DOI:** 10.1371/journal.pone.0350050

**Published:** 2026-05-26

**Authors:** Sebastian Praetz, Christopher Schlesiger, Damian Alexander Motz, Stephen Klimke, Moritz Jahns, Christine Gottschalk, Lena Heinrich, Eva Maria Heppke, Wolfgang Malzer, Franz Renz, Carla Vogt, Birgit Kanngießer

The sixth sentence of the fourth paragraph in the Introduction should have cited references 25 and 28 instead of 18 and 20.

The correct sentence should read: Nevertheless, these materials can still be examined by small-angle X-ray scattering (SAXS), but SAXS is a special technique with relatively limited accessibility and, moreover, does not provide the same information as regular XRD does and is instead rather used for nanoparticle size or pore size determinations [25, 28].

25. Spieß L, Teichert G, Schwarzer R, Behnken H, Genzel C. Moderne Röntgenbeugung: Röntgendiffraktometrie für Materialwissenschaftler, Physiker und Chemiker. 2., überarb. und erw. Aufl. ed. Vieweg Teubner. 2009.
28. Sharma R, Bisen D, Shukla U, Sharma B. X-ray diffraction: a powerful method of characterizing nanomaterials. Recent Res Sci Technol. 2012.
There is an error in reference 66. The correct reference is: Motz DA. Entwicklung von Referenzmaterialien für die Röntgen-Nahkanten-Absorptionsspektroskopie am Laboraufbau. Hannover: Institutionelles Repositorium der Leibniz Universität Hannover; 2021. https://doi.org/10.15488/10431
