# Supplementary material for: Can laboratory-based XAFS compete with XRD and Mössbauer spectroscopy as a tool for quantitative species analysis? Critical evaluation using the example of a natural iron ore
Source: PLoS One. 2025 May 16;20(5):e0323678. doi: 10.1371/journal.pone.0323678 (PMC12083805; doi:10.1371/journal.pone.0323678)
Supplement: S1 File — See DOI: 10.1039/x0xx00000x. (PDF) [file pone.0323678.s001.pdf]

Supporting Information

Can laboratory-based XAFS compete with XRD and Mössbauer spectroscopy as a tool for quantitative species analysis? Critical evaluation using the example of a natural iron ore

Sebastian Praetz[1¶*], Christopher Schlesiger[1¶], Damian Alexander Motz[2,#a¶*], Stephen Klimke[2], Moritz Jahns[2], Christine Gottschalk[2,#b], Lena Heinrich[3,#c], Eva Maria Heppke[4], Wolfgang Malzer[1], Franz Renz[2], Carla Vogt[2,#d] and Birgit Kanngießer[1]

[1]Technische Universität Berlin, Institute of Optics and Atomic Physics, Berlin, Germany
[2]Leibniz University Hannover, Institute of Inorganic Chemistry, Hannover, Germany
[3]Leibniz Institute of Freshwater Ecology and Inland Fisheries (IGB), Berlin, Germany
[4]Technische Universität Berlin, Institut für Chemie, Berlin, Germany
[#a]Current Address: Leibniz University Hannover, Institute of Sanitary Engineering and Waste Management, Hannover, Germany
[#b]Current Address: Amino GmbH, Frellstedt, Germany
[#c]Current Address: Institute of Environmental Science and Geography, Potsdam, Germany
[#d]Current Address: TU Bergakademie Freiberg, Institute of Analytical Chemistry, Freiberg, Germany
*Corresponding authors: sebastian.praetz@tu-berlin.de, motz@isah.uni-hannover.de
¶: These authors contributed equally to this work.

Table of contents

Characterization of the sample system 2
Macroscopic mineral determination 2
Particle sizes 3
Elemental Analysis 4
Speciation 6
X-ray absorption spectroscopy 8
Sample Preparation 8
Normalized Spectra 8
Edge and pre-edge – Qualitative analysis 8
Linear combination fitting – Model mixtures 9
Linear combination fitting – Mexican iron ore 11
Linear combination fitting with the Mössbauer revealed γ-Fe_2O_3 12
Quantitative X-ray powder diffraction 14
Rietveld refinement – Model mixtures 14
Rietveld refinement – Mexican iron ore 17
Mössbauer spectroscopy 18
Hyperfine parameters – Qualitative analysis 18
Quantitative analysis – Model mixtures 20
Quantitative analysis – Mexican iron ore 22
Compact overview 24
Data availability 24
References 24

Characterization of the sample system

The sample system consisted of the following materials:

1) α-Iron(III) oxide (α-Fe_2O_3): purity ≥ 99%, micro particle powder (d < 5 µm), *Honeywell*.

2) Iron(II, III) oxide (Fe_3O_4): purity 95%, micro particle powder (d < 5 µm), *Sigma Aldrich*.

3) Synthetic model mixtures: three different weight ratios α-Fe_2O_3 / Fe_3O_4 (ω_{rel}(α-Fe_2O_3) / ω_{rel}(Fe_3O_4) = 30 / 70, 50 / 50 and 70 / 30), prepared from 1) and 2) (details see Table S5).

4) Natural iron ore: origin Mexico, purchased from *Mineraliengrosshandel Hausen GmbH*, (inaccurately) labeled as "Mexican magnetite".

Both pure iron oxide samples (α-Fe_2O_3 micro particles and Fe_3O_4 micro particles), purchased for the use as reference substances in the laboratory XAFS measurements, as well as the Mexican iron ore (labelled as "Mexican magnetite") were characterized in detail before they were used in the XAFS, quantitative XRD and Mössbauer spectroscopic measurements. As already mentioned in the article, the pre-characterizations of these substances were also part of the work Motz 2021 [1]. Thus, to get an even more detailed insight into the characterization results, see Motz 2021 [1]. Nevertheless, it must be mentioned, that the results have been reevaluated in course of this study (to get optimal / assured results). This is also the case with the determined Mössbauer hyperfine parameters for the Mexican iron ore presented in Table S12 since in [1] Mössbauer spectroscopy was used as an additional qualitative pre-characterization method for the sample, but just with focus on the hyperfine parameters (without consideration of the subspectra areas and quantitative evaluations).

Macroscopic mineral determination

The purchased Mexican iron ore was examined using various macroscopic mineral determination methods, which are very common in mineralogy / geology to get a first impression and classification of a located mineral or rock sample, before it was further prepared (crushing and milling) and characterized in more detail. The purpose of these preceding examinations of the natural sample was to verify the basic categorization, specified by the retailer, and therefore to check, whether the sample was in general worthwhile regarding further preparations, detailed pre-characterizations and XAFS, quantitative XRD and Mössbauer measurements and whether the sample was suitable to deal with the scientific question of this work. Table S1 presents the obtained results compared to literature values and descriptions of magnetite and hematite. In addition, Fig S1 shows two example pieces of the raw and unprepared sample. It has to be highlighted that some of these macroscopic determination methods (especially gloss and Mohs hardness) actually just apply to minerals, thus, to relatively pure and homogenous natural compounds. As it is described in the following, the sample of this study was not a mineral but rather a rock, which limits the feasibility of these macroscopic determination methods. Nevertheless, all results are presented in Table S1 because these examinations were initially performed under the assumption of a pure magnetite. Furthermore, the obtained results provided the first clues for the sample being such a mineral mixture instead of a pure magnetite.

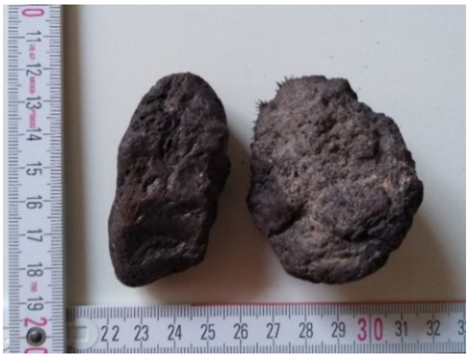

Fig S1 Two examples of the Mexican iron ore ("Mexican magnetite") polycrystalline raw pieces. The scale of the ruler is in centimeters. (taken from Motz 2021[1], picture by Damian Motz, license: *CC BY 3.0 DE*, https://creativecommons.org/licenses/by/3.0/de/)

Table S1 Macroscopic mineral determination of the Mexican iron ore sample. The results are compared to the literature. §§

	Mexican iron ore ("Mexican magnetite")	Magnetite (Fe_3O_4) [2–4]	Hematite (α-Fe_2O_3) [2–4]
**Color**	grey, a few red spots	grey-black	red-brown, grey-black
**Streak**	grey-red §§§ (see Fig S2c)	black	red
**Gloss**	weak, mainly matt	metallic gloss or matt	metallic gloss or matt
**Mohs hardness**	≈ 6.0 – 6.5 §§§§	6.0	6.0
**Magnetism**	ferrimagnetism moderately present	ferrimagnetism present	antiferromagnetism (sometimes ferrimagnetism weakly present)
**Morphology**	polycrystalline aggregates (pieces of a few centimeters in size, see Fig S1)	polycrystalline aggregates or monocrystals (octahedral)	polycrystalline aggregates (sometimes as kidney ore) or tabular monocrystals

§§ Technically speaking, the feasibility of macroscopic mineral determination methods (especially gloss and Mohs hardness) is limited in case of the sample examined in this study since it is not a pure / single mineral but rather a rock. The reasons why these investigations were performed in this study despite this limitation are presented in the text.
§§§ Streak was indirectly determined in course of this work, because the minerals sample was milled in later steps and the streak corresponds to the color of a powdered mineral sample.
§§§§ Mohs hardness was determined using a Swiss army knife (pocketknife). These knifes show a Mohs hardness of 7.0 [2] and thus can be utilized to get a very fast, approximated value. The Mexican iron ore sample was slightly scratchable by the pocketknife. This property corresponds to an approximated Mohs hardness of about 6.0 – 6.5.

One can easily recognize from the literature values and determination in Table S1 that the minerals magnetite and hematite in general have relatively similar macroscopic properties. The most essential differences are the streak (= color of the powdered sample), the magnetic properties and in some cases the morphology and the color. The iron ore sample´s properties, especially the streak (grey-red, see Fig S2c) and a weakened ferrimagnetism (compared to other magnetite samples), implied that the sample actually contained magnetite (as it was specified by the retailer) but also hematite. A possible significant content of goethite (instead of or in addition to hematite), which is α-FeO(OH) and related to hematite, was excluded due to the clear red and not brown proportion of the streak. These findings in combination with the results of the further detailed characterizations demonstrated that the sample was not a pure magnetite, respectively a (relatively) pure mineral, but rather a rock consisting of more than just one iron oxide. These results made the sample an interesting and well suitable sample for the laboratory XAFS experiments and the scientific question (species quantification) presented in this work.

Particle sizes

To check the particle sizes a Digital Microscope *VHX 600* with zoom objective *VH-Z100UR* (*Keyence Corporation*, Osaka, Japan) was used. In each case a spatula tip´s worth of iron(III) oxide, iron(II, III) oxide and the milled Mexican iron ore was prepared on a plane aluminum sample holder pasted with a graphite pad and then examined with the light microscope. The Fig S2a - c show some of the obtained results.

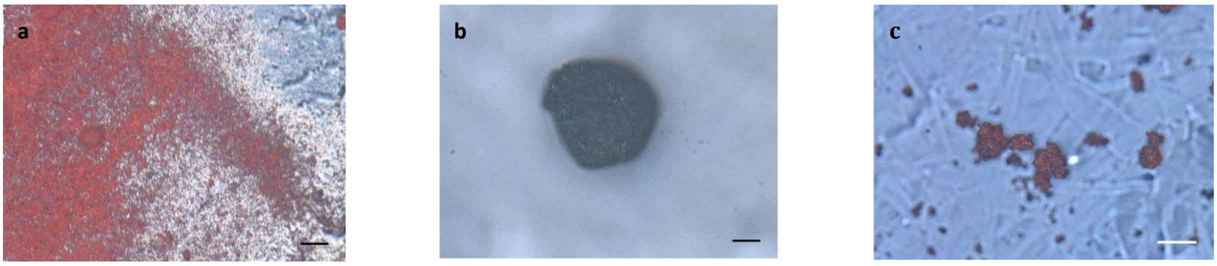

Fig S2 Light microscopic images of the substances – **a** iron(III) oxide (enlargement 1000x, scale bar d = 25 µm), **b** iron(II, III) oxide (enlargement 1000x, scale bar d = 25 µm), **c** Mexican iron ore (enlargement 700x, scale bar d = 50 µm). (taken from Motz 2021 [1], pictures by Damian Motz, license: *CC BY 3.0 DE*, https://creativecommons.org/licenses/by/3.0/de/)

The iron(III) oxide (Fig S2a) turned out as a red fine powder consisted of particles much smaller than 5 µm and was consistent with the supplier's specification (d < 5 µm). So this sample showed a perfect particle size for XAFS due to the rule that particles have to be smaller than one absorption length ($d < \mu^{-1}$) to get optimal results.[5] Consulting the NIST database [6] yields for Fe_2O_3 to the boundary μ^{-1} particle size $d(Fe_2O_3) \approx$ 6.8 µm (at E = 7.14756 keV, thus, slightly above the Fe K-edge). The iron(II, III) oxide (Fig S2b) was composed of grey-black aggregates in the size of 30 up to 200 µm. However, the aggregates themselves were very brittle and consisted of much smaller crystallites in a scale similar to the iron(III) oxide particles (smaller than 5 µm). Therefore, these aggregates could be removed easily by grounding the iron(II, III) oxide slightly in an agate mortar for a few seconds, beforehand the XAFS preparation. The obtained separated smaller crystallites were also in the perfect particle size range for XAFS (d < 5 µm, boundary μ^{-1} particle size based on NIST [6] $d(Fe_3O_4) \approx$ 6.6 µm at E = 7.14756 keV). The crushed and milled Mexican iron ore (Fig S2c) mainly showed particle sizes smaller than 10 - 20 µm (despite a few larger particles). Assuming a composition of iron oxides an exceed of the ideal XAFS particle size (at least in case of a noteworthy portion of the iron ore particles) can be concluded. Hence, slight limiting effects on the XAFS spectra quality had to be considered. Also visible is the already mentioned (see macroscopic mineral determination) red-grey color of the mineral powder instead of black in case of a purer magnetite.

Elemental Analysis

To determine some of the elemental concentrations, especially the iron content, micro X-ray fluorescence analysis (µ-XRF) followed by the more precise inductively coupled plasma optical emission spectroscopy (ICP-OES) was applied.

µ-XRF. An *µProbe EAGLE II* with a rhodium X-ray tube (*EDAX*, Mawah, USA) was used for qualitative and standard less quantitative (based on fundamental parameters) µ-XRF analyses. At first, the three iron oxides were set on a µ-XRF powder sample holder. Then, XRF analysis was done at three different areas of each sample (thus, three replicate / triple determinations) with an acceleration voltage of U = 40 kV, life time t = 250 s, death time limit t = 30%, 5000 cps and a spot size d = 50 µm. Following the qualitative analysis, a standard less quantification (fundamental parameter based quantification) to approximate the element concentrations was performed. Table S2 shows the results which were obtained as mean values of the three-point analyses. The element concentrations are referred to the respective (most stable) element oxide due to the fact that oxygen is not accessible with XRF analysis and therefore a fundamental parameter based quantification without consideration of the expected sample´s high oxygen contents would have been more incorrect.

Table S2 Standardless µ-XRF elemental quantification results referred to the respective element oxides. The mean values and the belonging uncertainties (in the form of the standard deviations) of the three-point analyses are shown.

Sample	$\omega_{rel}(Fe_2O_3)$ / %	$\omega_{rel}(MnO)$ / %	$\omega_{rel}(Al_2O_3)$ / %	$\omega_{rel}(SiO_2)$ / %
**Iron(III) oxide micro particles**	99.50 ± 0.01	0.45 ± 0.01	Not detected	Not detected
**Iron(II, III) oxide micro particles**	97.23 ± 0.42	0.58 ± 0.01	2.17 ± 0.41	Not detected
**Mexican iron ore**	75.42 ± 13.81	Not detected	5.87 ± 2.94	18.15 ± 10.45

The µ-XRF results of the iron(III) and iron(II, III) oxide micro particles revealed that the iron oxide contents are in good accord to the manufacturer information. Especially in case of the iron(III) oxide micro particles the obtained value of the iron oxide content $\omega_{rel}(Fe_2O_3)$ = 99.50% ± 0.01% could be compared to the manufacturer information (≥ 99%) directly. The iron ore from Mexico also showed high contents of aluminum oxide and silicon oxide due to the fact it is a mineral sample, which have contents of silicates and aluminosilicates frequently. In addition to the results shown in Table S2 small amounts of calcium oxide have also been detected in the ore sample, which is also explainable by paragenesis and therefore accompanying minerals.

ICP-OES. For more precise element quantification ICP-OES was used. These quantitative analyses were done with an ICP-OES *Spectro ARCOS* (*Spectro Analytical Instruments GmbH*, Kleve, Germany).

Firstly, the three iron substances were dissolved by a microwave assisted digestion (using the microwave assisted digestion system *µPREP-A*, *MLS GmbH*, Leutkirch, Germany) in aqua regia (HCl : HNO_3 = 3 : 1). The used acids were p. a. sub boiled nitric acid ($\omega_{rel}(HNO_3)$ = 65%) and p. a. hydrochloric acid (ω_{rel}(HCl) = 32%). Three samples of each iron compound were taken and dissolved (three replicate / triple determinations). To take possible contaminations of the applied microwave tubes and acids into account blank digestions were also carried out. Subsequently the total digested samples were diluted with pure water for the further ICP-OES analyses. In case of the Mexican iron ore a removal of small amounts of insoluble residues (silicates) using metal free syringe filters (*VWR*, Radnor, USA) was necessary before dilution (in this case the blank digestions were also filtered the same way). The quantifications of several element concentrations were performed by external calibrations with multi element standard solutions. These were prepared of commercial stock solutions (single element stock solutions, β = 1000 mg/L, *Merck*, or a multi element stock solution, *Multi IV*, β = 1000 mg/L, *Merck*) which were diluted with pure water but were also mixed with nitric acid and hydrochloric acid to get a matrix similar to the diluted samples. Especially the iron contents were quantified since these values allowed important information on the identity and purity of the three substances. This was particularly required in case of the XAFS reference substances iron(III) and iron(II, III) oxide micro particles. Furthermore, precise iron contents were important to estimate the sample amounts for the XAFS preparations more precisely to get optimal (or at least usable) area densities. The choice of other quantified elements was oriented towards the µ-XRF results (manganese in case of all samples, calcium just in case of the ore) and general chemical and physical properties similar to iron (cobalt and nickel as part of the iron triad, aluminum and chromium due to their charge densities in oxidation state +III which are similar to iron(III)) because of possible indirect influences on the samples X-ray absorption properties (due to mixture of structural similar substances or even solid solutions). In case of the iron(III) and iron(II, III) oxide micro particles the iron quantification was done by a 9 point calibration (ω(Fe) = 9 – 160 mg/kg) and the quantifications of aluminum, chromium, manganese, cobalt and nickel by 5 point calibrations (ω(Al) = 30 – 300 µg/kg, ω(Mn) = 8 – 90 µg/kg, ω(Cr, Ni, Co) = 3 – 30 µg/kg). The analyses of the iron ore sample´s contents of iron, aluminum, chromium, manganese, cobalt, nickel and calcium were all done by 6 point calibrations (ω(Fe) = 5 – 80 mg/kg, ω(Al, Cr, Mn, Co, Ni, Ca) = 40 – 2000 µg/kg). In all cases various lines of each element were measured in which the lines selection based on several aspects such as predicted / observed sensitivities and interferences. The evaluations of each element´s measurement results were done in accordance with DIN 38402-51:1986 [7] and DIN 32645:2008 [8]. Then these results of the different element lines were averaged, after they had been checked for outliers with Grubbs test. Finally, the determined elemental contents of the three replicates of each iron compound were also checked for outliers via Grubbs test and averaged to get the overall results.

Table S3 shows the overall results of iron analyses compared to theoretical values as well as manufacturer information and Table S4 presents the results of other elements.

Table S3 ICP-OES iron quantification results as the percentage of mass of iron and of the respective iron oxide Fe_2O_3 and Fe_3O_4 (assuming that all of the determined iron contents is bound in the respective iron oxide) compared to theoretical values/ manufacturer information. The belonging error values were obtained by the application of uncertainty propagation to evaluated confidence intervals. In case of the iron ore sample no conversion of the determined iron content to a specific iron oxide has been done, because it was a mixture of both oxides (see later analyses).

Sample	ω_{rel}(Fe) / %	ω_{rel}(iron oxide) / %	ω_{rel}(Fe) theor. / %	ω_{rel}(iron oxide) theor. / %
**Iron(III) oxide micro particles**	68.16 ± 1.34	97.45 ± 1.92	pure: 69.95<br>manufacturer: 69.2 – 71.3 [9]	manufacturer: ≥ 99 [9]
**Iron(II, III) oxide micro particles**	68.25 ± 1.37	94.32 ± 1.89	pure: 72.36<br>manufacturer: 68.0 – 76.7 [10]	manufacturer: 95 [10]
**Mexican iron ore**	63.05 ± 1.45	-	very pure magnetites:<br>up to ≈ 72% [3] – 72.4% [4]<br>very pure hematites:<br>up to ≈ 70% [4]	-

Table S4 ICP-OES quantification results of selected secondary and trace elements. The belonging error values were obtained by the application of uncertainty propagation to evaluated confidence intervals. In case of the iron(III) oxide and iron(II, III) oxide micro particles the calcium content has not been examined.

Sample	*ω*(Al) / mg/kg	*ω*(Ca) / mg/kg	*ω*(Co) / mg/kg	*ω*(Cr) / mg/kg	*ω*(Mn) / mg/kg	*ω*(Ni) / mg/kg
**Iron(III) oxide micro particles**	< LOD	-	21.48 ± 2.01	< LOD	1769.7 ± 32.2	60.31 ± 7.11
**Iron(II, III) oxide micro particles**	2515.7 ± 68.5	-	16.61 ± 2.10	< LOD	2186.8 ± 41.4	26.94 ± 7.81
**Mexican iron ore**	1860 ± 355	4288 ± 132	< LOD	< LOD	Detected (< LOQ)	Detected (< LOQ)

The resulting iron concentrations of the two reference substances were compared with the manufacturer specifications for iron oxide concentration and purity. As was already the case with the µ-XRF analyses, considering the obtained uncertainties the values fitted to these specifications well. However, it has to be pointed out that the iron(II, III) oxide micro particles had relatively high contents of manganese and especially aluminum – the analyzed aluminum content is even higher than the iron ore´s content of the light metal. Possibly, due to matrix effects, this must be taken into account when evaluating the use of the iron(II, III) oxide as reference substance in the XAFS measurements. The iron mass percentage of the Mexican iron ore was lower compared to possible contents of very pure minerals. Nevertheless, this is explainable by several impurities of the magnetite / hematite as well as accompanying minerals (e.g. containing silicon and calcium such as several silicates and aluminosilicates) as it was the case with the XRF analyses results. In addition to this, the natural ore was also checked for possible contents of rare earth elements (REE) because some of the L-lines of REEs (e.g. L-edges of Eu, Gd and Tb in the energy range of 6.9 keV to 7.5 keV, for example Eu L1, Gd L3) are in the same energy range like the iron K-line [5] (direct influence on the absorption properties, inferences) which was in focus of our work. To this end, the cerium and lanthanum contents, representative of all the REE's, were analyzed semi-quantitatively (standard solution ω(Ce, La) = 100 µg/kg prepared of Ce and La stock solutions, β = 1000 mg/L, *Carl Roth*). As a result, no significant REE contents were determined in the ore sample and therefore REE interferences in the XAFS spectra could be excluded.

Speciation

For qualitative chemical speciation X-ray powder diffraction (XRD) was applied. The XRD analyses were performed in reflection setup at a *D4 Endeavor* with a graphite monochromator (*Bruker Corporation*, Billerica, USA) (iron(III) oxide micro particles and iron(II, III) oxide micro particles) and at a *Stadi P* with PSD detector (*STOE & Cie GmbH*, Darmstadt, Germany) (Mexican iron ore). In both cases, Cu Kα radiation was used. The evaluations were conducted by the use of the software *WinXPow* (Version 1.08, *STOE & Cie GmbH*, Darmstadt, Germany). [11]

Fig S3a – c show the obtained diffraction patterns with qualitative phase analyses accomplished. It is evident that the XRD patterns of iron(III) micro particles (Fig S3a) as well as iron(II, III) oxide micro particles (Fig S3b) can be explained by the respective expected iron oxide species and phase (α-Fe_2O_3 and Fe_3O_4) entirely. In case of the natural ore sample (Fig S3c), besides a typical accompanying silicate mineral quartz (SiO_2), both iron oxide species (α-Fe_2O_3 and Fe_3O_4) were found in the XRD pattern. Thus, already this first qualitative XRD measurement (just like the observed grey-red color of the streak and the milled sample instead of a pure black shade) of the Mexican iron ore demonstrated that the sample mainly consisted of the two iron oxide minerals magnetite and hematite (paragenesis) and, therefore, that it was not a pure magnetite containing iron mineral. This is explainable by the fact that magnetite can be transferred to hematite (or Fe(III) species such as maghemite, limonite, etc.) due to possible secondary mineral formation processes weathering (in this case oxidation), metamorphism and especially pseudomorph formation (martitization).[4,12–14] At a first glance, the reasonable presence of very small amounts of an additional iron phase in form of iron(III) oxide-hydroxide (α-FeO(OH)), thus, goethite was assumed (due to the very small reflex at 2θ = 21.2°, close to the 20.860° quartz signal, see Fig S3c), but this could not be unequivocally confirmed by the later (quantitative) XAFS, XRD or Mössbauer examinations.

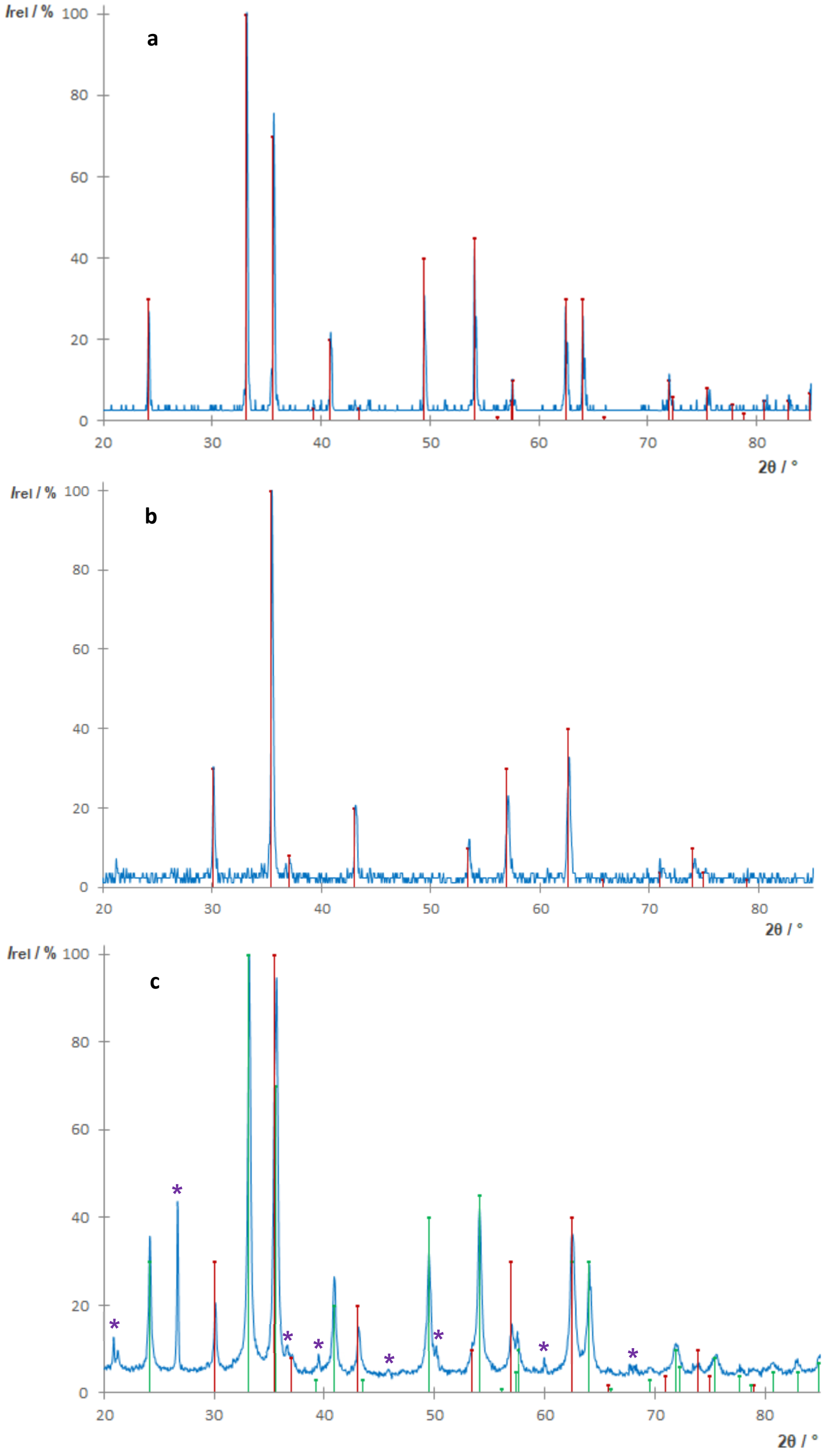

Fig S3 X-ray powder diffraction patterns for qualitative speciation – **a** Diffractogram of the iron(III) oxide micro particles (blue) compared to an α-Fe_2O_3 reference diffractogram (red, PDF [33-664]), **b** diffractogram of the iron(II, III) oxide micro particles (blue) compared to an Fe_3O_4 reference diffractogram (red, PDF [19-629]), **c** diffractogram of the Mexican iron ore (blue) compared to α-Fe_2O_3 (green, PDF [33-664]) and Fe_3O_4 (red, PDF [19-629]) reference diffractograms. The extra signals in the ore diffractogram, especially those at 2θ = 20.860°, 26.640°, 59.960° and about 68°, can be explained by the accompanying mineral quartz. The positions of the most essential reflexes of an SiO_2 reference diffractogram (purple asterisks, PDF [46-1045]) are marked. (taken from Motz 2021 [1], c has been deviated, original pictures by Damian Motz, license: *CC BY 3.0 DE*, https://creativecommons.org/licenses/by/3.0/de/)

X-ray absorption spectroscopy

Sample Preparation

The sample powder was applied between two tape layers, sliced, and analyzed via XRF to estimate the iron mass deposition. The required absorption thickness was achieved by stacking up to 20–40 slices, depending on particle size and analyte concentration. For wax pellet preparation, the powder was mixed with Hoechst Wax C© (e.g., 1:6 for pure iron oxide) in a vortex shaker with glass balls to ensure stability and minimize absorption. The mixed material was then pressed with a hydraulic press.
A detailed description of the sample preparation can be found in the main paper.

Normalized Spectra

Fig S4 shows the normalized XAFS spectra with the *ATHENA* software [15] and its flattened algorithm (a) and normalized with the *Larch* software package [16] and its unflattend algorithm (b). The raw data files of the XAFS measurements are available at the Zenodo repository.[17]

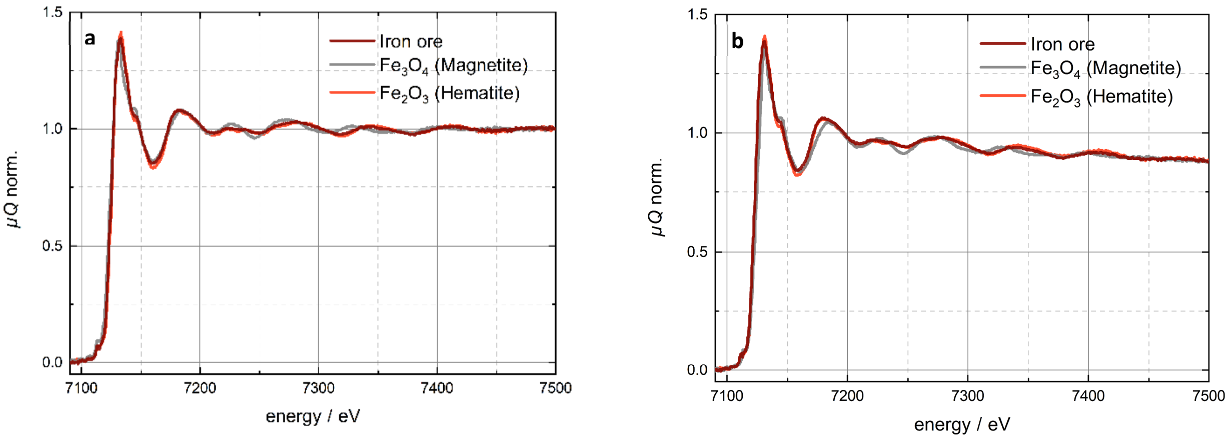

Fig S4 XAFS-spectra of the Mexican iron ore and the references α-Fe_2O_3 and Fe_3O_4. **a** normalized with *ATHENA* (flattened algorithm) and for comparison **b** unflattend normalization with *Larch*. Measurement time was 10 h per spectrum. The measurements were performed at room temperature.

Edge and pre-edge – Qualitative analysis

Beforehand the quantitative XAFS evaluation, an initial qualitative analysis with focus on the XANES area including the pre-edges was performed. Fig S5 shows a closer look on the XANES region of Mexican iron ore and the two references α-Fe_2O_3 and Fe_3O_4 with an inlet of the pre-edges. The qualitative analysis is described in the main paper.

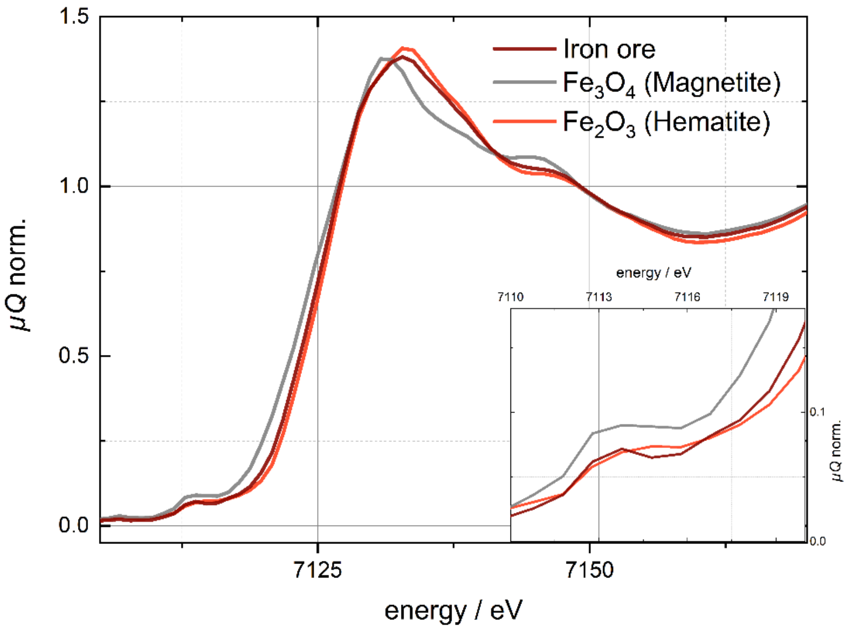

Fig S5 XANES-region around the edge of the Mexican iron ore and the references α-Fe_2O_3 and Fe_3O_4 and inlet with the close up on the pre-edges.

Linear combination fitting – Model mixtures

The raw data files of the lab-XAFS, XRD and Mössbauer measurements are available at the Zenodo repository.[17] The species α-Fe_2O_3 and Fe_3O_4 have very similar XAFS spectra with only minor differences in the XANES and EXAFS region, especially when comparing them to a pure metal foil. In previous studies as described in Schlesiger et al. 2015 [18] the capability of quantification with Lab-XAFS on mixtures consisting of pure iron powder and α-Fe_2O_3 could already be shown, with a deviation to the weight-in ratio for 30 minutes measurements in a range of 0 to 2 percent points (p.p.).

Table S5 Ideal and actual achieved weight-in and mass percentage ratios of the prepared α-Fe_2O_3 and Fe_3O_4 model mixtures.

Target mass percentage ratios $\omega_{rel}(Fe_2O_3)$ / $\omega_{rel}(Fe_3O_4)$	Target weight-in ratios $m(Fe_2O_3)$ / $m(Fe_3O_4)$ (assuming pure species)	Actual achieved weight-in ratios $m(Fe_2O_3)$ / $m(Fe_3O_4)$	Actual achieved mass percentage ratios $\omega_{rel}(Fe_2O_3)$ / $\omega_{rel}(Fe_3O_4)$
30 / 70	0.3 g / 0.7 g	0.3180 g / 0.7059 g	assuming pure species: 31.1 / 68.9<br>considering the determined purities: (30.3 ± 0.6) / (65.0 ± 1.3)<br>considering the purities and using just the contributions of the both iron species to the total mass: 31.8 / 68.2
50 / 50	0.5 g / 0.5 g	0.5140 g / 0.5174 g	assuming pure species: 49.8 / 50.2<br>considering the determined purities: (48.6 ± 1.0) / (47.3 ± 1.0)<br>Considering the purities and using just the contributions of the both iron species to the total mass: 50.6 / 49.4
70 / 30	0.7 g / 0.3 g	0.7037 g / 0.3041 g	assuming pure species: 69.8 / 30.2<br>considering the determined purities: (68.0 ± 1.3) / (28.5 ± 0.6)<br>considering the purities and using just the contributions of the both iron species to the total mass: 70.5 / 29.5

The spectra and the results of the LCF are presented in Fig S6 and S7. The quantitative results from the fit are listed in Table S6. It has to be emphasized, that the calculated results, which relate to the ratio r_i of the bound iron in each species, were converted to weight percentages of the species for comparability with the other two methods. To determine this, we calculate the number of “molecules” (more precisely formula units since the substances of this study are mostly ionic compounds or something between ionic compounds and covalent networks) each species N_i i.e., r_i divided by the number of said element in one “molecule” (formula unit) of the respective species, and multiply it by the molar mass of that species M_i. Equation (S1) presents the calculation.

$$\omega_{rel} = N_i \cdot M_i \,/\, \sum_i N_i \cdot M_i \qquad \text{(S1)}$$

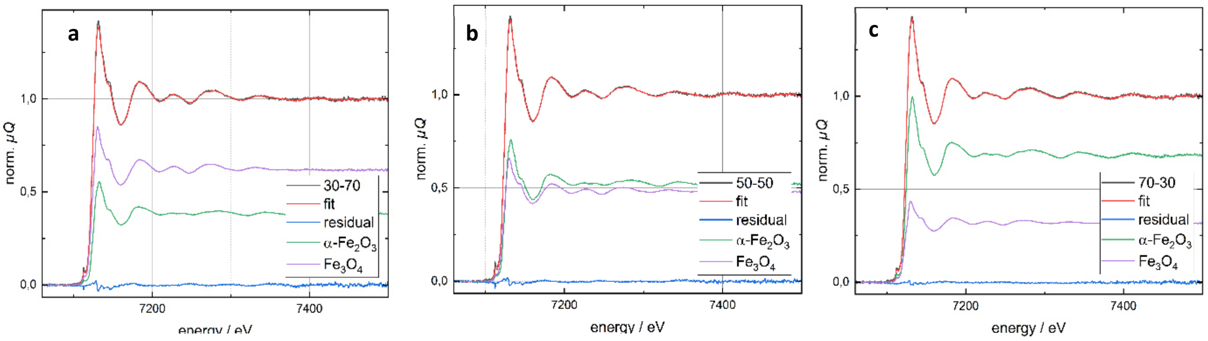

Fig S6 LCF results of the three synthetic α-Fe_2O_3 / Fe_3O_4 mixtures prepared by applying the sample/mixtures on adhesive tape. **a** with a ratio of 30/70 of α-Fe_2O_3 / Fe_3O_4. **b** with a ratio of 50/50 and **c** with a ratio of 70/30. For the actual achieved ratio of the mixtures see Table S5 or S6. The quantitative results for the species ratios are listed in Table S6. The measurement time was 10 h per spectrum. The measurements were performed at room temperature.

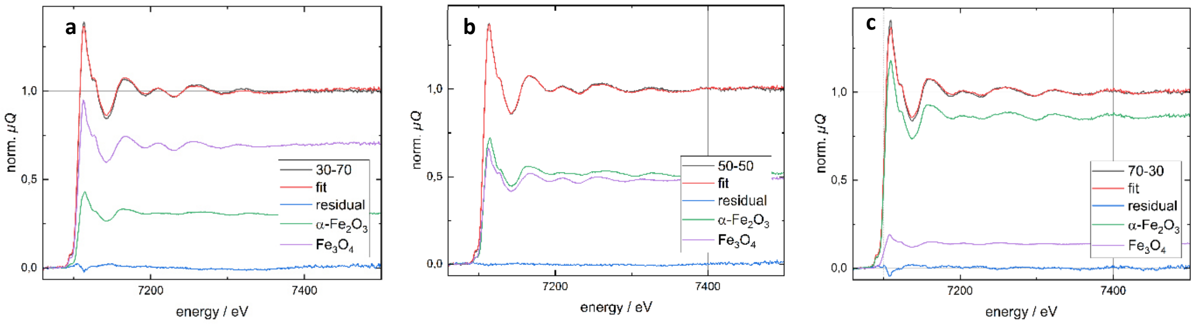

Fig S7 LCF results of the three synthetic α-Fe_2O_3 / Fe_3O_4 mixtures prepared by mixing the sample/mixtures with *Hoechst wax C©* in a ratio of 1:6 and pressing the sample-wax mixtures after vortex shaking into a pellet with a 13 mm diameter. **a** with a ratio of 30/70 of α-Fe_2O_3 / Fe_3O_4, **b** with a ratio of 50/50 and **c** with a ratio of 70/30. For the actual achieved ratio of the mixtures see Table S5 or S6. The quantitative results for the species ratios are listed in Table S6. The measurement time was 2.5 h per spectrum. The measurements were performed at room temperature.

The quantitative results of the mixtures prepared on adhesive tape, which were measured together with the Mexican iron ore, deviate from the actual weight-in ratio between 4.9 and 6.6 p.p. (percentage points). Since the Mexican iron ore was prepared with the same method and the statistical uncertainty for the measurement of the spectra is comparable, we assume, that the uncertainty for this analysis is below 7 p.p. accordingly.

In Table S6, which summarizes the results, the weight-in fractions achieved with the LCF over the entire energy range are included, as this setting was used for the analysis of the Mexican iron ore.

Table S6 Quantitative Lab-XAFS results of the synthetic α-Fe_2O_3 / Fe_3O_4 mixtures using LCF in comparison with the mass percentage ratio of the weight-in model mixtures.

		**tape preparation**			**pellet preparation**		
**Ideal ratio Fe_2O_3 / Fe_3O_4**	**Achieved ratio Fe_2O_3/Fe_3O_4**	**Fe_2O_3**	**Fe_3O_4**	**deviation in p. p.**	**Fe_2O_3**	**Fe_3O_4**	**deviation in p. p.**
30/70	31.8/68.2	25.4 ± 0.6	74.7 ± 0.6	6.4	33.0 ± 2.7	67.0 ± 3.5	1.2
50/50	50.6/49.4	44.0 ± 0.6	56.0 ± 0.6	6.6	53.0 ± 1.6	47.0 ± 2.8	2.4
70/30	70.5/29.5	65.6 ± 0.6	34.4 ± 0.6	4.9	87.0 ± 2.9	13.0 ± 2.9	16.5

Linear combination fitting – Mexican iron ore

The linear combination fitting (LCF) has been performed on the unflattened normalized spectra and the flattened normalized spectra. Fig S8a shows the LCF of the inflated normalized spectra by the Larch software,[12] while Fig S8b shows the results of the in-house LCF algorithm [18] on the unflattened normalized spectra. The quantitative results of the iron species are listed in Table S7 for the different algorithms and normalization procedures. Analogous to the model mixture examinations, the LCF results have been converted to mass percentages using equation (S1).

To compare the actual quality of the LCF of the different fitting approaches by open source and in-house software χ^2 and reduced χ^2_ν have been calculated by equation (S2):

$$\chi^2_\nu = \frac{1}{N}\sum_{\mathrm{i}=1}^{\mathrm{N}} \frac{(\mathrm{data_i} - \mathrm{fit_i})^2}{\varepsilon_\mathrm{i}^2}. \qquad \text{(S2)}$$

Here *N* is the number of data points used for the LCF i.e., the energies of the spectrum, and ε_i describes the measurement uncertainty which is usually unknown to the software and thus set to 1 by default.

Since this spectrometer is using a photon counting detector unit the measurement uncertainty can be estimated by using the photon statistic in the acquired spectra by equation (S3)

$$\varepsilon_\mathrm{i} = \sqrt{\frac{1}{N_\mathrm{i}} + \frac{1}{N_{\mathrm{i},0}}}. \qquad \text{(S3)}$$

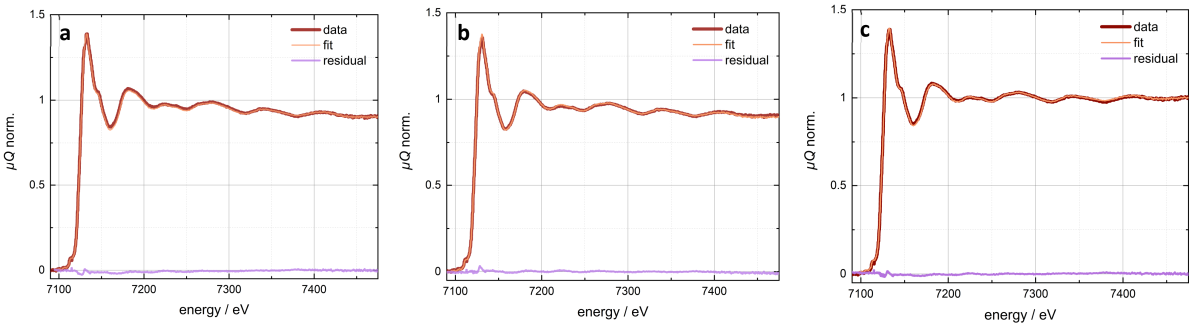

Fig S8 LCF results of the Mexican iron ore. **a** is the LCF performed by the *Larch* software on the unflattened normalized spectra. **b** is the LCF result of the in-house algorithm on unflattened normalized spectra. **c** is the LCF result of the in-house algorithm on the flattened normalized spectra.

N_i and $N_{\mathrm{i},0}$ are the number of photons corresponding to a given energy interval in the measurement with and without the sample, respectively. A good linear combination fit should have a χ^2_ν of 1.[19] Values of χ^2_ν much larger than 1 may indicate poor measurements or incorrect assignments of uncertainties.[20] By considering the value of the χ^2_ν of all fits both in-house LCFs for flattened and unflattened normalized spectra achieved the best fit to approximate the data, even though all applied LCFs overall showed good results.

Table S7 LCF results of the Mexican iron ore of the different software methods. §§

Method	α-Fe_2O_3 in %	Fe_3O_4 in %	χ_1^2	χ_ε^2
ATHENA	71.9 ± 0.5	28.1 ± 0.5	0.0000271	2.676
Larch	71.6 ± 0.6	28.4 ± 0.6	0.0000645	6.693
**In-house flat**	74.3 ± 0.6	25.7 ± 0.6	0.0000225	2.281
**In-house norm**	69.2 ± 0.8	30.8 ± 0.8	0.0000255	1.700
**Ø mean**	71.8 ± 2.6 §§§	28.2 ± 2.6 §§§		

§§ Applied on the normalized spectra. Besides the results of the different LCF methods the manually calculated reduced chi squared without (χ_1^2 ε set to 1) and with (χ_ε^2) considering the approximated measurement uncertainty is displayed. The uncertainty of the species composition results from the standard deviation of the LCF. For more information on the methods we advise for *ATHENA* to [15], for *Larch* to [16] and for the in-house Alg. to [18].

§§§ The uncertainty of the mean value Ø is given by the span of the values.

Linear combination fitting with the Mössbauer revealed γ-Fe_2O_3

Additional measurements and LCF were carried out on the Mexican iron ore including the third reference material γ-Fe_2O_3, since the presence of this phase in the ore was later discovered by Mössbauer spectroscopy. That was completed after the comprehensive characterization of sample and reference material, along with initial XRD and XAFS measurements and the subsequent quantitative analysis. The γ-Fe_2O_3 was retrieved by *Sigma-Aldrich* [21] and has been undergone equal extensive pre-characterization (see Motz 2021 [1]) as the references α-Fe_2O_3 and Fe_3O_4. The lab-XAFS measurement of this three references can be seen in Fig S9a and were compared with synchrotron radiation (SR) XAFS spectra (see Fig S9b) from the sshade.eu [22] data base.[23–25] Despite the great similarity of γ-Fe_2O_3 and Fe_3O_4, especially in the EXAFS regime, distinct differences can be observed in the XANES region specifically shown in the inlets of Fig S9. The resolving power of the lab spectrometer is sufficiently high to display disparities within the edge region, at the white line around 7133 eV and the following shoulder of the white line at 7140 eV. The LCF results of the iron ore measurement is presented in Fig S10a with only α-Fe_2O_3 and Fe_3O_4 as reference standard and with all three references as standard in Fig S10b. For only using the two α-Fe_2O_3 and Fe_3O_4 standard as in the prior measurements and analysis, the determined composition 69% ± 4% α-Fe_2O_3 and 31% ± 3% Fe_3O_4 is in accordance with the prior results in Table S7. By using γ-Fe_2O_3 as an additional standard the resulting composition of the Mexican iron ore is 27% ± 3% γ-Fe_2O_3, 56% ± 3% α-Fe_2O_3 and 17% ± 5% Fe_3O_4. While the residual in Fig S10b seems to be slightly higher than for the two standards LCF, the reduced chi squared, given by the *ATHENA* software,[15] is slightly lower with χ^2 = 0.000108 for the three standards in comparison with χ^2 = 0.000139 for the two standards LCF. This indicates a better matching fit when using all three standards. While the LCF given weight of the α-Fe_2O_3 component deviates 7 percent points (p.p.) from the Mössbauer results (see Table 2), the γ-Fe_2O_3 and Fe_3O_4 given by the LCF deviates of more than 11 p.p. from the Mössbauer results. The deviation of the 3 standard LCF-XAFS results from the Mössbauer results can have various reasons. The Mössbauer results are based on assumptions since the γ-Fe_2O_3 could not be measured. Furthermore, for a correct determination of relative concentrations the spectrometer has to be well calibrated or all the hyperfine parameters have to be known and even then the relative error will be about 5 %.[26]

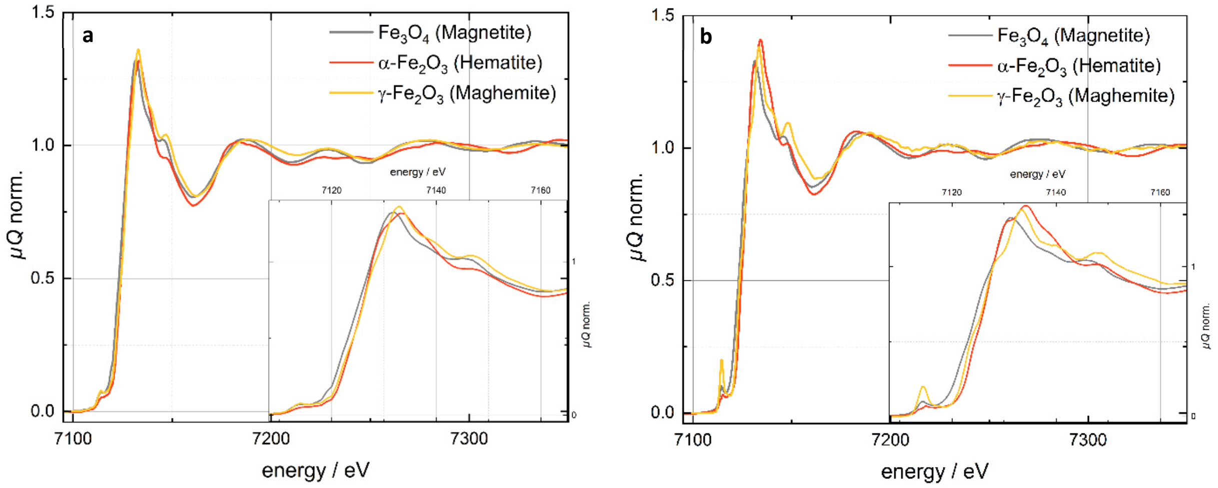

Fig S9 Normalized XAFS-spectra of the reference materials α-Fe_2O_3, γ-Fe_2O_3 and Fe_3O_4. **a** measured with the laboratory XAFS spectrometer and in comparison, with **b** spectra from the sshade.eu[30] SR-data base data base.[31–33] The inlet shows a close up of the XANES and shows the distinguishability of the γ-Fe_2O_3 and Fe_3O_4 species and the capability of the Lab spectrometer (**a**) to differentiate between these.

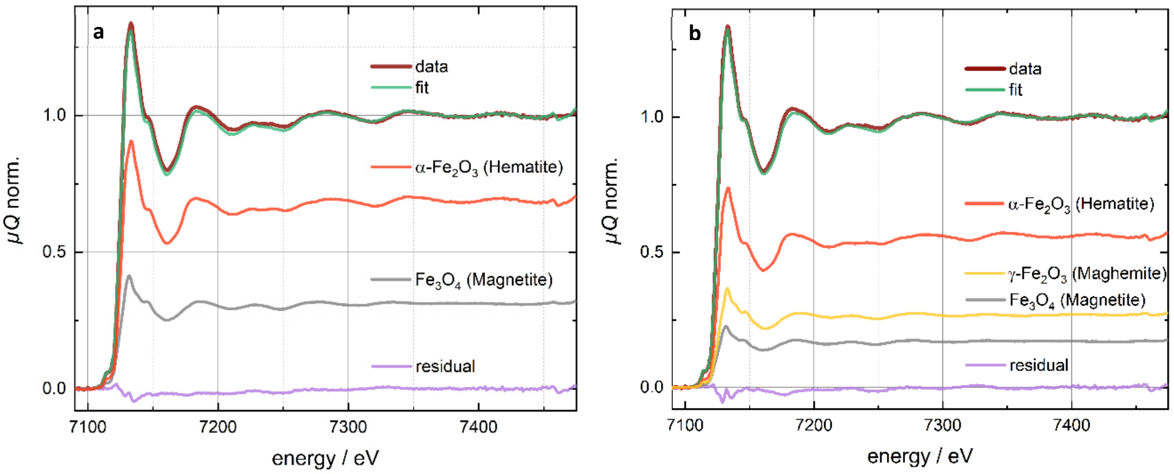

Fig S10 LCF results of the Mexican iron ore **a** with the two reference materials α-Fe_2O_3, and Fe_3O_4 as in Fig. S6 and **b** with additionally γ-Fe_2O_3 as a third reference. In addition to the LCF and the spectrum of the Mexican iron ore the residual and the weight components are displayed. The measurements were performed at room temperature.

Quantitative X-ray powder diffraction

Rietveld refinement – Model mixtures

For the X-ray diffraction measurements of the α-Fe_2O_3/Fe_3O_4 mixtures and the pure references *a Panalytical X'Pert PRO diffractometer* with a Bragg-Brentano setup was used. The Rietveld refinement was done with *Fullprof*.[27,28] We refer to the main paper for more experimental details.

The X-ray diffraction pattern with the results from the Rietveld refinement are shown in Fig S11-13. The quantitative results of the Rietveld refinement are listed in Table S8 alongside the actual weight-in achieved ratio of the two species. The detailed results of the refinement are listed in Table S9. The raw data files of the XRD measurements are available at the Zenodo repository.[17]

Table S8 Quantitative XRD results of the synthetic α-Fe_2O_3 / Fe_3O_4 mixtures using Rietveld Refinement in comparison with the mass percentage ratio of the weight-in model mixtures. §§

Ideal ratio Fe_2O_3 / Fe_3O_4	Achieved ratio Fe_2O_3/Fe_3O_4	Fe_2O_3	Fe_3O_4	deviation in p. p.
30/70	31.8/68.2	32.0 ± 1.2	68.0 ± 1.5	0.2
50/50	50.6/49.4	50.8 ± 1.8	49.2 ± 1.8	0.2
70/30	70.5/29.5	67.9 ± 2.1	32.1 ± 1.8	2.6

§§ by the Rietveld refinement with the software *Fullprof*. . The uncertainty of the mixture composition is given by 3 times e.s.d. according to ASTM E117-13.[31]

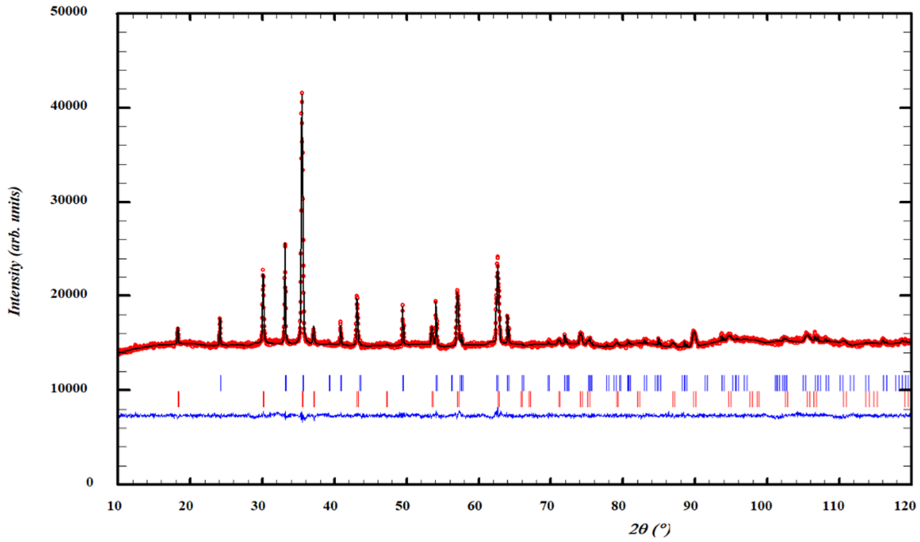

Fig S11 X-ray diffraction pattern of sample α-Fe_2O_3/Fe_3O_4 30/70 with the results of the Rietveld refinement (red: measured; black: calculated; blue: measured–calculated). The vertical bars indicate the reflection positions of Fe_2O_3 (top, blue colored) and Fe_3O_4 (bottom, red colored). The measurement was performed at room temperature.

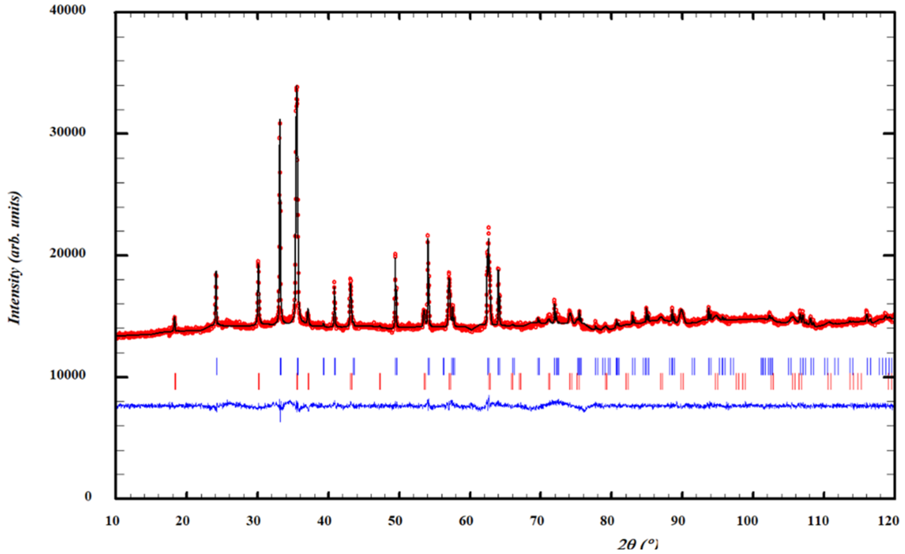

Fig S12 X-ray diffraction pattern of sample α-Fe_2O_3/Fe_3O_4 50/50 with the results of the Rietveld refinement (red: measured; black: calculated; blue: measured–calculated). The vertical bars indicate the reflection positions of Fe_2O_3 (top, blue colored) and Fe_3O_4 (bottom, red colored). The measurement was performed at room temperature.

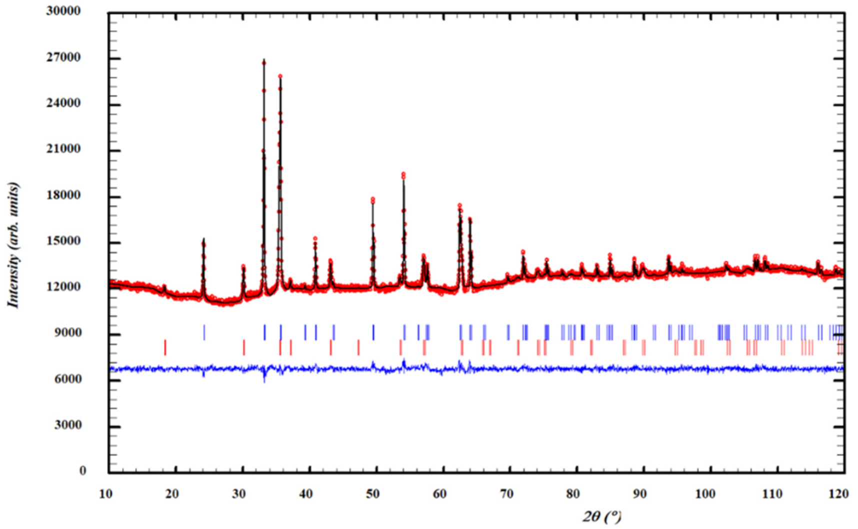

Fig S13 X-ray diffraction pattern of sample α-Fe_2O_3/Fe_3O_4 70/30 with the results of the Rietveld refinement (red: measured; black: calculated; blue: measured–calculated). The vertical bars indicate the reflection positions of Fe_2O_3 (top, blue colored) and Fe_3O_4 (bottom, red colored). The measurement was performed at room temperature.

Table S9 Detailed results of the Rietveld refinements (standard deviations in parentheses).

sample	30-70		50-50		70-30	
Phases	Fe_2O_3	Fe_3O_4	Fe_2O_3	Fe_3O_4	Fe_2O_3	Fe_3O_4
Ratio	32.0(4)	68.0(5)	50.8(6)	49.2(6)	67.9(7)	32.1(6)
Structure type	corundum	spinel	corundum	spinel	corundum	spinel
Space group	$R\bar{3}c$	$Fd\bar{3}m$	$R\bar{3}c$	$Fd\bar{3}m$	$R\bar{3}c$	$Fd\bar{3}m$
Crystal system	trigonal	cubic	trigonal	cubic	trigonal	cubic
Z	6	8	6	8	6	8
a, Å	5.0344(3)	8.3812(5)	5.0344(3)	8.3813(5)	5.0345(2)	8.3833(5)
c, Å	13.7468(9)		13.7461(7)		13.7475(6)	
V, Å^3	301.74(3)	588.73(7)	301.72(3)	588.76(6)	301.77(2)	589.18(6)
Calcu-lated den-sity, g/cm^3	5.273	5.224	5.274	5.225	5.273	5.220
Diffrac-tometer	PANalytical X'Pert MDP Pro		PANalytical X'Pert MDP Pro		PANalytical X'Pert MDP Pro	
Radiation	CuK_α radiation		CuK_α radiation		CuK_α radiation	
Wave-length, Å	λ_1 = 1.54056, λ_2 = 1.54439		λ_1 = 1.54056, λ_2 = 1.54439		λ_1 = 1.54056, λ_2 = 1.54439	
R_p	0.0063		0.00729		0.00689	
R_{wp}	0.00818		0.0095		0.00904	
R_{exp}	0.0081		0.0082		0.0088	
R_{Bragg}	0.132	0.0738	0.170	0.138	0.127	0.155
S	1.02		1.15		1.03	

Rietveld refinement – Mexican iron ore

Quantitative XRD on the Mexican iron ore has been performed with two different software *Topas V6* and *Profex 4.2.4/BGMN*. [29,30,32] Both software show minor differences in the outcomes, see results in Table S10. The XRD patterns as well as the result of the quantitative Rietveld refinements [27] are displayed in Fig S14 for both software. The raw data file of the XRD measurement is available at the Zenodo repository.[17]

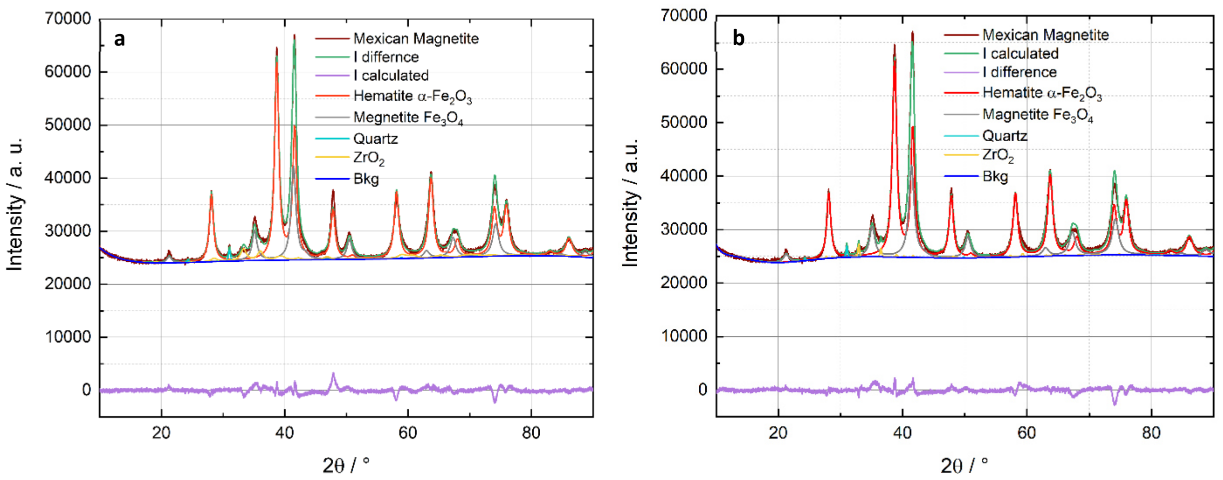

Fig S14 XRD patterns and Rietveld refinement results performed with the software **a** *Topas V6* and **b** *Profex 4.3.4 BGMN*. The total measurement time was 8 h. The measurement was performed at room temperature.

Table S10 Quantitative XRD results of the Mexican iron ore. §§

Ore composition	*Topas V6* (*Bruker*)	*Profex 4.2.4* (*BGMN*)
Magnetite (Fe_3O_4)	26 ± 1	26 ± 1
Hematite (α-Fe_2O_3)	70 ± 1	72 ± 1
Quartz (α-SiO_2)	1 ± 1	1 ± 1
ZrO_2	3 ± 1	1 ± 1
**Statistical parameter**		
R_{wp}	1.62	1.52
R_{exp}	0.59	0.60
GOF	2.73	2.53

§§ by the Rietveld refinement with the software *Topas V6* (*Bruker*) and the open source software *Profex* (*BGMN*).[29,30] The mineral composition of the crystalline phases in the sample Mexican iron ore is shown. For comparison with the other methods of XAFS and Mössbauer only the iron phases will be considered. The uncertainty of the mineral composition is given by 3 times e.s.d. according to ASTM E117-13.[31]

Mössbauer spectroscopy

Hyperfine parameters – Qualitative analysis

The Mössbauer raw data (including the fits) of the pure reference substances α-Fe_2O_3 and Fe_3O_4, the Mexican iron ore as well as the model mixtures of α-Fe_2O_3/Fe_3O_4 are available at the Zenodo repository.[17]

Table S11 Evaluated hyperfine parameters and subspectra areas of the reference substances and the model mixtures. §§

Sample	Signal shape	Isomer shift<br>δ / mm · s^{-1}	Quadrupole splitting<br>ΔE_Q / mm · s^{-1}	Magnetic splitting<br>H / T	Area<br>A / cts · mm · s^{-1}	Literature [33]
**Iron(III) oxide micro particles (α-Fe_2O_3)**	sextet	0.38482(85)	–0.10247(85)	51.6325(68)	269500(1900)	δ = 0.36 mm · s^{-1}<br>ΔE_Q = –0.19 mm · s^{-1}<br>H = 51.7 T<br>$f_{synthetic}$ = 0.811 – 0.860<br>$f_{natural}$ = 0.837 – 0.851
**Iron(II, III) oxide micro particles (Fe_3O_4)**	sextet 1 (A-site: Fe(III) in tetrahedral sites)	0.323(10)	–0.0009(86)	49.305(74)	59800(4500)	δ = 0.28 mm · s^{-1}<br>ΔE_Q = 0 mm · s^{-1}<br>H = 49.0 T<br>f = 0.889
	sextet 2 (B-site: Fe(II) and Fe(III) in octahedral sites)	0.671(16)	–0.012(15)	45.68(15)	59200(5800)	δ = 0.66 mm · s^{-1}<br>ΔE_Q = 0 mm · s^{-1}<br>H = 45.9 T<br>$f \approx$ 0.800
**α-Fe_2O_3/Fe_3O_4 30/70**	sextet 1: Fe_3O_4 (A-site)	0.322515*	–0.0113348*	50.2653*	80600(3600)	
	sextet 2: Fe_3O_4 (B-site)	0.691303*	–0.0114883*	46.3661*	39900(2600)	
	sextet 3: α-Fe_2O_3	0.392194*	–0.113659*	52.3525*	38000(1300)	
**α-Fe_2O_3/Fe_3O_4 50/50**	sextet 1: Fe_3O_4 (A-site)	0.3255*	–0.0113*	50.2650*	224700(4000)	
	sextet 2: Fe_3O_4 (B-site)	0.6913*	–0.0115*	46.3660*	114600(5500)	
	sextet 3: α-Fe_2O_3	0.392194*	–0.113659*	52.3525*	209600(3000)	
**α-Fe_2O_3/Fe_3O_4 70/30**	sextet 1: Fe_3O_4 (A-site)	0.27469*	0.0170128*	50.3516*	118100(4400)	
	sextet 2: Fe_3O_4 (B-site)	0.697952*	–0.0103262*	46.4436*	71100(3800)	
	sextet 3: α-Fe_2O_3	0.387914*	–0.116187*	52.3188*	323000(3700)	

§§ Measured at room temperature compared to selected representative literature values. The shown determined isomer shifts were corrected for the rhodium matrix of the used γ-source (calibration with α-Fe). Thus, the presented isomer shifts are relative to α-Fe. The values marked with * were obtained by a manual fitting procedure and were fixed, so uncertainty values cannot be indicated for these marked parameters. The literature Mössbauer fraction values f used in this work for the approximating quantification are also shown. As for the hyperfine parameters, f values at room temperature are displayed.

Table S12 Evaluated hyperfine parameters and subspectra areas of the reference substances and the iron ore sample. §§

Sample	Signal shape	Isomer shift δ / mm · s^{-1}	Quadrupole splitting ΔE_Q / mm · s^{-1}	Magnetic splitting H / T	Area A / cts · mm · s^{-1}	Literature [33]
**Iron(III) oxide micro particles (α-Fe_2O_3)**	sextet	0.38482(85)	–0.10247(85)	51.6325(68)	269500(1900)	δ = 0.36 mm · s^{-1}<br>ΔE_Q = –0.19 mm · s^{-1}<br>H = 51.7 T<br>$f_{synthetic}$ = 0.811 – 0.860<br>$f_{natural}$ = 0.837 – 0.851
**Iron(II, III) oxide micro particles (Fe_3O_4)**	sextet 1 (A-site: Fe(III) in tetrahedral sites)	0.323(10)	–0.0009(86)	49.305(74)	59800(4500)	δ = 0.28 mm · s^{-1}<br>ΔE_Q = 0 mm · s^{-1}<br>H = 49.0 T<br>f = 0.889
	sextet 2 (B-site: Fe(II) and Fe(III) in octahedral sites)	0.671(16)	–0.012(15)	45.68(15)	59200(5800)	δ = 0.66 mm · s^{-1}<br>ΔE_Q = 0 mm · s^{-1}<br>H = 45.9 T<br>$f \approx$ 0.800
**Mexican iron ore** §§§	sextet 1: Fe_3O_4 (A-site)	0.2463(65)	–0.0511(60)	49.761(46)	111220(6800)	
	sextet 2: Fe_3O_4 (B-site)	0.762923*	0.0274084*	47.3014*	105700(2400)	
	sextet 3: α-Fe_2O_3	0.3903(18)	–0.0986(18)	52.225(15)	212700(5600)	

§§ Measured at room temperature compared to selected representative literature values. The shown determined isomer shifts were corrected for the rhodium matrix of the used γ-source (calibration with α-Fe). Thus, the presented isomer shifts are relative to α-Fe. The values marked with * were obtained by a manual fitting procedure and were fixed, so uncertainty values cannot be indicated for these marked parameters. The literature Mössbauer fraction values f used in this work for the approximating quantification are also shown. As for the hyperfine parameters, f values at room temperature are displayed.
§§§ For Mexican iron ore hyperfine parameters δ, ΔE_Q and H see also [1].

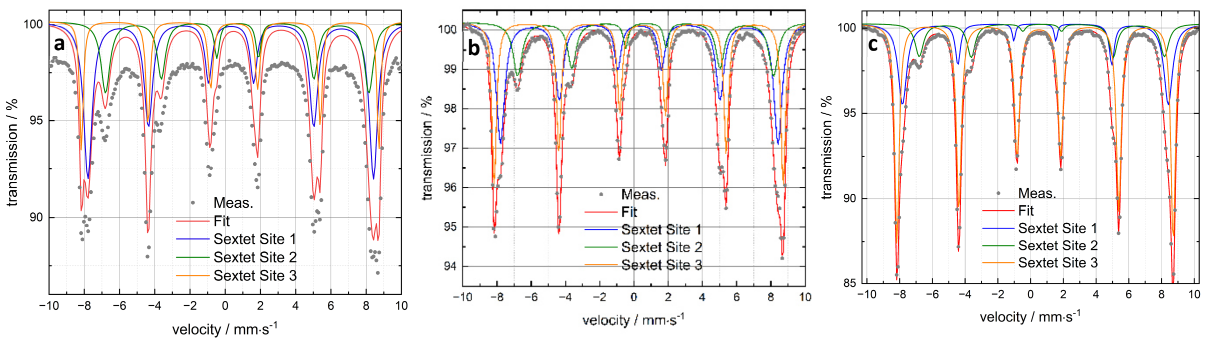

Fig S15 Mössbauer spectra of the α-Fe_2O_3/Fe_3O_4 model mixtures. **a** 30/70 ratio ($\chi_v^2 = \mathbf{1.429}$), **b** 50/50 ratio ($\chi_v^2 = \mathbf{2.692}$) and **c** 70/30 ratio ($\chi_v^2 = \mathbf{3.747}$). The total spectra fits are marked in red and the individual sextets in blue, green and orange. The measurement time was 23 h for 30/70, 120 h for 50/50 and 72 h for the 70/30 model mixture. The measurements were performed at room temperature.

Quantitative analysis – Model mixtures

General Mössbauer quantification formula (e.g., described in [33]):

$$\frac{N_{\mathrm{A}}}{N_{\mathrm{B}}} = \frac{n_{\mathrm{A}}}{n_{\mathrm{B}}} = \frac{A_{\mathrm{A}} \cdot f_{\mathrm{B}}}{A_{\mathrm{B}} \cdot f_{\mathrm{A}}} \tag{S4}$$

Here N_i is the amount of iron atoms of type i, n_i the amount of Mössbauer active iron atoms (^{57}Fe) of type i per area in the absorber, A_i the particular signal areas and f_i the Mössbauer fractions, respectively.[33]

Specific quantification formula in this study:

$$\frac{N_{\mathrm{Fe\ B-site}}}{N_{\mathrm{Fe\ \alpha-Fe2O3}}} = \frac{A_{\mathrm{Fe\ B-site}} \cdot f_{\mathrm{Fe\ \alpha-Fe2O3}}}{A_{\mathrm{Fe\ \alpha-Fe2O3}} \cdot f_{\mathrm{Fe\ B-site}}} \tag{S5a}$$

The Mössbauer fractions $f_{\mathrm{Fe\ \alpha\text{-}Fe2O3}}$ and $f_{\mathrm{Fe\ B\text{-}site}}$ were obtained by averaging literature values, because a detailed experimental determination of them was not accessible during the time of this work. The A-site contribution to the total iron atom amount was mathematically considered in later steps (see Table S13).

Table S13 Exemplary quantitative evaluation of the Mössbauer spectrum of the Model mixture ω_{rel}(α-Fe_2O_3)/ ω_{rel}(Fe_3O_4) 70/30. §§

Quantification step	Calculation process	Results
**1. Determination of *N*(Fe B-site)/*N*(Fe hematite)**	$\frac{N_{\text{Fe B-site}}}{N_{\text{Fe }\alpha-\text{Fe2O3}}} = \frac{A_{\text{Fe B-site}} \cdot f_{\text{Fe }\alpha-\text{Fe2O3}}}{A_{\text{Fe }\alpha-\text{Fe2O3}} \cdot f_{\text{Fe B-site}}}$<br><br>$f_{\text{Fe B-site}}$ = 0.80, $f_{\text{Fe }\alpha\text{-Fe2O3}} = \overline{f_{\text{Fe}_2\text{O}_3\text{ synthetic}}}$ = 0.8355 (cf. Table S11/12)	*N*(Fe B-site)/*N*(Fe α-Fe_2O_3) = 0.23 ± 0.01
**2. Conversion to amounts of substances *n*(Fe_3O_4) and *n*(α-Fe_2O_3)**	Assumption: 1 mol of total iron was considered in step 1<br><br>*n*(Fe B-site) = (0.19 ± 0.01) mol<br>*n*(Fe α-Fe_2O_3) = (0.81 ± 0.01) mol<br><br>Taking the Fe atoms of the A-site into account:<br><br>$n_{\text{Fe A-site}} = \frac{n_{\text{Fe B-site}}}{2/3} \cdot \frac{1}{3}$,<br>*n*(Fe A-site) = (0.09 ± 0.01) mol<br><br>$n_{\text{Fe magnetite total}} = n_{\text{Fe B-site}} + n_{\text{Fe A-site}}$<br><br>Calculating the amounts of substances:<br><br>$n(\text{Fe}_3\text{O}_4) = \frac{1}{3} \cdot n_{\text{Fe magnetite total}}$<br><br>$n(\text{Fe}_2\text{O}_3) = \frac{1}{2} \cdot n_{\text{Fe hematite}}$	*n*(Fe_3O_4) = (0.093 ± 0.004) mol<br><br>*n*(α-Fe_2O_3) = (0.407 ± 0.004) mol
**3. Calculation of the mass ratio ω_{rel}(Fe_3O_4)/ω_{rel}(α-Fe_2O_3)**	Determinations of the masses:<br><br>$m(\text{Fe}_3\text{O}_4) = M(\text{Fe}_3\text{O}_4) \cdot n(\text{Fe}_3\text{O}_4)$,<br>*m*(Fe_3O_4) = (21.64 ± 0.89) g<br><br>$m(\text{Fe}_2\text{O}_3) = M(\text{Fe}_2\text{O}_3) \cdot n(\text{Fe}_2\text{O}_3)$,<br>*m*(Fe_2O_3) = (64.92 ± 0.67) g<br><br>Calculating the relative mass fractions:<br><br>$\omega_{rel}(\text{Fe}_3\text{O}_4) = \left(\frac{m(\text{Fe}_3\text{O}_4)}{m(\text{Fe}_3\text{O}_4)+m(\text{Fe}_2\text{O}_3)}\right) \cdot 100\%$<br><br>$\omega_{rel}(\text{Fe}_2\text{O}_3) = \left(\frac{m(\text{Fe}_2\text{O}_3)}{m(\text{Fe}_3\text{O}_4)+m(\text{Fe}_2\text{O}_3)}\right) \cdot 100\%$	**ω_{rel}(α-Fe_2O_3) = (75.00 ± 0.76)%**<br><br>**ω_{rel}(Fe_3O_4) = (25.00 ± 1.03)%**<br><br>(via analogous calculation:<br>→ 30/70: ω_{rel}(α-Fe_2O_3) = (38.61 ± 1.48)% / ω_{rel}(Fe_3O_4) = (61.39 ± 3.38)%<br>→ 50/50: ω_{rel}(α-Fe_2O_3) = (54.71 ± 0.99)% / ω_{rel}(Fe_3O_4) = (45.29 ± 1.70)%)

§§ The indicated uncertainty values were obtained by the application of error propagation.

Quantitative analysis – Mexican iron ore

General Mössbauer quantification formula (e.g., described in [33]):

$$\frac{N_{\mathrm{A}}}{N_{\mathrm{B}}} = \frac{n_{\mathrm{A}}}{n_{\mathrm{B}}} = \frac{A_{\mathrm{A}} \cdot f_{\mathrm{B}}}{A_{\mathrm{B}} \cdot f_{\mathrm{A}}} \tag{S4}$$

Here N_i is the amount of iron atoms of type i, n_i the amount of Mössbauer active iron atoms (^{57}Fe) of type i per area in the absorber, A_i the particular signal areas and f_i the Mössbauer fractions, respectively.[33]

Specific quantification formula in this study:

$$\frac{N_{\mathrm{Fe\ B-site}}}{N_{\mathrm{Fe\ hematite}}} = \frac{A_{\mathrm{Fe\ B-site}} \cdot f_{\mathrm{Fe\ hematite}}}{A_{\mathrm{Fe\ hematite}} \cdot f_{\mathrm{Fe\ B-site}}} \tag{S5b}$$

The Mössbauer fractions $f_{\mathrm{Fe\ hematite}}$ and $f_{\mathrm{Fe\ B\text{-}site}}$ were obtained by averaging literature values, because a detailed experimental determination of them was not accessible during the time of this work. The A-site contribution to the total iron atom amount was mathematically considered in later steps (see Table S14).

Table S14 Quantitative evaluation of the Mössbauer spectrum of the Mexican iron ore. §§

**Quantification step**	**Calculation process**	**Results**
**1. Determination of *N*(Fe B-site)/*N*(Fe hematite)**	$\frac{N_{\text{Fe B-site}}}{N_{\text{Fe hematite}}} = \frac{A_{\text{Fe B-site}} \cdot f_{\text{Fe hematite}}}{A_{\text{Fe hematite}} \cdot f_{\text{Fe B-site}}}$<br><br>$f_{\text{Fe B-site}} = 0.80$, $f_{\text{Fe hematite}} = \overline{f_{\text{Fe}_2\text{O}_3\text{ natural}}} = 0.844$ (cf. Table 1)	*N*(Fe B-site)/*N*(Fe hematite) = 0.52 ± 0.02
**2. Conversion to amounts of substances *n*(Fe_3O_4) and *n*(α-Fe_2O_3)**	Assumption: 1 mol of total iron was considered in step 1<br><br>*n*(Fe B-site) = (0.34 ± 0.01) mol<br>*n*(Fe hematite) = (0.66 ± 0.01) mol<br><br>Taking the Fe atoms of the A-site into account:<br><br>$n_{\text{Fe A-site}} = \frac{n_{\text{Fe B-site}}}{2/3} \cdot \frac{1}{3}$,<br>*n*(Fe A-site) = (0.17 ± 0.01) mol<br><br>$n_{\text{Fe magnetite total}} = n_{\text{Fe B-site}} + n_{\text{Fe A-site}}$<br><br>Calculating the amounts of substances:<br><br>$n(\text{Fe}_3\text{O}_4) = \frac{1}{3} \cdot n_{\text{Fe magnetite total}}$<br><br>$n(\text{Fe}_2\text{O}_3) = \frac{1}{2} \cdot n_{\text{Fe hematite}}$	*n*(Fe_3O_4) = (0.172 ± 0.004) mol<br><br>*n*(α-Fe_2O_3) = (0.328 ± 0.004) mol
**3. Calculation of the mass ratio $\omega_{rel}(Fe_3O_4)/\omega_{rel}(\alpha\text{-}Fe_2O_3)$**	Determinations of the masses:<br><br>$m(\text{Fe}_3\text{O}_4) = M(\text{Fe}_3\text{O}_4) \cdot n(\text{Fe}_3\text{O}_4)$,<br>*m*(Fe_3O_4) = (39.82 ± 1.03) g<br><br>$m(\text{Fe}_2\text{O}_3) = M(\text{Fe}_2\text{O}_3) \cdot n(\text{Fe}_2\text{O}_3)$,<br>*m*(Fe_2O_3) = (52.38 ± 0.63) g<br><br>Calculating the relative mass fractions:<br><br>$\omega_{rel}(\text{Fe}_3\text{O}_4) = \left(\frac{m(\text{Fe}_3\text{O}_4)}{m(\text{Fe}_3\text{O}_4)+m(\text{Fe}_2\text{O}_3)}\right) \cdot 100\%$<br><br>$\omega_{rel}(\text{Fe}_2\text{O}_3) = \left(\frac{m(\text{Fe}_2\text{O}_3)}{m(\text{Fe}_3\text{O}_4)+m(\text{Fe}_2\text{O}_3)}\right) \cdot 100\%$	**ω_{rel}(α-Fe_2O_3) = (56.81 ± 0.67)%**<br><br>**ω_{rel}(Fe_3O_4) = (43.19 ± 1.11)%**

§§ The indicated uncertainty values were obtained by the application of error propagation.

Compact overview

Table S15 General differences of the three techniques for quantitative analysis.

XAFS	Mössbauer	XRD
**-** sample effects e.g., thickness, homogeneity	**-** sample effects e.g., thickness, homogeneity	**-** texture effects, homogeneity
- up to 4 species	- strongly depending on hyperfine parameters	- works for up to 8 species
- all phases	- solid phases	- solid crystalline phases
- particle size < μ^{-1}(absorption length) [5]	- particle size > nm (limitation in 10 nm range [34])	- particle size > nm (X-ray amorphous below 3-15 nm)
- few databases with sometimes strong influence of specific spectrometer, therefore need of reference materials and more pre-knowledge of sample	- databases (especially for Fe-compounds) existent (but to a lesser extent compared to XRD & often wide ranges of the hyperfine parameters of a species)	- huge database(s)
- accessible elements limited by spectral range of spectrometer	- few elements in PSE	- all crystals
- strong influence of human interaction in data pre-processing	- complex quantification with a lot of assumptions or higher experimental expenditure	- complex quantification with a lot of assumptions or higher experimental expenditure

Data availability

Data for this article, including the raw data files of the XAS, XRD and Mössbauer measurements are available at Zenodo at https://zenodo.org/doi/10.5281/zenodo.14959531.[17]

References

1. Motz DA. Entwicklung von Referenzmaterialien für die Röntgen-Nahkanten-Absorptionsspektroskopie am Laboraufbau. Hannover : Institutionelles Repositorium der Leibniz Universität Hannover; 2021. doi:10.15488/10431

2. Markl G. Minerale und Gesteine: Mineralogie, Petrologie, Geochemie. 2., verb. und erw. Aufl., durchges. Nachdr. Heidelberg: Spektrum Akademischer Verlag; 2012.

3. Neukirchen F, Ries G. Die Welt der Rohstoffe: Lagerstätten, Förderung und wirtschaftliche Aspekte. Berlin: Springer Spektrum; 2014.

4. Okrusch M, Matthes S. Mineralogie. Berlin, Heidelberg: Springer Berlin Heidelberg; 2014. doi:10.1007/978-3-642-34660-6

5. Bunker G. Introduction to XAFS: A practical guide to X-ray absorption fine structure spectroscopy. Cambridge and New York: Cambridge University Press; 2010. Available: https://search.ebscohost.com/login.aspx?direct=true&scope=site&db=nlebk&db=nlabk&AN=324079

6. Chantler CT, Olsen K, Dragoset RA, Chang J, Kishore AR, Kotochigova SA, et al. X-Ray Form Factor, Attenuation, and Scattering Tables (version 2.1.). National Institute of Standards and Technology. 2005 [cited 28 Feb 2025]. Available: https://www.nist.gov/pml/x-ray-form-factor-attenuation-and-scattering-tables

7. DIN 38402-51:1986, Deutsche Einheitsverfahren zur Wasser-, Abwasser- und Schlammuntersuchung_- Allgemeine Angaben (Gruppe_A)_- Teil_51: Kalibrierung von Analysenverfahren_- Lineare Kalibrierfunktion_(A_51). Berlin: Beuth Verlag GmbH; doi:10.31030/2657448

8. DIN 32645:2008-11, Chemische Analytik_- Nachweis-, Erfassungs- und Bestimmungsgrenze unter Wiederholbedingungen_- Begriffe, Verfahren, Auswertung. Berlin: Beuth Verlag GmbH; doi:10.31030/1465413

9. Honeywell/Fluka. iron(III) oxide (310050), powder, <5µm, ≥99%. Honeywell, editor. Available: https://lab.honeywell.com/shop/iron-iii-oxide-310050

10. Sigma-Aldrich. iron(II,III) oxide (310069): powder, < 5µm, 95%. Available: https://www.sigmaaldrich.com/DE/de/product/aldrich/310069

11. Stoe & Cie, editor. WINXPOW. Darmstadt, Germany; 2004.

12. Petrovský E, Kropáček V, Dekkers MJ, deBoer C, Hoffmann V, Ambatiello A. Transformation of hematite to maghemite as observed by changes in magnetic parameters: Effects of mechanical activation? Geophysical Research Letters. 1996;23: 1477–1480. doi:10.1029/96GL01411

13. Choudhary A, Khandelwal N, Ganie ZA, Darbha GK. Influence of magnetite and its weathering originated maghemite and hematite minerals on sedimentation and transport of nanoplastics in the aqueous and subsurface environments. Science of The Total Environment. 2024;912: 169132. doi:10.1016/j.scitotenv.2023.169132

14. Cornell RM, Schwertmann U. The Iron Oxides: Structure, Properties, Reactions, Occurences and Uses. 1st ed. Wiley; 2003. doi:10.1002/3527602097

15. Ravel B, Newville M. ATHENA, ARTEMIS, HEPHAESTUS: data analysis for X-ray absorption spectroscopy using IFEFFIT. Journal of synchrotron radiation. 2005;12: 537–541. doi:10.1107/S0909049505012719

16. Matthew Newville. Larch: An Analysis Package for XAFS and Related Spectroscopies. Journal of Physics: Conference Series. 2013;430: 012007. doi:10.1088/1742-6596/430/1/012007

17. Praetz S, Schlesiger C, Motz DA, Heinrich L, Heppke EM, Klimke S, et al. Data set from: Can laboratory-based XAFS compete with XRD and Mössbauer spectroscopy as a tool for quantitative species analysis? Zenodo; 2025. doi:10.5281/ZENODO.14959531

18. Schlesiger C, Anklamm L, Stiel H, Malzer W, Kanngießer B. XAFS spectroscopy by an X-ray tube based spectrometer using a novel type of HOPG mosaic crystal and optimized image processing. Journal of Analytical Atomic Spectrometry. 2015;30: 1080–1085. doi:10.1039/C4JA00303A

19. Calvin S, Furst KE. XAFS for everyone. Boca Raton, Fla.: CRC Press; 2013.

20. Bevington PR, Robinson DK. Data reduction and error analysis for the physical sciences. 3. ed., [Nachdr.]. Boston: McGraw-Hill; 2010.

21. Sigma-Aldrich. Iron(III) oxide (544884: nanopowder, < 50 nm particle size (BET). Available: https://www.sigmaaldrich.com/DE/de/product/aldrich/544884

22. Schmitt B, Bollard P, Albert D, Garenne A, Gorbacheva M, Bonal L, et al. SSHADE: \textquotedblSolid Spectroscopy Hosting Architecture of Databases and Expertise\textquotedbl. OSUG Data Center; 2017. doi:10.26302/SSHADE

23. Ould-Chikh S, Vollmer I, Aguilar Tapia A. Fe K edge XAS HERFD (Kbeta1,3) and XES of synthetic maghemite gamma-Fe2O3 at ambient conditions. SSHADE/FAME (OSUG Data Center); 2018. doi:10.26302/SSHADE/EXPERIMENT_ SOC_ 20181115_ 005

24. Testemale D. Fe K edge XAS transmission of natural magnetite Fe3O4 at ambient conditions. SSHADE/FAME (OSUG Data Center); 2018. doi:10.26302/SSHADE/EXPERIMENT_ DT_ 20170704_ 001

25. Testemale D, Sanchez-Valle C. Fe K edge XAS transmission of natural hematite Fe2O3 at ambient conditions. SSHADE/FAME (OSUG Data Center); 2018. doi:10.26302/SSHADE/EXPERIMENT_ DT_ 20170706_ 002

26. Kuzmann E, Nagy S, Vértes A. Critical review of analytical applications of Mössbauer spectroscopy illustrated by mineralogical and geological examples (IUPAC Technical Report). Pure and Applied Chemistry. 2003;75: 801–858. doi:10.1351/pac200375060801

27. Rietveld HM. A profile refinement method for nuclear and magnetic structures. Journal of applied crystallography. 1969;2: 65–71. doi:10.1107/S0021889869006558

28. Rodríguez-Carvajal J. FULLPROF: a program for Rietveld refinement and pattern matching analysis. Toulouse, France: Satellite Meeting on Powder Diffraction of the XV Congress of the IUCr; 1990.

29. Bergmann J, P. Friedel P, Kleeberg R. BGMN – a new fundamental parameter based Rietveld program for laboratory X-ray sources, its use in quantitative analysis and structure investigations. International Union of Crystallography. 1998; 5-ß.

30. Doebelin N, Kleeberg R. Profex: a graphical user interface for the Rietveld refinement program BGMN. Journal of applied crystallography. 2015;48: 1573–1580. doi:10.1107/S1600576715014685

31. E11 Committee. Practice for Use of the Terms Precision and Bias in ASTM Test Methods. West Conshohocken, PA: ASTM International; doi:10.1520/E0177-13

32. Runčevski T. Rietveld Refinement Practical Powder Diffraction Pattern Analysis using TOPAS. By Robert E. Dinnebier, Andreas Leineweber and John S. O. Evans. De Gruyter, 2019. Pp. 331. Price (paperback) EUR 69.95, USD 80.99, GBP 63.50. ISBN 978-3-11-045621-9, e-ISBN (PDF) 978-3-11-046138-1. Journal of applied crystallography. 2019;52: 1238–1239. doi:10.1107/S1600576719011178

33. Vandenberghe RE, de Grave E. Application of Mössbauer Spectroscopy in Earth Sciences. In: Yoshida Y, Langouche G, editors. Mössbauer Spectroscopy. Berlin, Heidelberg: Springer Berlin Heidelberg; 2013. pp. 91–185. doi:10.1007/978-3-642-32220-4_ 3

34. Enver Murad, Udo Schwertmann. The Möessbauer spectrum of ferrihydrite and its relations to those of other iron oxides. American Mineralogist. 1980;65: 1044–1049.
